# Targeting Glial Cells by Organic Anion-Transporting Polypeptide 1C1 (OATP1C1)-Utilizing l-Thyroxine-Derived Prodrugs

**DOI:** 10.1021/acs.jmedchem.3c01026

**Published:** 2023-11-06

**Authors:** Arun Kumar Tonduru, Seyed Hamed Maljaei, Santosh Kumar Adla, Landry Anamea, Janne Tampio, Adéla Králová, Aaro J. Jalkanen, Catarina Espada, Inês Falcato Santos, Ahmed B. Montaser, Jarkko Rautio, Thales Kronenberger, Antti Poso, Kristiina M. Huttunen

**Affiliations:** † School of Pharmacy, Faculty of Health Sciences, University of Eastern Finland, P.O. Box 1627, 70211 Kuopio, Finland; ‡ Department of Pharmaceutical and Medicinal Chemistry, Institute of Pharmaceutical Sciences, Eberhard-Karls-Universität, Tuebingen, Auf der Morgenstelle 8, 72076 Tuebingen, Germany; § Tuebingen Center for Academic Drug Discovery & Development (TüCAD2), 72076 Tuebingen, Germany; ∥ Department of Internal Medicine VIII, University Hospital Tübingen, DE 72076 Tübingen, Germany; ⊥ Cluster of Excellence iFIT (EXC 2180) “Image-Guided and Functionally Instructed Tumor Therapies”, University of Tübingen, 72076 Tübingen, Germany

## Abstract

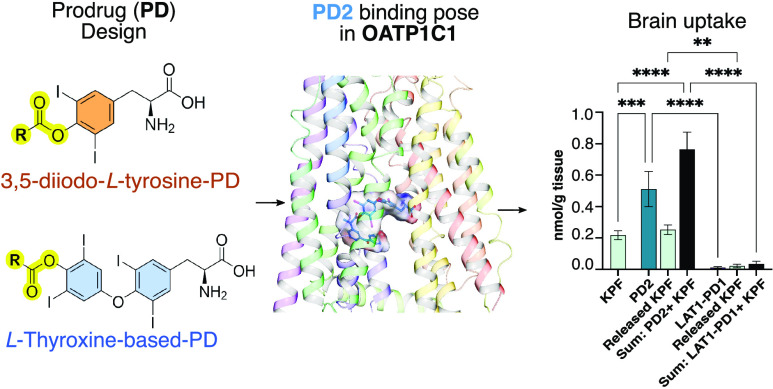

OATP1C1 (organic anion-transporting polypeptide 1C1) transports thyroid hormones, particularly thyroxine (T_4_), into human astrocytes. In this study, we investigated the potential of utilizing OATP1C1 to improve the delivery of anti-inflammatory drugs into glial cells. We designed and synthesized eight novel prodrugs by incorporating T_4_ and 3,5-diiodo-l-tyrosine (DIT) as promoieties to selected anti-inflammatory drugs. The prodrug uptake in OATP1C1-expressing human U-87MG glioma cells demonstrated higher accumulation with T_4_ promoiety compared to those with DIT promoiety or the parent drugs themselves. *In silico* models of OATP1C1 suggested dynamic binding for the prodrugs, wherein the pose changed from vertical to horizontal. The predicted binding energies correlated with the transport profiles, with T_4_ derivatives exhibiting higher binding energies when compared to prodrugs with a DIT promoiety. Interestingly, the prodrugs also showed utilization of oatp1a4/1a5/1a6 in mouse primary astrocytes, which was further supported by docking studies and a great potential for improved brain drug delivery.

## Introduction

OATP1C1 (organic anion-transporting polypeptide 1C1) is an organic anion-transporting polypeptide that belongs to the solute carrier family (SLCO1C1, also known as OATP14 or OATP-F or SLC21A14) and is a primary thyroid hormone transporter. It was characterized as a sodium-independent transporter that was originally localized to the brain and testes.^1^ In the human fetal brain, OATP1C1, is expressed in astrocytes and glial cells.^2^ It plays a crucial role in transporting thyroid hormones (TH), the prohormone thyroxine (T_4_), and reverse triiodothyronine (rT_3_) with high affinity (Michaelis constant *K*_m_ in the nanomolar range) compared to other OATPs.^1^ The essential role of thyroid hormones in brain development and function is demonstrated by the severe consequences observed in congenital hypothyroidism.^3^ OATP1C1 facilitates the uptake of T_4_ into astrocytes, while monocarboxylate transporter 8 (MCT8) mediates the uptake of T_4_ across the blood–brain barrier (BBB).^4^ The Asp252Asn mutation in OATP1C1 has led to intracellular retention of the transporter, which in turn has caused reduced uptake of T_4_ into astrocytes and hindered its conversion to triiodothyronine (T_3_).^5^ OATP1C1 also transports several other substrates like bromosulfophthalein (BSP), estrone-3-sulfate (ES), and estradiol 17β-d-glucuronide (E_2_17G) with lower efficiency.^1^ Site-directed mutagenesis experiments have revealed that OATP1C1 transport of T_4_ and ES is pH independent as it lacks the conserved histidine on transmembrane domain 3, contradicting other OATPs.^6^

OATP1C1 transport exhibits atypical kinetics, indicating the presence of multiple binding sites, which is also observed in other members of the OATP family.^7^ The identification of binding site residues and residues lining the putative pore is essential to understanding the structure and function of the transporter protein. Since the experimental structure of the OATP1C1 is unavailable, comparative modeling has been employed to predict the structure and comprehend the transport of substrates. The predicted structure of OATP1C1 consists of 12 transmembrane helices, with N and C terminals located in the cytoplasm, similar to other major facilitator superfamily (MFS) members.^8^ It is proposed that OATPs, like other MFS members, follow a “Rocker-switch” transport mechanism, in which they alternate between outward and inward conformations.^9,10^ Numerous studies have reported homology models of OATPs with binding site predictions and evaluation of mutagenic residues involved in the transport of substrates.^5,11−18^ Molecular dynamics (MD) investigations using the homology model of OATP1C1 have helped to elucidate the underlying mechanism involved in the mutation of Asp252Asn in the transmembrane domain 5.^5^ The transport of T_4_ was either abolished or diminished when mutations are applied on amino acid residues Trp277, Trp278, Arg601, and Pro609 in rat oatp1c1.^17^

**Figure 1:**
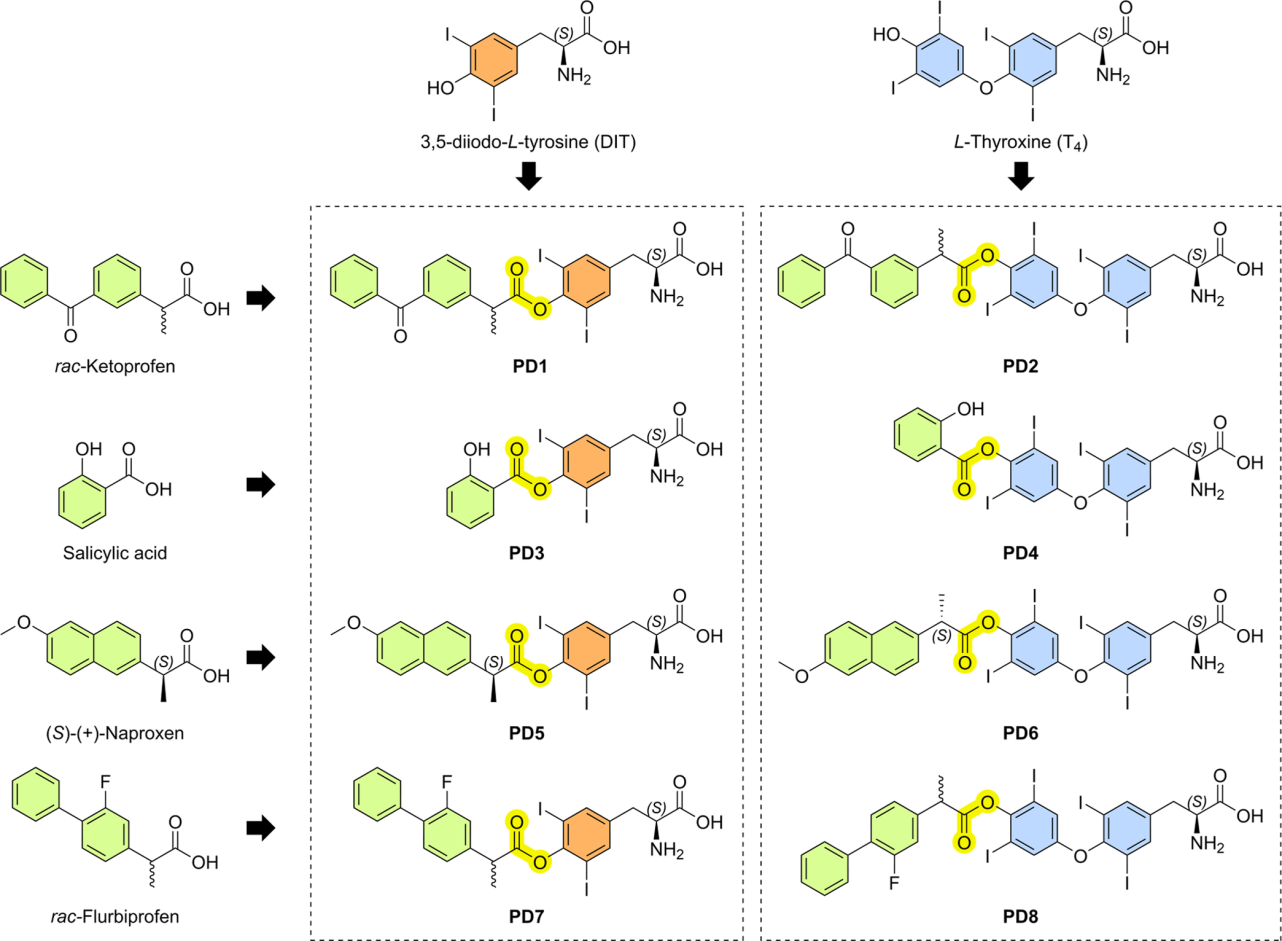
Structures of DIT and T_4_ prodrugs along with the parent NSAID drugs.

Since the BBB restricts efficient drug delivery into the brain, various methods, such as nanocarriers and prodrugs, have been extensively investigated in the past. We have utilized l-type amino acid transporter 1 (LAT1) to improve brain drug delivery of several nonsteroidal anti-inflammatory drugs (NSAIDs), namely, ketoprofen, salicylic acid, flurbiprofen, ibuprofen, and naproxen, in the form of amino acid prodrugs.^19−25^ The aim of the selected anti-inflammatory drugs has been to reduce neuroinflammation behind many neurodegenerative diseases. However, due to LAT1 being a high affinity, low-capacity transporter expressed in neurons and glial cells (astrocytes and microglia), we sought alternative approaches to achieve higher capacity in delivery and preferable selectivity toward glial cells. Therefore, in the present study, we designed and synthesized eight prodrugs (PDs) that would utilize OATP1C1 for their cellular internalization and astrocyte-targeting. Given that T_4_ is a known substrate for OATP1C1, it was used as a backbone for the design of novel compounds (Figure 1). The AlphaFold model of OATP1C1 was used for docking and MD simulations to generate potential protein–ligand binding modes. Finally, we investigated the cellular accumulation of the novel prodrugs mediated by OATP and the delivery of their parent drugs in human U-87MG glioma cells and mouse primary astrocytes.

## Results and Discussion

### Synthesis of Novel OATP-Utilizing Prodrugs

**Scheme 1:**
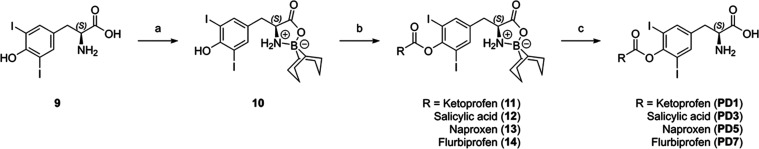
Synthesis of DIT-Based Prodrugs Reagents and conditions: (a) 9-BBN (0.5 M solution in tetrahydrofuran (THF)), THF, room temperature, 3 days, 97%; (b) i. EDC·HCl, DMAP, CH_2_Cl_2_/DMF, reflux, 3 days; ii. EDC·HCl, DMAP, DMF, microwave, 100 °C, 30 min, 41–79%; (c) i. *tert*-butyl hydroperoxide, CHCl_3_/MeOH, room temperature, open air, no septum, 4 days; ii. HCl 1 M, CH_2_Cl_2_/MeOH, microwave, 100 °C, 30–60 min, 48–66%.

**Scheme 2:**
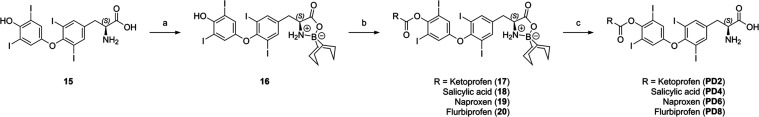
Synthetic Route for the T_4_ Ester Prodrugs Reagents and conditions: (a) 9-BBN (0.5 M solution in THF), THF, room temperature, 3 days, 98%; (b) i. EDC·HCl, DMAP, CH_2_Cl_2_/DMF, reflux, 3 days; ii. EDC·HCl, DMAP, DMF, microwave, 100 °C, 30 min, 46–94%; (c) i. *tert*-butyl hydroperoxide, CHCl_3_/MeOH, room temperature, open air, no septum, 4 days; ii. HCl 1 M, CH_2_Cl_2_/MeOH, microwave, 100 °C, 30–60 min, 33–99%.

To increase the selective cellular accumulation of anti-inflammatory drugs into astrocytes via OATP1C1, prodrugs **1**–**8** were designed and confirmed by computational modeling. All prodrugs were synthesized based on the literature procedures^19,26,27^ with extensive optimization and developing methods using a microwave synthesizer. In brief, a 9-BBN-protected ligand, here DIT (as a synthetic ligand; Scheme 1) or T_4_ (as a natural ligand; Scheme 2), and four NSAIDs (ketoprofen, flurbiprofen, naproxen, and salicylic acid) were coupled together (either via method A, refluxing together with EDC·HCl and 4-dimethylaminopyridine (DMAP) in CH_2_Cl_2_/*N*,*N*-dimethylformamide (DMF) solution, or method B, stirring in DMF with the EDC·HCl and DMAP). The NSAIDs selected for this study were the same that we have used in the past in order to compare the current OATP-prodrug strategy to the LAT1-prodrug approach. Then, the deprotection of the 9-BBN moiety was performed by either an oxidative cleavage reaction at room temperature (method C) or a quick reaction with 1 M aq. HCl in a microwave reactor (method D).

### Stability and Bioconversion of Novel OATP-Utilizing Prodrugs

Chemical stability and enzymatic bioconversion of prepared prodrugs (100 μM) were studied in Tris buffer at pH 7 at +37 °C and in human plasma and liver S9 subcellular fraction as well as in mouse serum, liver S9 fraction, and astrocyte–microglia cell homogenate (Tables 1–2). All of the studied prodrugs were stable in Tris buffer for 24 h, and hence, they were chemically stable. Ketoprofen prodrugs (**1** and **2**) also showed good stability in all studied biological media. Curiously, for both prodrugs, the bioconversion rates were very similar in the mouse and human-derived media (Table 1). Moreover, their stability in mouse serum/human plasma was close to their stability in mouse brain cells. Lower stability (higher bioconversion rate) was observed in the mouse and human liver, which can be attributed to the higher presence of *e.g.*, carboxylesterase 1 (CES1). However, the overall stability of prodrug **2** (a T_4_ derivative) was a little more than prodrug **1** (a DIT derivative).

**Table 1:** Enzymatic Stability of Prodrugs **1**–**8** in Mouse and Human Serum/Plasma, Mouse, and Human Liver S9 Fraction, as well as in Mouse Brain (Presented as Remaining Percentages, %), Measured after 5 h Incubation^a^

prodrug	mouse liver S9	human liver S9	mouse serum	human plasma	mouse brain cell homogenate
**1**	74.30 ± 7.50	78.13 ± 2.17	94.32 ± 1.27	92.01 ± 5.02	85.76 ± 2.74
**2**	87.59 ± 1.20	85.71 ± 4.27	98.79 ± 1.21	96.47 ± 3.53	96.01 ± 3.99
**3**	99.06 ± 0.94	96.84 ± 3.16	14.40 ± 0.60	68.94 ± 2.01	95.26 ± 4.74
**4**	69.18 ± 0.81	84.00 ± 3.90	96.79 ± 3.21	97.17 ± 2.83	91.38 ± 5.34
**5**	38.26 ± 4.49	69.44 ± 2.10	96.75 ± 3.25	70.94 ± 29.06	92.98 ± 6.14
**6**	78.10 ± 1.88	78.70 ± 3.74	98.33 ± 1.67	96.32 ± 3.68	95.18 ± 4.82
**7**	25.19 ± 6.22	30.54 ± 3.21	94.89 ± 5.11	90.52 ± 9.48	92.68 ± 7.32
**8**	84.32 ± 0.99	80.17 ± 2.23	96.79 ± 3.21	97.17 ± 2.83	97.94 ± 2.06

a The data are expressed as mean ± standard deviation (SD), *n* = 3.

**Table 2:** Enzymatic Bioconversion of Prodrugs **3**, **5**, and **7** in Mouse Serum and Liver S9 Fraction, as well as in Human Liver S9 Fraction, Presented as Half-Lives, *t*_1/2_ (Min; Mean ± SD, *n* = 3)

prodrug	matrix	*t*_1/2_ (min)
PD3	mouse serum	83.33 ± 29.43
PD5	mouse liver	249.16 ± 45.63
PD7	mouse liver	161.11 ± 25.76
PD7	human liver	174.52 ± 15.01

For salicylic acid prodrugs (**3** and **4**), the bioconversion rate of prodrug **4** in human and mouse liver S9 fraction was more effective than prodrug **3** (Table 1). However, prodrug **3** exhibited a 5 times higher bioconversion rate in mouse serum than human plasma (Table 2), whereas prodrug **4** showed a slower and similar bioconversion rate in these two mediums. One probable explanation for this observation is that prodrug **3** is sensitive to hydrolyzing enzymes, which are available in mouse serum but have not been detected in human plasma, such as specific subforms of CES1. Contrary to other pairs of prodrugs, bioconversion of prodrug **4** in the mouse brain cell homogenate occurred faster than prodrug **3**. Similarly to ketoprofen prodrugs, the DIT derivative of naproxen, prodrug **5**, showed a 2-times faster bioconversion rate in mouse liver S9 fraction than its T_4_ derivative, prodrug **6** (Tables 1–2). The bioconversion rate of prodrug **5** in the human liver was also around 10% slower than that of prodrug **6**. Both prodrugs showed very high stability in mouse serum, human plasma, and mouse brain cell homogenate, suggesting that bioconversion occurs mainly via liver-specific enzymes.

A similar bioconversion pattern to each other was also observed with naproxen and flurbiprofen prodrugs in mouse serum, human plasma, and mouse brain cell homogenate (Tables 1–2). The bioconversion rate of DIT-flurbiprofen prodrug **7** in mouse and human liver was around 3- and 2.5-times faster than T_4_-flurbiprofen prodrug **8**. Taken together, it can be concluded from this study that the T_4_ derivatives of ketoprofen, naproxen, and flurbiprofen displayed higher stability than their corresponding DIT derivatives in all mediums. Contrarily, the T_4_ derivative of salicylic acid has a lower stability than its DIT derivative. However, all of these studied prodrugs with ester prodrug bonds showed higher enzymatic stability than, *e.g.*, previously reported corresponding LAT1-utilizing prodrugs that are much smaller in their overall molecular weight.^25,27^ Thus, the electronic and steric hindrance imposed by iodines near the prodrug bond is most likely to impede the bioconversion rate, as they can prevent the serine residues of the bioconverting enzymes from attacking the carbonyl carbon of the ester bond. However, since overall the DIT derivatives were bioconverted more readily than their corresponding T4 derivatives, the prodrug size may also have an impact on the bioconversion rate, as the larger prodrugs can be diverted away from the enzymes’ active pockets.

### Human U-87MG Glioma Cells and Mouse Primary Astrocytes Express Functional OATPs on Their Plasma Membrane

**Figure 2:**
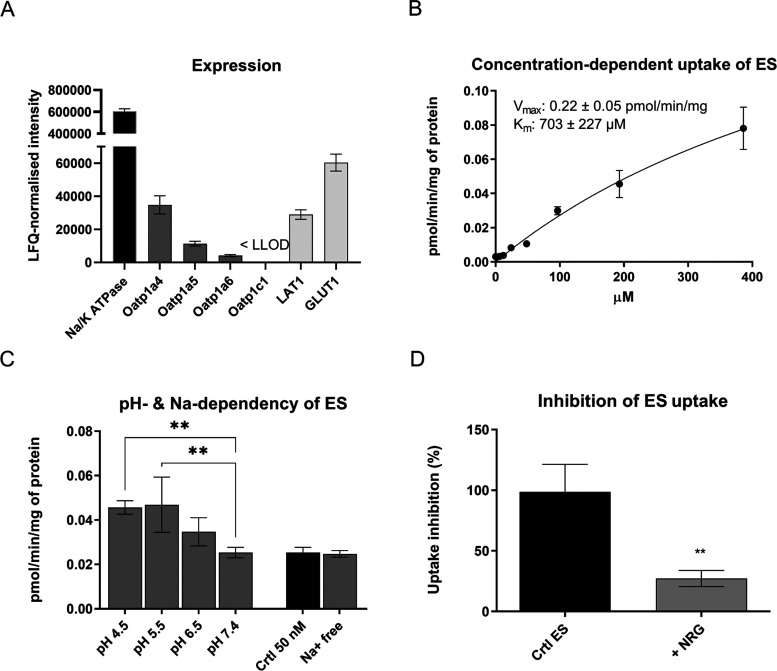
(A) Relative expression of organic anion-transporting polypeptide 1c1 (below the lowest limit of detection, LLOD) together with l-type amino acid transporter 1 (LAT1), glucose transporter 1 (GLUT1), and sodium–potassium adenosine triphosphatase (Na^+^/K^+^-ATPase) measured by a nontargeted global proteomic approach from mouse primary astrocytes and normalized to the total amount of protein in the plasma membrane (mean ± SD, *n* = 3). (B) Concentration-dependent cellular uptake of a known OATP substrate [6,7-^3^H(*N*)]-estrone-3-sulfate (ES) uptake (5–400 μM) into mouse primary astrocytes. (C) pH-Dependent uptake (4.5–8.5) of ES in the presence of Na^+^ (left) and sodium-independent uptake at pH 7.4 (right; Hank’s Balanced Salt Solution (HBSS) buffer with and without sodium ions) in mouse primary astrocytes. (D) Cellular uptake of ES (100 μM) in the presence of the OATP1A2 inhibitor (100 μM), naringin (NRG), in mouse primary astrocytes. All data are presented as mean ± SD (*n* = 3; biological replicates), and an asterisk denotes a statistically significant difference from the respective control uptake (black bars) (** *P* < 0.01, one-way analysis of variance (ANOVA), followed by Dunnett’s multiple comparison test).

**Figure 3:**
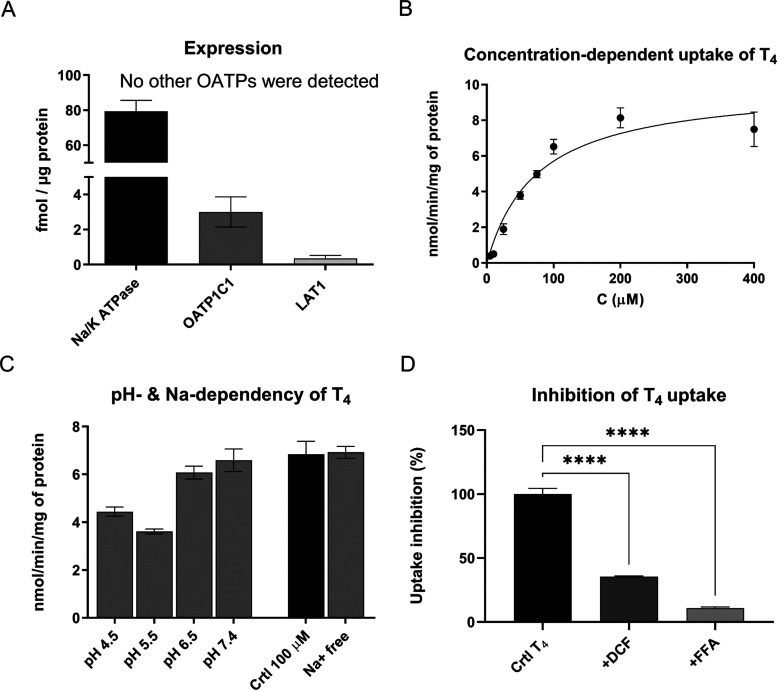
(A) Quantitative protein levels of organic anion-transporting polypeptide 1C1 (others were not detected) together with l-type amino acid transporter 1 (LAT1) and sodium–potassium adenosine triphosphatase (Na^+^/K^+^-ATPase) were analyzed from the plasma membranes of human glioma cells (U-87MG) and normalized to the total amount of protein in the plasma membrane. (B) Concentration-dependent cellular uptake of a known OATP1C1 substrate, thyroxine (T_4_), uptake (5–400 μM) into human glioma U-87MG cells. (C) pH-Dependent uptake (4.5–8.5) of T_4_ in the presence of Na+ (left) and sodium-independent uptake at pH 7.4 (right; HBSS buffer with and without sodium ions) in human U-87MG glioma cells. (D) Cellular uptake of T_4_ (100 μM) in the presence of OATP1C1 inhibitors or competing substrates (100 μM), diclofenac (DFC) and flufenamic acid (FFA), in human U-87MG glioma cells. All data are presented as mean ± SD (*n* = 3; biological replicates), and an asterisk denotes a statistically significant difference from the respective control uptake (black bars) (** *P* < 0.01, *** *P* < 0.001, one-way ANOVA, followed by Dunnett’s multiple comparison test).

The cellular accumulation into the astrocytes and the OATP1C1-mediated cellular uptake was studied in mouse primary astrocytes and human glioma U-87MG cells. Since mouse primary astrocytes did not express oatp1c1, but other oatp subtypes (oatp1a4, 1a5, and 1a6; Figure 2A), the interactions of the prepared prodrugs were first studied in human U-87MG cells that expressed OATP1C1, but not other OATPs. Notably, U-87MG cells expressed OATP1C1 with over 8 times higher extent, *e.g.*, compared to highly expressed l-type amino acid transporter 1 (LAT1; 3.01 ± 0.85 vs 0.36 ± 0.16 fmol/μg protein, respectively; Figure 3A). The functionality of expressed OATPs/oatps (here on capital OATP is used for human proteins and a small letter “oatp” for mouse or other animals) was also different; in U-87MG cells, the probe substrate, T_4_, saturated and followed Michaelis–Menten kinetics, while in astrocytes several transport mechanisms (distinct oatp subtypes) participated in the uptake of estrone-3-sulfate (ES), which confused the interpretation, creating almost linear uptake curve (Figures 2B and 3B). However, according to the pH-dependent uptake (Figures 2C and 3C), it was evident that the transport of T_4_ was mainly carried out by pH-insensitive subtypes, such as the uptake of OATP1C1 into the U-87MG cells, whereas the uptake of ES into the primary astrocytes was mediated via pH-sensitive subtypes, such as oatp1a4, 1a5, and 1a6. Mutational studies have revealed that T_4_ uptake by OATP1C1 was pH insensitive due to the lack of conserved histidine on TM3. In the same study, mutating His107 to Gln on rat oatp1a1 resulted in the loss of pH sensitivity, whereas in human OATP1C1, the pH sensitivity was attained when Gln130 was mutated to histidine.^6^ This made it evident that the conserved histidine present on TM3 was critical for the pH sensitivity of the OATPs. Although pH insensitivity was identified, Patik et al. (2015) reported that OATP1C1-mediated transport was activated by lower pH. This discrepancy was explained by the different substrates used in the experiments.^28^ Nevertheless, the lack of sodium-dependency and inhibition of T_4_ uptake by known OATP1C1 inhibitors, diclofenac (DCF) and flufenamic acid (FFA), as well as inhibition of ES by a known OATP1A2 inhibitor naringin (NRG) proved that human U-87MG glioma cells expressed functional OATP1C1 and mouse primary astrocytes expressed other oatp1a-subtypes on their plasma membranes (Figures 2D and 3D).^29^

### Novel Prodrugs can Accumulate into Human U-87MG Glioma Cells via OATP1C1

**Figure 4:**
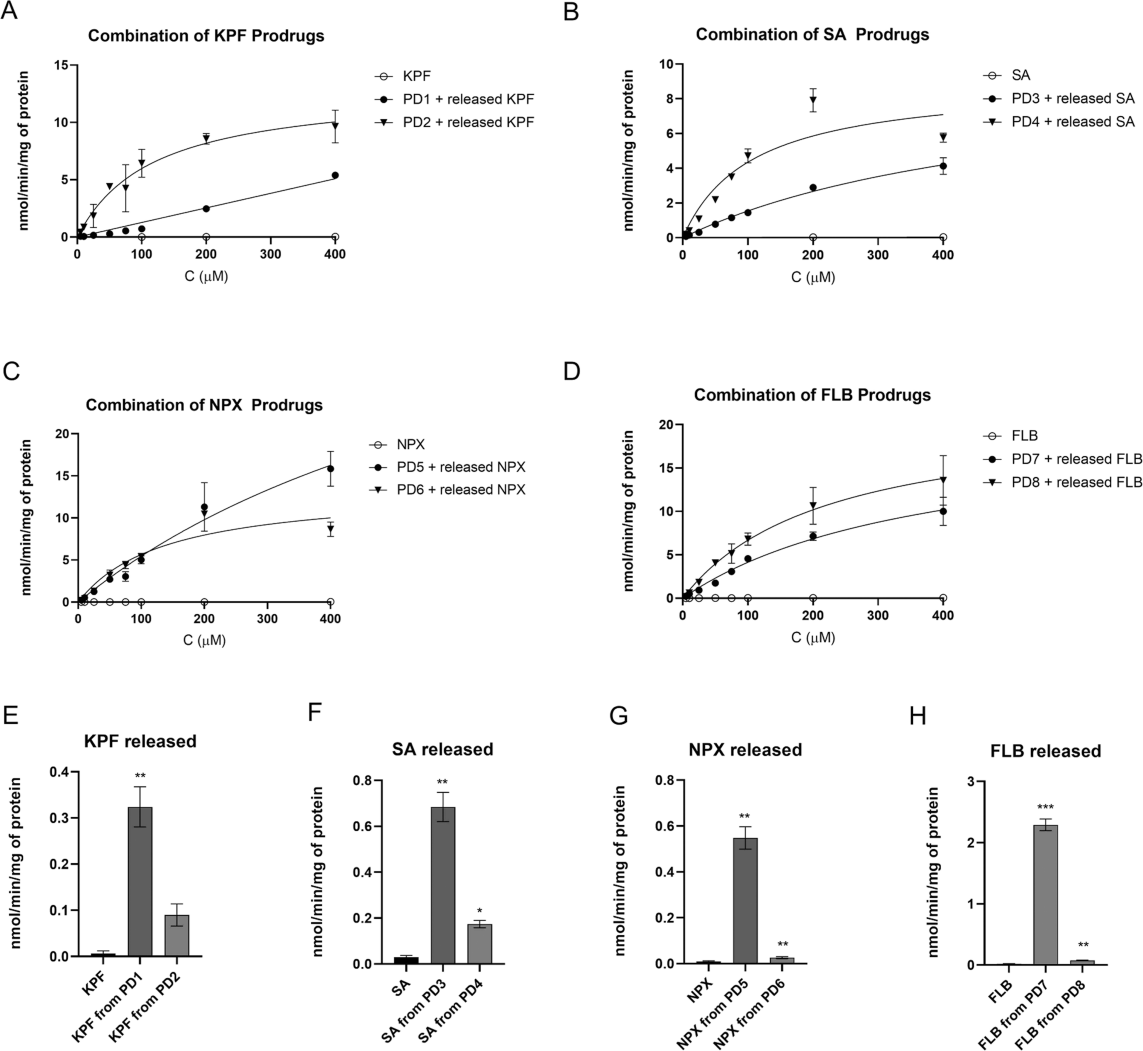
(A–D) Concentration-dependent cellular uptake of prodrugs **1**–**8** (5–400 μM; ● filled circles and ▼ down-facing triangles; including the proportion of the released parent drugs) compared to their parent drugs (○ hollow circles) in human glioma U-87MG cells. (E–H) Released parent drugs after the uptake of prodrugs **1**–**8** at 100 μM concentration into U-87MG cells compared to the uptake of the parent drug themselves. The data are presented as mean ± SD (*n* = 3–6).

Due to the selective and abundant expression of OATP1C1 in human U-87MG glioma cells (no other OATPs were detected), the cellular uptake of prodrugs **1**–**8** was studied first with these cells. Overall, all prepared prodrugs displayed higher cellular accumulation compared with their parent drugs (Figures 4 and Table 3). Moreover, some of these prodrugs displayed higher cellular accumulation into U-87MG cells compared to their corresponding LAT1-utilizing prodrugs (Supporting Information Figure S8), implying that OATP1C1-mediated cellular uptake of the corresponding prodrugs can be more effective than that of the LAT1-mediated uptake. Interestingly, all of the studied prodrugs released to some extent their parent drugs (up to 50% of the original prodrug amount; Figure 4E–H, S1). This hydrolysis was mainly enzymatic since all of the studied prodrugs were stable in the cell lysis buffer (0.1 M NaOH) and the amounts of released parent drugs were from hundreds to thousands of times greater than the uptake of the parent drug themselves in U-87MG cells (Figure 4E–H). Curiously, the cellular bioconversion was greater than that seen in the stability experiments (Table 1). This discrepancy may be explained by the fact that during the homogenization of the biological media, some of the enzyme activity can be lost. Alternatively, the bioconversion experiments may not contain all of the crucial supplements for each and every enzyme, while the culturing media for the cell experiments are richer for supplements and can better support diverse bioconversion reactions.

**Table 3:** Michaelis-Menten Kinetic Parameters (*K*_m_ and *V*_max_) for the Cellular Uptake of Prodrugs **1**–**8** and l-Thyroxine (T_4_) as a Positive Control into Human U-87MG Glioma Cells and Mouse Primary Astrocytes

	*K*_m_ (μM)		*V*_max_ (nmol/min/mg)	
prodrug	U-87MG	astrocytes	U-87MG	astrocytes
PD1	∼174.4^a^	n.d.^b^	∼1.27^a^	n.d.^b^
PD2	118 ± 27	123 ± 21	13.0 ± 1.3	5.5 ± 0.4
PD3	539 ± 97	685 ± 97	9.9 ± 1.2	13.0 ± 1.3
PD4	106 ± 32	86 ± 32	9.0 ± 1.1	5.5 ± 0.8
PD5	138 ± 79	n.d.^b^	9.0 ± 3.7	n.d.^b^
PD6	138 ± 36	n.d.^b^	13.5 ± 1.6	n.d.^b^
PD7	381 ± 73	360 ± 61	19.8 ± 2.3	10.3 ± 1.1
PD8	221 ± 48	58 ± 20	21.5 ± 2.4	3.0 ± 0.4
l-thyroxine, T	111 ± 32	1248 ± 230	8.86 ± 1.33	9.7 ± 6.1

a Due to the linear uptake, only estimations of exact *K*_m_ and *V*_max_ can be reported.

b Due to the linear uptake, no exact *K*_m_ and *V*_max_ can be reported.

**Figure 5:**
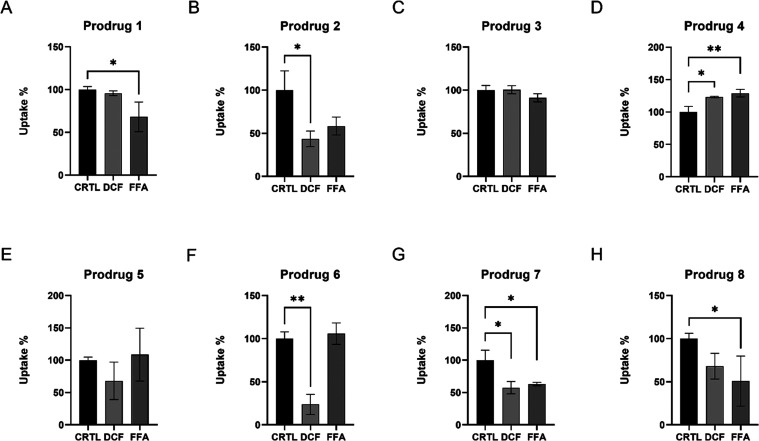
Cellular uptake (percentages (%) compared to control) of prodrugs **1**–**8** (100 μM) into the U-87MG human glioma cells in the presence of 100 μM OATP1C1 inhibitors diclofenac (DCF) and flufenamic acid (FFA). The data is presented as mean ± SD; *n* = 3 (*P < 0.05, **P < 0.01, one-way ANOVA, followed by Dunnett’s multiple comparison test).

Among ketoprofen prodrugs, prodrug **2** exhibited higher Michaelis–Menten parameters than prodrug **1** (*K*_m_ value of 118 vs 174 μM and *V*_max_ value of 13 vs 1.2 nmol/min/mg of protein), with a saturated plot against the concentration contrarily to prodrug **1** (Figure 4A, Table 3). Moreover, the uptake of prodrug **2** in the presence of the OATP1C1 inhibitors, DCF and FFA, was also reduced more effectively compared to prodrug **1**. Both of these observations suggest that prodrug **2** with a T_4_ promoiety is a more specific OATP1C1 substrate than prodrug **1** with a DIT promoiety (Figure 5A,B).

Also, salicylic acid-T_4_ prodrug **4** showed 5 times higher affinity than corresponding DIT prodrug **3** (*K*_m_ value of 106 vs 539 μM) and ca. 3.5 times higher transport capacity than prodrug **3** at 200 μM concentration (Figure 4B, Table 3). Curiously, we observed that in the presence of OATP1C1 inhibitors (DCF and FFA), the uptake of prodrug **4** was slightly increased, implying the use of other transportation systems (other than OATPs), whereas uptake of prodrug **3** was not affected significantly by the OATP1C1 inhibitors, indicating that it was not able to compete with DCF and/or FFA for OATP1C1-utilization (Figure 5C,D). Notably, the salicylic acid prodrugs were the smallest studied compounds in this series. When DCF and FFA were docked into the OATP1C1 model, it was evident that the docking poses and sites of these inhibitors and competing substrates were very similar to those of the studied prodrugs. MD analysis of these inhibitors showed similar interactions and binding modes as the substrates and prodrug designs (Supporting Information Figure S44). Therefore, the lack of inhibition seen in Figure 5 raises questions from the other facts discussed below.

Naproxen prodrugs **5** and **6** showed very similar uptake profiles to each other up to 200 μM concentration, with T_4_ prodrug **6** having a little higher *V*_max_ value than DIT prodrug **5** (13.5 vs 9.0 nmol/min/mg of protein) (Figure 4C). In addition, their cellular uptake was affected by DCF but not by FFA (Figure 5E,F), and the amount of reduction for prodrug **6** was greater (75%) compared to prodrug **5** (25%), denoting that T_4_ prodrug **6** is a more selective substrate for OATP1C1, similar to the ketoprofen prodrugs. This may arise from the fact that some of these prodrugs may be able to utilize some other amino acid transporters (other than OATP family members; the same phenomenon has been seen with LAT1-utilizing prodrugs in the past).^30,31^ For example, DCF can also interact with organic anion transporters (OAT) 1, 3, and 4, while FFA is known to interact only with OAT1.^32−35^ Therefore, if the studied prodrugs can utilize OAT3 or 4 in addition to OATP1C1, these inhibition studies can give confusing results. Therefore, cellular uptake studies should always be considered with caution.

Similarly to other prodrugs, flurbiprofen derivatives also had a slightly higher affinity and transport capacity with its T_4_ promoiety (prodrug **8**) than its DIT promoiety (prodrug **7**) (Figure 4D, Table 3). Furthermore, in the presence of OATP1C1 inhibitors (DCF or FFA), the uptake of both prodrugs was significantly reduced (Figure 5G,H). Thus, overall, it was concluded that the natural T_4_ promoiety yielded slightly better OATP substrates than non-natural DIT promoiety, although logically, the features of the parent drugs themselves affected the properties of the final prodrugs a lot, which was then further studied with molecular modeling.

### OATP1C1 Model Shows Hydrophobic and Polar Sites in the Predicted Binding Site

**Figure 6:**
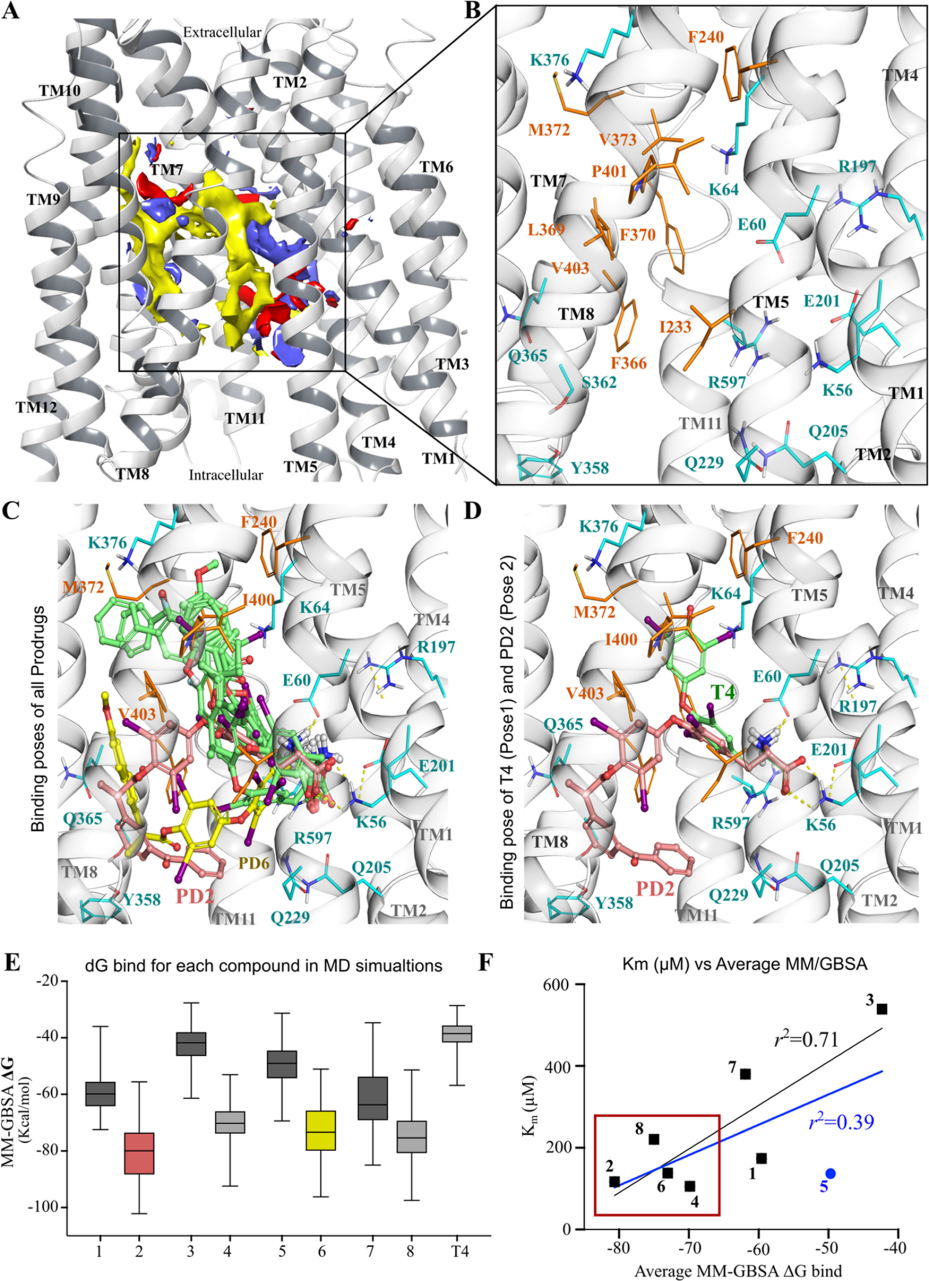
Structure of OATP1C1 was obtained from the AlphaFold database with ID AF-Q9NYB5-F1-model_v3. (A) SiteMap predicted site showing hydrophobic sites in yellow, hydrogen bond acceptors in red, and hydrogen bond donors in blue. (B) The binding site zoomed to show the residues, hydrophobic residues in orange, and polar residues in cyan. (C) Poses of all compounds (green) in the same orientation and prodrug **2** (light pink) and prodrug **6** (yellow) in different orientations. (D) Poses of T_4_ (green pose1) and prodrug **2** (light pink pose2) showing interactions with the polar residues Glu201 and Lys56. (E) Plot showing MM-GBSA dG bind values of each compound along 2.5 μs simulation data. (F) Plot showing the correlation between *K*_m_ (μM) and average MM-GBSA dG bind for all of the compounds and leaving out prodrug **5**, and showing the tetraiodo-based prodrugs in a red box with high dG bind energy.

To gain insights into the binding modes of the natural T_4_ and non-natural DIT promoiety, we employed the AlphaFold homology model of human OATP1C1 to conduct docking and evaluate the stability of the poses and interactions through MD simulations. Additionally, the MD data was utilized to calculate the free energies of binding using MM-GBSA. Moreover, we performed a principal component analysis on the comprehensive simulation data. The SiteMap analysis identified the best-ranked sites as a slightly slanted channel, primarily comprised of hydrophobic side chains, with certain polar residues directed toward the cytoplasmatic opening (Figure 6A,B and Supporting Information Figure S9). Hydrophobic contributions to the site originate from residues Ile233, Phe240, Phe366, Leu369, Phe370, Met372, Val373, Ile400, and Val403, while polar contributions come from residues Lys56, Glu60, Lys64, Arg197, Glu201, Gln205, Gln229, Lys376, and Arg597 (Figure 6B). All 8 designed prodrugs, as well as T_4_, as a reference substrate of OATP1C1, were docked into this pocket. The initial predicted T_4_ binding pose (Figure 6D) resembles the Pose2 type described in our previous modeling studies,^12^ where the amino acid portion (interactions with TM1 residues: Lys56 and Glu60) faces the intracellular cavity. For all of the prodrugs, the hydrophobic portion extends toward the extracellular region (Figure 6C), interacting with Phe240 (TM5), Phe366, Leu369 (TM7), Ile395, Ile400, and Val403 (TM8). All of the prodrugs and T_4_ (Figure 6C, green) have similar vertical binding poses, with the exception of prodrugs **2** and **6** (Figure 6C, salmon and yellow, respectively), which have shown a horizontal pose (Supporting Information Figure S10). These putative binding modes underwent MD simulations to assess their stability and interactions.

MD simulations revealed a range of dynamic behaviors, with some compounds displaying stable interactions, while others change the initial binding pose from horizontal to vertical. The prodrugs derived from DIT (**1**, **3**, **5**, and **7**) show similar dynamics, with some of them moving toward intracellular regions in certain replica runs, suggesting a common transport pathway. This is also supported by the lack of stable interactions with the transporter (Supporting Information, Figure S11). In contrast, prodrugs derived from tetraiodothyronine (T_4_ and compounds **2**, **4**, and **6**) demonstrate stable interactions throughout the simulations. T_4_ also shows stable interactions, but the pose varies from vertical to horizontal. This variability may be attributed to the presence of a negatively charged oxygen group on the phenyl ring, attempting to interact with the positively charged Lys376. The last T4 derivative **8**, transitioned from the initial vertical conformation to a horizontal pose and, in one replica, even moved toward the intracellular region after losing polar contacts. Nonetheless, most compounds exhibited stable polar interactions with Lys56, Glu60, Glu201, and Arg597. The amino group stabilized ionic interactions with either Glu60, Glu89, or Glu201 (Supporting Information Figure S11), supported by polar contacts with Gln205 and Gln229, while Lys56 and Arg597 interact with a carboxylate group (relevant for T_4_ and prodrug **2**). Notably, the positively charged amino acid Arg597 (or Arg601 in rat oatp1c1) is known to influence substrate binding and transport.^17^ Both Arg597 (TM11) and Lys56 (TM1) are positioned opposite to each other, creating a charged clamp for the carboxylate group and likely involved in the intracellular translocation. The MD simulation data was further used to calculate the binding free energies using the MM-GBSA method.

### MM-GBSA Differentiates the Binding between Diiodo and Tetraiodide-Based Prodrugs

The predicted binding free energies for all prodrugs were calculated along the trajectories of the MD simulations by using MM-GBSA (Figure 6E). As our compounds share a common core, we employed this method to check for correlations between the average binding free energies and experimental *K*_m_. The analysis of the total data set yields a correlation of 0.39 (Figure 6F, blue line). However, excluding prodrug **5**, which escapes the binding pocket in 3 out of 5 replicas, improved the correlation to 0.71 (Figure 6F, black line). Furthermore, the MM-GBSA average values (Figure 6E,F) distinguish between tetraiodothyronine derivates, which exhibited lower values than diiodo derivatives, depicting the relevance of the second diiodophenyl ring system and, consequently, the hydrophobic interactions. By utilizing MM-GBSA, we successfully differentiated between compounds with high and low *K*_m_ values with one outlier.

### Cellular Uptake of Novel Prodrugs into Mouse Primary Astrocytes is Mediated *via* oatp1a4/5/6

**Figure 7:**
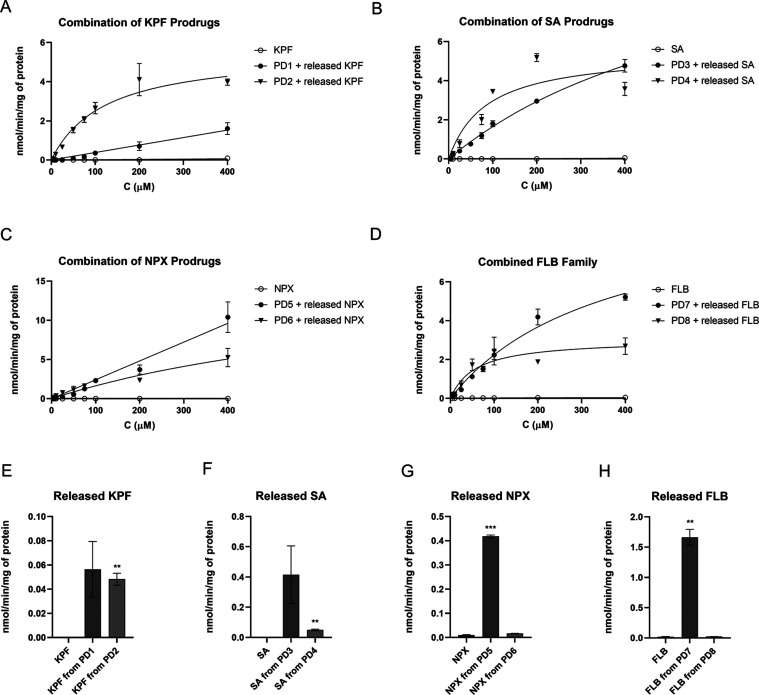
(A–D) Concentration-dependent cellular uptake of prodrugs **1**–**8** (5–400 μM; ● filled circles and ▼ down-facing triangles; including the proportion of released parent drugs) compared to their parent drugs (○ hollow circles) in mouse primary astrocytes. (E–H) Released parent drugs after the uptake of prodrugs **1**–**8** at 100 μM concentration into U-87MG cells compared to the uptake of the parent drug themselves. The data are presented as mean ± SD (*n* = 3–6).

To evaluate whether the prepared OATP1C1-utilizing prodrugs could be astrocyte-targeted, the cellular uptake of compounds **1**–**8** was also evaluated in the mouse primary astrocyte, despite their lack of oatp1c1 expression, but notable expression of oatp1a4/5/6. Unexpectedly, the prodrugs **1**–**8** were indeed uptaken into the astrocytes and to a greater extent than their corresponding parent drugs (Figures 7, S2, and Table 3). Despite the resemblance between the prodrug uptake curves in different cell types, the prodrug uptake in human U-87MG cells was mostly greater than that in mouse astrocytes. As with U-87MG cells, these prodrugs also released their parent drug in the mouse astrocytes, and similarly, this proportion of the released parent drug was significantly higher than the uptake of the parent drug themselves in astrocytes (Figure 7E–H).

**Figure 8:**
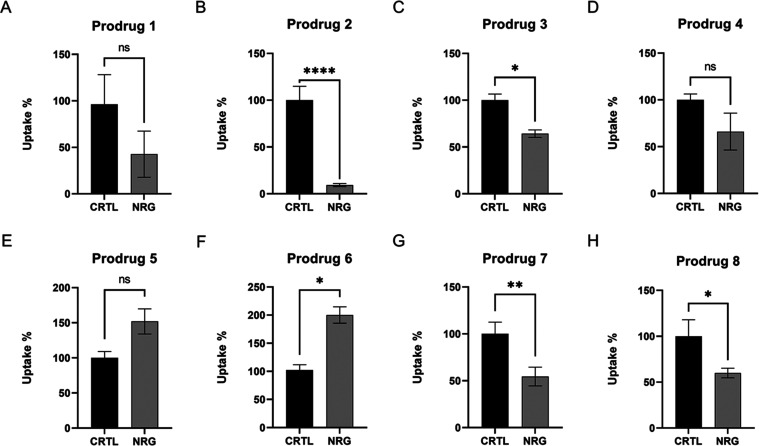
Cellular uptake (percentages (%) compared to control) of prodrugs **1**–**8** (100 μM) into the mouse primary astrocytes in the presence of 100 μM OATP1A2 inhibitor naringin (NRG). The data is presented as mean ± SD; *n* = 3 (*P < 0.05, **P < 0.01, one-way ANOVA, followed by Dunnett’s multiple comparison test).

With ketoprofen and salicylic acid prodrugs (**1**–**4**), the T_4_ derivatives were more effectively accumulated into the astrocytes than the corresponding DIT derivatives (Figure 7A,B, Table 3). Curiously, both naproxen prodrugs (**5** and **6**) displayed a linear uptake trend, and therefore, Michaelis–Menten parameters could not be calculated for their uptake (Figure 7C, Table 3). Unlike the other pairs of prodrugs, the uptake of the naproxen-T_4_ prodrug (**6**) was lower than that of its corresponding DIT derivative (**5**). Similarly, flurbiprofen-DIT derivative **7** displayed a higher capacity, whereas the T_4_ prodrug **8** displayed higher affinity (Figure 7D, Table 3). Moreover, most of the prodrugs seemed to have interactions with OATP1a4, 1a5, and/or 1a6 subtypes since their uptake was decreased in the presence of OATP1A2 inhibitor naringin (NRG, Figure 8A–H).^36^ Ketoprofen-T_4_ prodrug (**2**) seemed to have the greatest decrease in its uptake in the presence of this inhibitor, whereas naproxen prodrugs behaved oppositely; NRG-mediated oatp1A4/5/6 inhibition most likely drove these prodrugs (**5** and **6**) to use some other higher capacity transport mechanism, and therefore their uptake was increased in the presence of NRG. This can also explain the linear uptake behavior of these prodrugs, as seen in Figure 7.

### Potential Binding Mode of Mouse oatp1a2 Orthologs Supports Unspecific Binding

Since mouse astrocytes did not express oatp1c1 (Figure 2A), but rather orthologs of the human OATP1A2, namely, oatp1a4, oatp1a5, and oatp1a6 (sequence identities are presented in the Supporting Information, Figure S12), we also explored the potential interaction between our prodrugs and these mouse transporters using AlphaFold models and the same binding pocket as OATP1C1. Despite the conserved pocket, the docking poses with high scores in mouse transporters indicated differences in the orientation of prodrugs, while the poses in the human OATP1A2 are consistent with the OATP1C1 proposed binding mode of OATP1C1 (Supporting Information, Figure S13A,B). Interestingly, the docking poses in oatp1a4 differentiated between the diiodo-based prodrugs and tetraiodide-based prodrugs, with the first showing vertical poses, while the latter preferred horizontal pose (Supporting Information Figure S14A,B). Docking poses in oatp1a6 exhibited consistent orientations, with all of the compounds aligned in a similar pattern (Supporting Information, Figure S14E,F). Docking poses of mouse oatp1c1 (Supporting Information Figure S13C,D) show conserved interactions only with the highly conserved Arg600 (i.e., Arg601 in rat oatp1c1), which plays a role in the transport of substrates.^17^ These docking analyses support the idea of an unspecific binding in mice transporters. According to this model, our prodrugs would bind to OATP1A2 as well as the mouse orthologs’ 1a4, 1a5, and 1a6, which is consistent with the observed experimental uptake studies in mouse astrocytes.

### Brain Uptake of T_4_ Prodrug of Ketoprofen (PD2)

**Figure 9:**
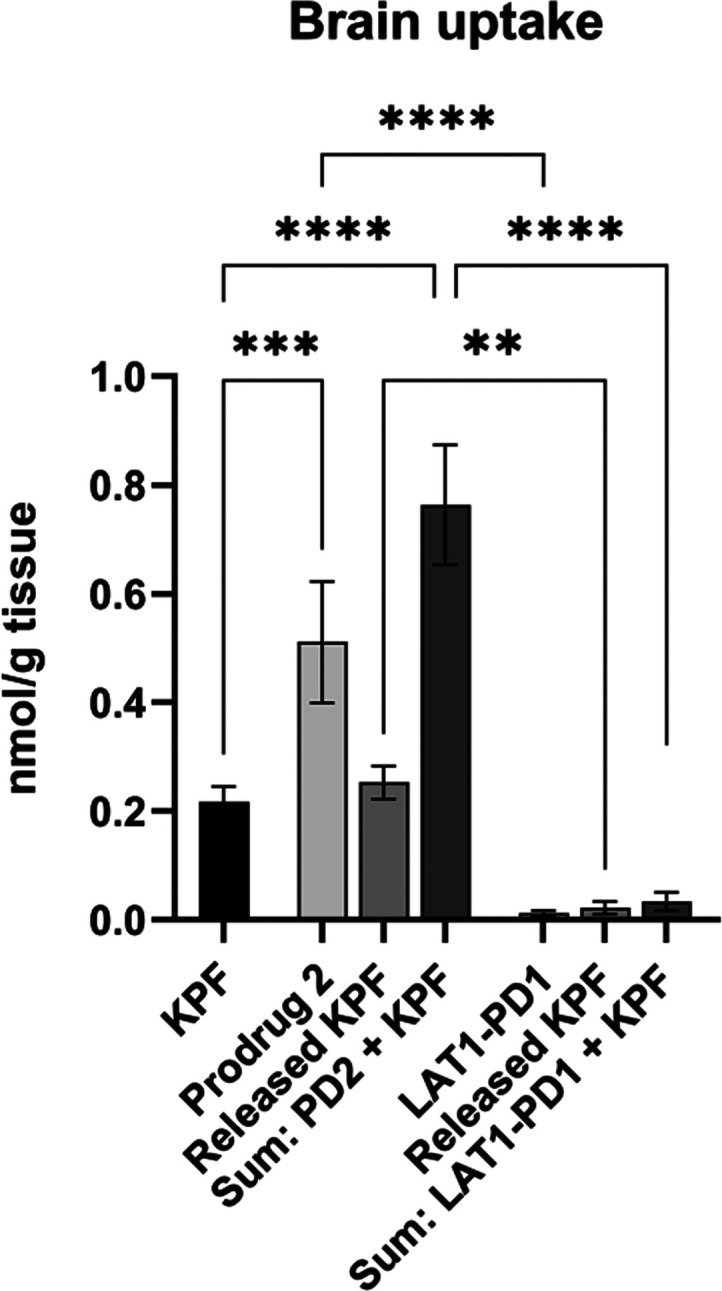
Brain uptake of prodrug **2**, ketoprofen, and LAT1-utilizing prodrug of ketoprofen (25 μmol/kg) after ip administration into mice analyzed from 30 min from injection. The results are presented as mean ± SD (*n* = 3–4), and an asterisk denotes a statistically significant difference (** *P* < 0.01, ***P < 0.001, ****P < 0.0001 one-way ANOVA followed by Tukey’s multiple comparison test).

Finally, the brain drug delivery of the prodrug candidate with the greatest potential for further development (appropriate OATP-mediated cellular uptake (the best affinity (118 μM), capacity (*V*_max_ 13.0 ± 1.3 nmol/min/mg protein), and selectivity (inhibition by DCF and FFA ca. 56 and 42%, respectively) in U-87MG cells) and bioconversion profile) was studied with mice. Ketoprofen-T_4_ prodrug **2** (25 μmol/kg) was administered intraperitoneally (i.p.) into mice and compared to our previous results with similar amounts of ketoprofen or a LAT1-utilizing prodrug (LAT1-PD-KPF1).^19^ This LAT1-utilizing prodrug of ketoprofen was the same one used for the comparison of the cellular uptake of prodrugs **1** and **2** into U-87MG cells in Figure S8 and has proven to have the greatest LAT1-mediated brain accumulation and ability to deliver ketoprofen to the brain from all LAT1-utilizing prodrugs that we have studied in the past. As seen in Figure 9, after 30 min from the injection of the compounds, the accumulation of prodrug **2** (prodrug and released ketoprofen; 0.78 ± 0.13 nmol/g of tissue) was significantly greater than the amounts of ketoprofen (0.22 ± 0.03 nmol/g of tissue) or LAT1-utilizing prodrug (prodrug and released ketoprofen; 0.03 ± 0.02 nmol/g of tissue) detected in the brain. Although ketoprofen released from prodrug **2** in the brain was at the same level as ketoprofen administration (0.24 ± 0.03 nmol/g of tissue), it was 10 times greater than the released ketoprofen from LAT1-utilizing prodrug (0.022 ± 0.011 nmol/g of tissue), implying that this prodrug has a great potential to deliver greater amount ketoprofen to the brain. Furthermore, it is highly possible that prodrug **2** functions as a reservoir and releases ketoprofen slowly in the brain, giving greater AUC_brain_ of ketoprofen than the administration of the parent drug itself, and therefore, the *t*_max_ points among ketoprofen and ketoprofen released from prodrug **2** are most likely different from each other. Thus, this needs to be studied in more detail in the future. Noteworthily, this study proves that prodrugs with larger size (Mw. 1013 g/mol in this case) can be delivered into the brain via a transporter-mediated route.

## Conclusions

In conclusion, this study successfully designed and synthesized eight novel OATP1C1-utilizing prodrugs. The chemical stability and the enzymatic bioconversion of these prodrugs having ester prodrug bonds were slower than expected, and curiously, the prodrugs having T_4_ promoiety (**2**, **4**, **6**, and **8**) displayed higher accumulation into human U-87MG glioma cells highly expressing OATP1C1 than the prodrugs with DIT promoiety (**1**, **3**, **5**, and **7**). The proposed binding mode of these prodrugs to OATP1C1 suggests two initial conformations where the conformations change from vertical to horizontal positions during simulations. The calculated binding energies of these prodrugs showed higher binding energy for the T_4_ derivatives **2**, **4**, **6**, and **8** when compared to the DIT promoiety prodrugs, which is in line with their transport profile. Novel prodrugs were also noticed to utilize oatp1a4/1a5/1a6 in mouse primary astrocytes, which did not express oatp1c1 on their plasma membrane. Docking of the prodrugs into the human OATP1A2 and mouse orthologs oatp1a4/1a5/1a6 revealed binding modes similar to OATP1C1, which supports their binding to these transporters. Moreover, the improved brain drug delivery in mice (*in vivo*) was proven with a single-point analysis of the most promising prodrug candidate for further development. Given that human ortholog to the three transporters, OATP1A2, is expressed at the human BBB and OATP1C1 in human astrocytes, the proposed prodrug approach has a great potential to be utilized for improving the drug delivery into the human brain and astrocytes, although possible secondary and tertiary transport mechanisms beyond the OATP family needs to be carefully considered in the future.

## Experimental Section

### General Synthesis

All reactions were performed with commercial reagents obtained from Sigma-Aldrich (St. Louis, MO), Acros Organics (Waltham, MA) or Merck (Darmstadt, Germany), Thermo Fisher Scientific (Heysham, China), Ambeed (Arlington Hts, IL), and AK Scientific (Union City, CA). All of the solvents used in the reactions were anhydrous. Dichloromethane (DCM), *N,N-*dimethylformamide (DMF), and tetrahydrofuran (THF) were dried over molecular sieves (4 Å) and were stored under an inert atmosphere. All reactions were performed under an inert atmosphere of argon or nitrogen unless otherwise specified. Selected reactions were performed in Microwave synthesizers (Biotage Initiator^+^) using either 10- or 20 mL reaction glass vials at an appropriate temperature. Reactions were monitored by thin-layer chromatography using aluminum sheets coated with silica gel 60 F245 (0.24 mm) with a suitable visualization agent. Purifications by flash chromatography (BUCHI, Sepacore flash systems X10) were performed on silica gel 60 (0.063–0.200 mm mesh) cartridges. The ^1^H and ^13^C NMR spectra were recorded on a Bruker Avance HD III 600 spectrometer, equipped with a 5 mm cryogenically cooled BBO probe head and operating at 600.18 and 150.93 MHz, respectively. All NMR experiments were measured at 298 K. Chemical shifts are reported in ppm on the δ scale from an internal standard of solvent (DMSO-*d*_6_ referenced to 2.50 (^1^H), 39.52 (^13^C), and MeOD-*d*_4_ referenced to 3.31 (^1^H), 49.00 (^13^C) ppm). The spectra were processed from the recorded FID files with Mestrenova software. The following abbreviations are used: s, singlet; br. s, broad singlet; d, doublet; dd, doublet of doublets; ddd, doublet of doublets of doublets; t, triplet; td, triplet of doublets; q, quartet; p, pentet; and m, multiplet. Coupling constants are reported in Hz. Electrospray ionization mass spectrometry (ESI-MS) spectra were recorded with an Agilent 1260 Infinity LC system coupled with an Agilent 6410 triple quadrupole mass spectrometer with an electrospray ionization source (Agilent Technologies, Palo Alto, CA). Over 95% of the purities of the final compounds were confirmed by high-performance liquid chromatography (HPLC) analysis (see more details of instrumentation below). The main traces of impurities (0.61–4.77%) in these final products were their unreacted or liberated parent drugs during purification and storage. Since the parent drugs were not substrates for OATPs as such, it was considered that these traces do not affect the final conclusions of cellular uptake studies of these prodrugs mediated *via* OATPs. The HPLC chromatograms (Figures S15–S18, S23–S25, S30–S32, S37–S39), ^1^H NMR (Figures S19, S21, S26, S28, S33, S35, S40, S42), and ^13^C-NMRs (Figures S20, S22, S27, S29, S34, S36, S41, S43) of all final prodrugs **1**–**8** are provided in the Supporting Information.

### Synthesis of the Prodrugs

#### (*S*)-4′-(4-Hydroxy-3,5-diiodobenzyl)-9λ^4^-boraspiro[bicyclo[3.3.1]nonane-9,2′-[1,3,2]oxazaborolidin]-5′-one (**10**)

Under an argon atmosphere, DIT (**9**, 3.0 g, 6.93 mmol, 1 equiv) was suspended in THF (50 mL) and vigorously stirred for 1 h. To this, a solution of 9-borabicyclo[3.3.1]nonane (9-BBN) (0.5 M solution in THF, 22 mL, 70.2 mmol, 22 equiv) was added dropwise over 20 min, and the resulting suspension was stirred for 3 days further at room temperature. After completion of the reaction, the reaction mixture was concentrated *in vacuo* to afford the title compound **10**. The resulting crude product (3.75 g, 6.78 mmol, 97%) was used in the next step with no further purification. **TLC** (*R*_*f*_) = 0.90 (DCM/MeOH, 9:1). ^**1**^**H NMR** (600 MHz, DMSO*-d*_6_): δ 7.72 (s, 2H), 3.82 (br. s, 1H), 3.06 (d, *J* = 14.4 Hz, 1H), 2.78–2.71 (m, 1H), 1.65–1.20 (m, 14H). ^**13**^**C NMR** (151 MHz, DMSO-*d*_6_): δ 172.91, 153.94, 139.84 (2C), 133.45, 86.85 (2C), 55.67, 33.91, 31.31, 31.23, 30.74, 30.56, 26.10, 24.24, 23.91, 21.77.

#### (*S*)-4′-(4-(4-Hydroxy-3,5-diiodophenoxy)-3,5-diiodobenzyl)-9λ^4^-boraspiro[bicyclo[3.3.1]nonane-9,2′-[1,3,2]oxazaborolidin]-5′-one (**16**)

Under an argon atmosphere, T_4_ (**15**, 3.0 g, 3.86 mmol, 1 equiv) was suspended in THF (50 mL) and vigorously stirred for 1 h. To this, a solution of 9-BBN (0.5 M solution in THF, 12 mL, 42.5 mmol, 22 equiv) was added dropwise over 20 min, and the resulting suspension was stirred for 3 days further at room temperature. After completion of the reaction, the reaction mixture was concentrated *in vacuo* to afford the title compound **10**. The resulting crude product (3.41 g, 3.79 mmol, 98%) was used in the next step with no further purification. **TLC** (*R*_*f*_) = 0.85 (DCM/MeOH, 9:1). ^**1**^**H NMR** (600 MHz, DMSO-*d*_6_): δ 7.95 (s, 2H), 7.11 (s, 2H), 3.96 (br. s, 1H), 3.17 (d, *J* = 13.4 Hz, 1H), 2.91 (dd, *J* = 14.3, 9.2 Hz, 1H), 1.62–1.20 (m, 14H). ^**13**^**C NMR** (151 MHz, DMSO-*d*_6_): δ 172.85, 151.41, 150.94, 150.14, 140.88 (2C), 139.21, 125.05 (2C), 91.71 (2), 87.80 (2), 55.37, 34.19, 31.86, 31.27, 31.20, 30.65, 26.08, 24.21, 23.89, 21.75.

### General Procedure for Coupling Reactions of **10** and **16**

**Method A**: To the parent drug (ketoprofen, salicylic acid, naproxen, or flurbiprofen, 2 equiv), 1-ethyl-3-(3-dimethylaminopropyl) carbodiimide hydrochloride (EDCI·HCl; 2 equiv) and 4-dimethylaminopyridine (DMAP) (2 equiv) were added at room temperature, and the solids were dissolved in a mixture of DCM/DMF (10:1, 20 mL). Under an argon atmosphere, the reaction mixture was refluxed for 2 h. Upon cooling to room temperature, a solution of either **10** (1 equiv) or **16** (1 equiv) in dry DMF (1 mL) was added dropwise over 20 min. The resulting reaction mixture was heated to 65 °C for 3 days. Upon completion of the reaction, H_2_O (50 mL) was added, and the aqueous phase was extracted with DCM (3 × 50 mL). The combined organic layers were washed with H_2_O (50 mL), followed by brine (50 mL), dried over Na_2_SO_4_, and concentrated *in vacuo*. The crude residue was purified by flash chromatography using DCM/MeOH (0–100%) + 0.5% (v/v) of Et_3_N as an eluent.

**Method B**: Into a microwave reactor glass reaction 20 mL vial, the parent drug (ketoprofen, salicylic acid, naproxen, or flurbiprofen, 2 equiv), EDCI·HCl (2 equiv), and DMAP (2 equiv) were added at room temperature and the solids were dissolved in a mixture of DCM/DMF (10:1, 20 mL). Under an argon atmosphere, the reaction mixture was stirred at room temperature for 30 min, followed by the dropwise addition of a solution of either **10** (1 equiv) or **16** (1 equiv) in dry DMF (1 mL) over 20 min. The resulting reaction mixture was irradiated in a microwave reactor (100 °C) for 30 min. Upon completion of the reaction, H_2_O (50 mL) was added, and the aqueous phase was extracted with DCM (3 × 50 mL). The combined organic layers were washed with H_2_O (50 mL), followed by brine (50 mL), dried over Na_2_SO_4_, and concentrated *in vacuo*. The crude residue was purified by flash chromatography using DCM/MeOH (0–100%) + 0.5% (v/v) of Et_3_N as an eluent.

#### 2,6-Diiodo-4-(((*S*)-5′-oxo-9λ^4^-boraspiro[bicyclo[3.3.1]nonane-9,2′-[1,3,2]oxazaborolidin]-4′-yl)methyl)phenyl 2-(3-benzoylphenyl)propanoate λ^4^(**11**)

Following method B, starting material **10** (100.0 mg, 0.181 mmol) was subjected to the coupling reaction. Purification was done using CombiFlash chromatography (gradient elution, DCM/MeOH (0–100%) + 0.5% (v/v) of Et_3_N, 45 min). The retention time of the product was 13 min. The product (**11**) was obtained as a white amorphous solid (100 mg, 0,13 mmol, 79%). **TLC** (*R*_*f*_) = 0.89 (DCM/MeOH, 9:1). ^**1**^**H NMR** (600 MHz, DMSO-*d*_6_): δ 7.87 (d, *J* = 7.2 Hz, 2H), 7.84–7.75 (m, 3H), 7.74–7.63 (m, 3H), 7.63–7.50 (m, 4H), 4.34 (q, *J* = 6.9 Hz, 1H), 3.92 (br. s, 1H), 3.15 (d, *J* = 15.3 Hz, 1H), 2.81 (dd, *J* = 14.7, 9.1 Hz, 1H), 1.69 (d, *J* = 7.1 Hz, 3H), 1.66–1.10 (m, 14H). ^**13**^**C NMR** (151 MHz, DMSO-*d*_6_): δ 195.60, 172.80, 170.40, 149.33, 140.08, 139.51, 139.35 (2C), 137.15, 136.97, 132.76, 132.53, 129.68 (2C), 129.38, 128.90, 128.83, 128.59 (2C), 91.18, 90.70, 54.90, 44.58, 34.26, 31.84, 31.26, 30.70, 30.56, 26.07, 24.19, 23.87, 21.74, 18.46.

#### (*S*)-2,6-Diiodo-4-((5′-oxo-9λ^4^-boraspiro[bicyclo[3.3.1]nonane-9,2′-[1,3,2]oxazaborolidin]-4′-yl)methyl)phenyl 2-hydroxybenzoate (**12**)

Following method B, starting material **10** (500.0 mg, 0.9 mmol) was subjected to the coupling reaction. Purification was done using CombiFlash chromatography (gradient elution, DCM/MeOH (0–100%) + 0.5% (v/v) of Et_3_N, 45 min). The retention time of the product was 9 min. The product (**12**) was obtained as a white, amorphous solid (318 mg, 0.47 mmol, 52%). **TLC** (*R*_*f*_) = 0.79 (DCM/MeOH, 9:1). ^**1**^**H NMR** (600 MHz, DMSO-*d*_6_): δ 8.09 (d, *J* = 7.9 Hz, 1H), 7.95 (s, 2H), 7.62 (d, *J* = 8.7 Hz, 1H), 7.09 (d, *J* = 8.4 Hz, 1H), 7.07 (t, *J* = 7.9 Hz, 1H), 4.26–4.22 (m, 1H), 3.96 (td, *J* = 13.4, 9.0 Hz, 1H), 3.20 (dd, *J* = 14.7, 4.4 Hz, 1H), 1.56–1.36 (m, 14H). ^**13**^**C NMR** (151 MHz, DMSO-*d*_6_): δ 172.84, 163.88, 160.37, 149.51, 139.63 (2C), 136.47, 133.63, 131.23, 119.63, 117.86, 112.76, 91.64 (2C), 55.29, 34.36, 31.85, 31.30, 30.74, 30.58, 26.08, 24.22, 23.90, 21.75.

#### 2,6-Diiodo-4-(((*S*)-5′-oxo-9λ^4^-boraspiro[bicyclo[3.3.1]nonane-9,2′-[1,3,2]oxazaborolidin]-4′-yl)methyl)phenyl (*S*)-2-(6-methoxynaphthalen-2-yl)propanoate (**13**)

Following method A, starting material **10** (250.0 mg, 0.45 mmol) was subjected to the coupling reaction. Purification was done using CombiFlash chromatography (gradient elution, DCM/MeOH (0–100%) + 0.5% (v/v) of Et_3_N, 40 min). The retention time of the product was 13 min. The product (**13**) was obtained as a white, amorphous solid (143 mg, 0.19 mmol, 41%). **TLC** (*R*_*f*_) = 0.89 (DCM/MeOH, 9:1). ^**1**^**H NMR** (600 MHz, DMSO-*d*_6_): δ 7.90–7.82 (m, 4H), 7.62 (s, 1H), 7.60 (dd, *J* = 8.5, 1.6 Hz, 1H), 7.32 (d, *J* = 2.4 Hz, 1H), 7.18 (dd, *J* = 8.9, 2.5 Hz, 1H), 4.31 (q, *J* = 7.2 Hz, 1H), 3.88 (s, 3H), 3.87 (s, 1H), 3.18–3.14 (m, 1H), 2.83 (dd, *J* = 10.6, 4.2 Hz, 1H), 1.63–1.34 (m, 14H), 1.45 (d, *J* = 6.9 Hz, 3H). ^**13**^**C NMR** (151 MHz, DMSO-*d*_6_): δ 175.45, 170.84, 157.29, 149.52, 140.06 (2C), 139.42, 134.03, 133.55, 129.31, 128.35, 126.97, 126.90, 126.57, 118.75, 105.77, 91.29, 90.88, 55.18, 54.90, 44.86, 34.27, 31.84, 31.21, 30.76, 30.57, 26.08, 24.19, 23.87, 21.74, 18.65.

#### 2,6-Diiodo-4-(((*S*)-5′-oxo-9λ^4^-boraspiro[bicyclo[3.3.1]nonane-9,2′-[1,3,2]oxazaborolidin]-4′-yl)methyl)phenyl 2-(2-fluoro-[1,1′-biphenyl]-4-yl)propanoate (**14**)

Following method A, starting material **10** (250 mg, 0.45 mmol) was subjected to the coupling reaction. Purification was done using CombiFlash chromatography (gradient elution, DCM/MeOH (0–100%) + 0.5% (v/v) of Et_3_N, 40 min). The retention time of the product was 7 min. The product (**14**) was obtained as a white, amorphous solid (249 mg, 0.32 mmol, 71%). **TLC** (*R*_*f*_) = 0.91 (DCM/MeOH, 9:1). ^**1**^**H NMR** (600 MHz, DMSO-*d*_6_): δ 7.59–7.56 (m, 3H), 7.42 (m, 5H), 7.23 (m, 2H), 4.30 (q, *J* = 6.9 Hz, 1H), 3.93 (br. s, 1H), 3.17 (br. s, 1H), 2.83 (d, *J* = 10.5 Hz, 1H), 1.79–1.42 (m, 14H), 1.41 (d, *J* = 6.5 Hz, 3H). ^**13**^**C NMR** (151 MHz, DMSO-*d*_6_): δ 172.81, 170.26, 159.69 (C–F), 158.06 (C–F), 149.38, 143.10, 140.08 (2C), 134.89, 134.82, 130.68, 128.78 (2C), 128.60 (2C), 127.77, 126.58, 124.85, 115.85, 90.87, 90.87, 55.22, 44.08, 34.28, 31.84, 31.27, 30.76, 30.58, 26.08, 24.20, 23.88, 21.74, 18.26.

#### 4-(2,6-Diiodo-4-(((*S*)-5′-oxo-9λ^4^-boraspiro[bicyclo[3.3.1]nonane-9,2′-[1,3,2]oxazaborolidin]-4′-yl)methyl)phenoxy)-2,6-diiodophenyl (*S*)-2-(3-benzoylphenyl)propanoate (**17**)

Following method A, the starting material **16** (300,0 mg, 0.33 mmol) was subjected to the coupling reaction. Purification was done using CombiFlash chromatography (gradient elution, DCM/MeOH (0–100%) + 0.5% (v/v) of Et_3_N, 40 min). The retention time of the product was 15 min. The product (**17**) was obtained as a white, amorphous solid (597 mg, 0.53 mmol, 94%). **TLC** (*R*_*f*_) = 0.86 (DCM/MeOH, 9:1). ^**1**^**H NMR** (600 MHz, DMSO-*d*_6_): δ 7.96 (s, 2H), 7.74–7.66 (m, 5H), 7.62 (s, 2H), 7.57 (d, *J* = 11.8 Hz, 4H), 4.25 (s, 1H), 4.10 (s, 1H), 3.18 (s, 2H), 1.53 (d, *J* = 8.4 Hz, 3H), 1.42–1.22 (m, 14H). ^**13**^**C NMR** (151 MHz, DMSO-*d*_6_): δ 195.65, 173.44, 170.69, 153.94, 150.93, 150.18, 141.17, 140.85, 139.27 (2C), 137.16, 136.96, 132.75, 132.50, 129.69 (2C), 129.56, 129.36, 128.84, 128.57 (2C), 125.06 (2C), 91.49 (2C), 87.70 (2C), 56.05, 44.54, 35.77, 31.84, 30.76, 26.67, 26.08, 25.10, 24.33, 21.74, 18.43.

#### (*S*)-4-(2,6-Diiodo-4-((5′-oxo-9λ^4^-boraspiro[bicyclo[3.3.1]nonane-9,2′-[1,3,2]oxazaborolidin]-4′-yl)methyl)phenoxy)-2,6-diiodophenyl 2-hydroxybenzoate (**18**)

Following method A, starting material **16** (250.0 mg, 0.28 mmol) was subjected to the coupling reaction. Purification was done using CombiFlash chromatography (gradient elution, DCM/MeOH (0–100%) + 0.5% (v/v) of Et_3_N, 45 min). The retention time of the product was 12 min. The product (**18**) was obtained as a white, amorphous solid (131 mg, 0.13 mmol, 46%). **TLC** (*R*_*f*_) = 0.70 (DCM/MeOH, 9:1). ^**1**^**H NMR** (600 MHz, DMSO-*d*_6_): δ 7.94 (s, 2H), 7.85 (s, 1H), 7.62 (t, *J* = 7.0 Hz, 1H), 7.37–7.17 (m, 2H), 7.14–7.05 (m, 2H), 3.62 (s, 1H), 3.17 (m, 2H), 1.62–1.30 (m, 14H). ^**13**^**C NMR** (151 MHz, DMSO-*d*_6_): δ 172.79, 164.11, 160.41, 155.28, 154.06, 140.97 (2C), 139.52, 136.52, 131.21, 129.97, 125.05 (2C), 119.62, 117.90, 112.62, 92.25, 91.69, 91.56, 87.76, 55.36, 36.00, 31.84, 29.01, 28.07, 26.67, 26.08, 24.33, 23.87, 21.74.

#### 4-(2,6-Diiodo-4-(((*S*)-5′-oxo-9λ^4^-boraspiro[bicyclo[3.3.1]nonane-9,2′-[1,3,2]oxazaborolidin]-4′-yl)methyl)phenoxy)-2,6-diiodophenyl (*S*)-2-(6-methoxynaphthalen-2-yl)propanoate (**19**)

Following method A, starting material **16** (250.0 mg, 0.28 mmol) was subjected to the coupling reaction. Purification was done using CombiFlash chromatography (gradient elution, DCM/MeOH (0–100%) + 0.5% (v/v) of Et_3_N, 12 min). The retention time of the product was 40 min. The product (**19**) was obtained as a white, amorphous solid (180 mg, 0.16 mmol, 65%). **TLC** (*R*_*f*_) = 0.81 (DCM/MeOH, 9:1). ^**1**^**H NMR** (600 MHz, DMSO-*d*_6_): δ 7.88–7.69 (m, 5H), 7.41 (dd, *J* = 8.5, 1.6 Hz, 1H), 7.32 (d, *J* = 5.1 Hz, 1H), 7.29 (d, *J* = 2.3 Hz, 1H) 7.16 (d, *J* = 2.6 Hz, 1H), 7.15 (d, *J* = 2.4 Hz, 1H), 3.87 (s, 3H), 3.80 (q, *J* = 7.1 Hz, 1H), 2.90 (d, *J* = 8.4 Hz, 2H), 2.74 (s, 1H), 1.45 (d, *J* = 7.1 Hz, 3H) 1.65–1.28 (m, 14H). ^**13**^**C NMR** (151 MHz, DMSO-*d*_6_): δ 171.10, 162.30, 157.10, 153.85, 150.99, 146.27, 140.94 (2C), 139.51, 136.31, 133.23, 129.10, 128.39, 126.92, 126.83, 126.40, 125.55 (2C), 118.68, 105.69, 92.50, 91.69, 91.49 (2C), 55.33, 55.15, 44.57, 34.21, 31.85, 30.76, 30.64, 26.67, 26.08, 25.11, 24.33, 21.75, 18.43.

#### 4-(2,6-Diiodo-4-(((*S*)-5′-oxo-9λ^4^-boraspiro[bicyclo[3.3.1]nonane-9,2′-[1,3,2]oxazaborolidin]-4′-yl)methyl)phenoxy)-2,6-diiodophenyl 2-(2-fluoro-[1,1′-biphenyl]-4-yl)propanoate (**20**)

Following method A, starting material **16** (200.0 mg, 0.22 mmol) was subjected to the coupling reaction. Purification was done using CombiFlash chromatography (gradient elution, DCM/MeOH (0–100%) + 0.5% (v/v) of Et_3_N, 45 min). The retention time of the product was 15 min. The product (**20**) was obtained as a white, amorphous solid (160 mg, 0.14 mmol, 72%). **TLC** (*R*_*f*_) = 0.83 (DCM/MeOH, 9:1). ^**1**^**H NMR** (600 MHz, DMSO-*d*_6_): δ 7.96 (s, 2H), 7.58–7.54 (m, 3H), 7.49–7.46 (m, 3H), 7.43 (s, 1H), 7.43–7.41 (m, 1H), 7.27–7.19 (m, 2H), 4.31–4.27 (m, 1H), 3.19 (dd, *J* = 14.7, 4.0 Hz, 1H), 3.00 (br. s, 1H), 2.89 (d, *J* = 7.5 Hz, 1H), 1.70 (d, *J* = 7.2 Hz, 3H), 1.45–1.20 (m, 14H). ^**13**^**C NMR** (151 MHz, DMSO-*d*_6_): δ 172.78, 170.53, 159.69 (C–F), 158.05 (C–F), 153.91, 150.99, 146.14, 140.95 (2C), 140.56, 139.51, 134.82, 130.74, 128.80 (2C), 128.59 (2C), 127.86, 127.16, 125.24, 124.84 (2C), 115.98, 91.89, 91.49 (2C), 91.26, f55.33, 44.19, 34.24, 31.84, 31.24, 31.18, 30.65, 26.67, 25.10, 24.33, 21.74, 18.44.

### Deprotection of the Protected Prodrugs (**PD1**–**8**)

**Method C:** To the 9-BBN-protected coupling product (**17**), a mixture of CHCl_3_/MeOH (10:1, 10 mL) was added at room temperature. To this solution, *tert*-butyl hydroperoxide (TBHP, 5.5 M in nonane, 1 mL, 2 equiv) was added at room temperature, and the reaction mixture was stirred in open air without septum for 4 days. Upon completion, the reaction mixture was concentrated *in vacuo*. The crude residue was purified by flash chromatography (MeOH/DCM + 0.5% triethylamine) to afford the final prodrug (**2**).

**Method D:** To the 9-BBN-protected coupling product (**11**, **12**, **13**, **14**, **18**, **19**, or **20**) in a microwave reactor glass vial was added a mixture of MeOH/DCM (5:1, 10 mL) at room temperature. To this solution, aq. HCl (1 M, 2 equiv) was added, and the reaction mixture was irradiated in a microwave reactor at 100 °C for 30–60 min (TLC control). Upon completion, the reaction mixture was concentrated in a vacuum. The crude residue was purified by flash chromatography (MeOH/DCM + 0.5% triethylamine) to afford the final prodrug (**1**, **3**, **4**, **5**, **6**, **7**, or **8**).

#### (2*S*)-2-Amino-3-(4-((2-(3-benzoylphenyl)propanoyl)oxy)-3,5-diiodophenyl)propanoic acid (**1**)

The coupling reaction followed method D on a 130 mg, 0.165 mmol scale. Purification was done using CombiFlash chromatography (gradient elution, DCM/MeOH (0–100%) + 0.5% (v/v) of Et_3_N, 45 min). The retention time of the product was 23 min. The product (**1**) was obtained as a white, amorphous solid (68 mg, 0.1 mmol, 62%). **TLC** (*R*_*f*_) = 0.40 (DCM/MeOH, 1:1). ^**1**^**H NMR** (600 MHz, MeOD): δ 7.85–7.77 (m, 6H), 7.72 (dt, *J* = 7.8, 1.4 Hz, 1H), 7.65 (td, *J* = 7.4, 1.3 Hz, 1H), 7.57 (t, *J* = 7.7 Hz, 1H), 7.52 (t, *J* = 7.7 Hz, 2H), 4.26 (q, *J* = 7.2 Hz, 1H), 4.12 (s, 1H), 3.24 (s, 1H), 3.10–2.98 (m, 1H), 1.75 (d, *J* = 7.2 Hz, 3H). ^**13**^**C NMR** (151 MHz, MeOD): δ 198.35, 172.25, 152.52, 141.85, 140.88, 139.16, 138.75, 133.94, 131.17, 131.11, 130.34, 129.94, 129.59, 91.28, 49.57, 46.88, 35.37, 18.69. **MS** (ESI – positive mode): For C_25_H_22_I_2_NO_5_ [M + H] calculated: 669.96; found: 669.90. **MS** (ESI – negative mode): For C_25_H_20_I_2_NO_5_ [M – H] calculated: 667.94; found: 668.00. HPLC purity 97.80%.

#### (*S*)-2-Amino-3-(4-((2-hydroxybenzoyl)oxy)-3,5-diiodophenyl)propanoic acid (**3**)

The coupling reaction followed method D on a 200.0 mg, 0.3 mmol scale. Purification was done using CombiFlash chromatography (gradient elution, DCM/MeOH (0–100%) + 0.5% (v/v) of Et_3_N, 35 min). The retention time of the product was 16 min. The product (**3**) was obtained as a white, amorphous solid (119 mg, 0.21 mmol, 66%). **TLC** (*R*_*f*_) = 0.35 (DCM/MeOH, 1:1). ^**1**^**H NMR** (600 MHz, MeOD): δ 8.18–8.12 (m, 1H), 7.90 (d, *J* = 9.1 Hz, 2H), 7.66–7.60 (m, 1H), 7.05 (dddt, *J* = 12.1, 7.1, 5.0, 2.4 Hz, 2H), 4.01 (d, *J* = 42.3 Hz, 1H), 3.29 (s, 1H), 3.16–3.05 (m, 1H). ^**13**^**C NMR** (151 MHz, MeOD): δ 167.93, 163.31, 152.13, 141.84, 138.24, 136.81, 132.00, 120.92, 118.84, 112.82, 91.61, 91.57, 49.57, 36.27. **MS** (ESI – positive mode): For C_16_H_14_I_2_NO_5_ [M + H] calculated: 553.89; found: 553.70. **MS** (ESI – negative mode): For C_16_H_12_I_2_NO_5_ [M – H] calculated: 551.88; found: 551.90. HPLC purity 96.01%.

#### (*S*)-2-Amino-3-(3,5-diiodo-4-(((*S*)-2-(6-methoxynaphthalen-2-yl)propanoyl)oxy)phenyl)propanoic acid (**5**)

The coupling reaction followed method D on a 143 mg, 0.19 mmol scale. Purification was done using CombiFlash chromatography (gradient elution, DCM/MeOH (0–100%) + 0.5% (v/v) of Et_3_N, 45 min). The retention time of the product was 26 min. The product (**5**) was obtained as a white, amorphous solid (58 mg, 0.1 mmol, 48%). **TLC** (*R*_*f*_) = 0.30 (DCM/MeOH, 1:1). ^**1**^**H NMR** (600 MHz, DMSO): δ 7.92–7.75 (m, 5H), 7.58 (d, *J* = 8.3 Hz, 1H), 7.31 (s, 1H), 7.16 (d, *J* = 8.8 Hz, 1H), 4.28 (d, *J* = 7.0 Hz, 1H), 3.86 (s, 3H), 3.16 (s, 1H), 3.08 (d, *J* = 13.4 Hz, 1H), 2.92 (s, 1H), 1.73 (d, *J* = 6.8 Hz, 3H). ^**13**^**C NMR** (151 MHz, DMSO): δ 170.88, 157.30, 149.53, 140.22, 139.59, 134.05, 133.57, 129.34, 128.36, 127.00, 126.92, 126.59, 118.76, 105.80, 91.55, 91.07, 55.21, 54.82, 44.89, 34.83, 18.64. **MS** (ESI – positive mode): For C_23_H_22_I_2_NO_6_ [M + H] calculated: 645.96; found: 645.80. **MS** (ESI – negative mode): For C_23_H_20_I_2_NO_6_ [M – H] calculated: 643.94; found: 644.10. HPLC purity 96.66%.

#### (2*S*)-2-Amino-3-(4-((2-(2-fluoro-[1,1′-biphenyl]-4-yl)propanoyl)oxy)-3,5-diiodophenyl)propanoic acid (**7**)

The coupling reaction followed method D on a 229.1 mg, 0.29 mmol scale. Purification was done using CombiFlash chromatography (gradient elution, DCM/MeOH (0–100%) + 0.5% (v/v) of Et_3_N, 37 min). The retention time of the product was 21 min. The product (**7**) was obtained as a white, amorphous solid (103 mg, 0.16 mmol, 54%). **TLC** (*R*_*f*_) = 0.41 (DCM/MeOH, 1:1). ^**1**^**H NMR** (600 MHz, DMSO + 10% MeOD): δ 7.81–7.73 (m, 2H), 7.59–7.52 (m, 3H), 7.50–7.45 (m, 2H), 7.44–7.38 (m, 3H), 4.28 (q, *J* = 7.2 Hz, 1H), 3.49 (q, *J* = 6.3 Hz, 1H), 3.06 (dd, *J* = 14.5, 4.7 Hz, 1H), 2.88 (dd, *J* = 14.4, 7.3 Hz, 1H), 1.70 (d, *J* = 7.2 Hz, 3H). ^**13**^**C NMR** (151 MHz, DMSO + 10% MeOD): δ 170.33, 158.96 (d, ^1^*J*_C–F_ = 246.0 Hz), 149.53, 140.73 (d, ^3^*J*_C–F_ = 8.0 Hz), 140.30, 139.53, 134.91, 130.81 (d, ^4^*J*_C–F_ = 3.6 Hz), 128.85 (d, ^4^*J*_C–F_ = 2.9 Hz), 128.66, 127.92, 127.30 (d, ^3^*J*_C–F_ = 13.0 Hz), 124.91 (d, ^4^*J*_C–F_ = 3.1 Hz), 115.99 (d, ^2^*J*_C–F_ = 23.4 Hz), 91.39, 91.05, 54.69, 44.34, 34.86, 29.81, 18.48. **MS** (ESI – positive mode): For C_24_H_21_FI_2_NO_4_ [M + H] calculated: 659.95; found: 659.90. **MS** (ESI – negative mode): For C_24_H_19_FI_2_NO_4_ [M – H] calculated: 657.94; found: 657.60. HPLC purity 95.38%.

#### (2*S*)-2-Amino-3-(4-(4-((2-(3-benzoylphenyl)propanoyl)oxy)-3,5-diiodophenoxy)-3,5-diiodophenyl)propanoic acid (**2**)

The coupling reaction followed method C on a 250.0 mg, 0.22 mmol scale. Purification was done using CombiFlash chromatography (gradient elution, DCM/MeOH (0–100%) + 0.5% (v/v) of Et_3_N, 42 min). The retention time of the product was 26 min. The product (**2**) was obtained as a white, amorphous solid (74 mg, 0.07 mmol, 33%). **TLC** (*R*_*f*_) = 0.30 (DCM/MeOH, 1:1). ^**1**^**H NMR** (600 MHz, MeOD): δ 7.93–7.89 (m, 3H), 7.82–7.76 (m, 3H), 7.74–7.70 (m, 1H), 7.64 (tdd, *J* = 7.3, 4.6, 2.8 Hz, 1H), 7.56 (t, *J* = 7.7 Hz, 1H), 7.54–7.49 (m, 2H), 7.20 (d, *J* = 27.7 Hz, 2H), 4.30–4.21 (m, 2H), 3.30 (s, 1H), 3.12 (dd, *J* = 14.5, 7.5 Hz, 1H), 1.74 (d, *J* = 7.2 Hz, 3H). ^**13**^**C NMR** (151 MHz, MeOD): δ 198.47, 172.20, 155.37, 154.26, 148.25, 142.68, 140.94, 139.11, 138.72, 138.06, 133.97, 133.94, 131.23, 131.12, 130.27, 129.94, 129.59, 127.36, 91.90, 90.93, 90.40, 49.57, 46.83, 35.66, 18.69. **MS** (ESI – positive mode): For C_31_H_24_I_4_NO_6_ [M + H] calculated: 1013.78; found: 1013.60. **MS** (ESI – negative mode): For C_31_H_22_I_4_NO_6_ [M – H] calculated: 1011.76; found: 1011.80. HPLC purity 98.10%.

#### (*S*)-2-Amino-3-(4-(4-((2-hydroxybenzoyl)oxy)-3,5-diiodophenoxy)-3,5-diiodophenyl)propanoic acid (**4**)

The coupling reaction followed method D on a 103 mg, 0.1 mmol scale. Purification was done using CombiFlash chromatography (gradient elution, DCM/MeOH (0–100%) + 0.5% (v/v) of Et_3_N, 36 min). The retention time of the product was 23 min. The product (**4**) was obtained as a white, amorphous solid (57 mg, 0.06 mmol, 63%). **TLC** (*R*_*f*_) = 0.31 (DCM/MeOH, 1:1). ^**1**^**H NMR** (600 MHz, DMSO): δ 8.08 (d, *J* = 7.7 Hz, 1H), 7.85 (s, 2H), 7.60 (t, *J* = 7.6 Hz, 1H), 7.30 (s, 1H), 7.12 (d, *J* = 8.5 Hz, 2H), 7.04 (t, *J* = 7.3 Hz, 1H), 3.52 (d, *J* = 12.5 Hz, 2H), 2.88 (s, 1H). ^**13**^**C NMR** (151 MHz, DMSO): δ 169.27, 163.97, 160.46, 154.15, 151.40, 151.02, 140.99, 139.63, 136.37, 131.25, 125.03, 119.47, 117.94, 112.76, 92.47, 91.91, 91.81, 87.93, 54.92, 34.93. **MS** (ESI – positive mode): For C_22_H_16_I_4_NO_6_ [M + H] calculated: 897.72; found: 897.70. **MS** (ESI – negative mode): For C_22_H_14_I_4_NO_6_ [M – H] calculated: 895.70; found: 895.40. HPLC purity 97.44%.

#### (*S*)-2-Amino-3-(4-(3,5-diiodo-4-(((*S*)-2-(6-methoxynaphthalen-2-yl)propanoyl)oxy)phenoxy)-3,5-diiodophenyl)propanoic acid (**6**)

The coupling reaction followed method D on a 180 mg, 0.16 mmol scale. Purification was done using CombiFlash chromatography (gradient elution, DCM/MeOH (0–100%) + 0.5% (v/v) of Et_3_N, 40 min). The retention time of the product was 20 min. The product (**6**) was obtained as a white, amorphous solid (94 mg, 0.1 mmol, 59%). **TLC** (*R*_*f*_) = 0.35 (DCM/MeOH, 1:1). ^**1**^**H NMR** (600 MHz, DMSO): δ 7.91 (s, 1H), 7.88–7.81 (m, 4H), 7.58 (d, *J* = 8.4 Hz, 1H), 7.31 (d, *J* = 2.7 Hz, 1H), 7.26 (s, 1H), 7.22–7.11 (m, 2H), 4.27 (q, *J* = 7.1 Hz, 1H), 3.86 (d, *J* = 1.8 Hz, 3H), 3.54 (t, *J* = 6.5 Hz, 1H), 3.23–3.14 (m, 1H), 2.89 (dd, *J* = 14.4, 8.4 Hz, 1H), 1.72 (dd, *J* = 7.3, 1.9 Hz, 3H). ^**13**^**C NMR** (151 MHz, DMSO): δ 171.11, 157.29, 153.95, 150.99, 146.20, 140.99, 139.64, 134.00, 133.56, 129.34, 128.36, 126.95, 126.93, 126.56, 125.15, 118.75, 105.79, 92.21, 91.97, 91.78, 91.65, 55.20, 54.95, 44.85, 34.89, 18.67. **MS** (ESI – positive mode): For C_29_H_24_I_4_NO_6_ [M + H] calculated: 989.77; found: 989.60. **MS** (ESI – negative mode): For C_29_H_22_I_4_NO_6_ [M – H] calculated: 987.76; found: 987.80. HPLC purity 95.89%.

#### (2*S*)-2-Amino-3-(4-(4-((2-(2-fluoro-[1,1′-biphenyl]-4-yl)propanoyl)oxy)-3,5-diiodophenoxy)-3,5-diiodophenyl)propanoic acid (**8**)

The coupling reaction followed method D on a 95 mg, 0.08 mmol scale. Purification was done using CombiFlash chromatography (gradient elution, DCM/MeOH (0–100%) + 0.5% (v/v) of Et_3_N, 30 min). The retention time of the product was 24 min. The product (**8**) was obtained as a white, amorphous solid (95 mg, 0.08 mmol, 99%). **TLC** (*R*_*f*_) = 0.30 (DCM/MeOH, 1:1). ^**1**^**H NMR** (600 MHz, DMSO): δ 7.85 (s, 2H), 7.59–7.52 (m, 3H), 7.48 (dd, *J* = 8.5, 6.9 Hz, 2H), 7.44–7.38 (m, 3H), 7.25 (d, *J* = 27.0 Hz, 2H), 4.28 (q, *J* = 7.1 Hz, 1H), 3.48–3.46 (m, 4H), 3.16 (td, *J* = 14.6, 10.4 Hz, 1H), 2.87–2.78 (m, 1H), 1.69 (d, *J* = 7.2 Hz, 3H). ^**13**^**C NMR** (151 MHz, DMSO): δ 170.54, 158.88 (d, ^1^*J*_C–F_ = 246.0 Hz), 154.02, 151.19 (d, ^2^*J*_C–F_ = 68.2 Hz), 146.07, 140.92 (d, ^3^*J*_C–F_ = 9.7 Hz), 140.63 (d, ^3^*J*_C–F_ = 8.3 Hz), 134.83, 130.77 (d, ^4^*J*_C–F_ = 3.9 Hz), 128.80 (d, ^4^*J*_C–F_ = 3.2 Hz), 128.71 (d, ^4^*J*_C–F_ = 2.8 Hz), 128.62, 128.53 (d, ^3^*J*_C–F_ = 13.0 Hz), 127.88, 127.21 (d, ^3^*J*_C–F_ = 13.2 Hz), 125.24, 125.03, 124.85 (d, ^4^*J*_C–F_ = 3.0 Hz), 115.92 (d, ^2^*J*_C–F_ = 23.6 Hz), 91.90 (d, ^3^*J*_C–F_ = 11.5 Hz), 91.62 (d, ^3^*J*_C–F_ = 18.3 Hz), 87.86, 55.02, 44.22, 35.11, 18.47. **MS** (ESI – positive mode): For C_30_H_23_FI_4_NO_5_ [M + H] calculated: 1003.77; found: 1003.80. **MS** (ESI – negative mode): For C_30_H_21_FI_4_NO_5_ [M – H] calculated: 1001.76; found: 1001.50. HPLC purity 95.18%.

### General—Bioanalytics

All reagents and solvents used in analytical studies were commercial and high purity analytical grade or ultra-gradient HPLC-grade purchased from Sigma (St. Louis, MO), J.T. Baker (Deventer, The Netherlands), Merck (Darmstadt, Germany), or Riedel-de Han (Seelze, Germany). Water was purified using a Milli-Q Gradient system (Millipore, Milford, MA).

Pooled human liver S9 fraction (>20 mg of protein/mL) was purchased from Sigma–Aldrich (St. Louis, MO) and pooled sterile and filtered mouse serum from Biowest (Nuaillé, France). The pooled human plasma was obtained from the Finnish Red Cross (healthy human donors) under an applied license for research purposes. All biological materials were stored at −80 °C until used.

U-87MG (ATCC, HTB-14, unknown origin) was purchased from Lonza (Basel, Switzerland). The cells were cultured in standard conditions (37 °C, 5% CO_2_) using Dulbecco’s modified Eagle’s medium (DMEM) supplemented with l-glutamine (2.0 mM), heat-inactivated fetal bovine serum (FBS) (10%), penicillin (50 U/mL), and streptomycin (50 μg/mL). U-87MG cells (passages 8–25) were seeded at a density of 1 × 10^5^ cells/well onto 24-well plates and used for the uptake experiments 1 day after seeding. All of the studies were carried out as three biological replicates from the same cell passage.

The mouse primary astrocytes were isolated from neonatal pups (P0-2) of wild-type mice (C57BL/6JOlaHsd) as previously described, in compliance with the European Commission Directive 2010/63/EU and approved by the Institutional Animal Care and Use Committee of the University of Eastern Finland (Animal Usage Plan numbers: EKS-006-2019).^37^ Neonatal pups were decapitated, and their brains were collected in an ice-cold HBSS buffer. After tissue dissociation and washing, cells were plated on poly-l-lysine-coated flasks and cultured in DMEM supplemented with FBS, l-glutamine, and penicillin/streptomycin (50 μg/mL) for 24 h. Debris and dead cells were removed by DPBS rinsing. After 7 days, microglial cells were removed, and the remaining astrocytic monolayer was collected by trypsinization using 0.05% trypsin-ethylenediaminetetraacetic acid (EDTA) (Gibco), pelleted, and stored at −80 °C for subsequent analysis. For the cell uptake experiments, the astrocytes were seeded on 24-well plates with a density of 10^4^ cells/well 3 days before the experiments.

### Relative Expression of Transporters in Primary Astrocytes

The primary mouse astrocytes were lysed, reduced, and carboxymethylated prior to the digestion with TPCK-trypsin, as described previously by Montaser et al.^38^ Briefly, primary mouse astrocytes were solubilized in sodium dodecyl sulfate buffer (4% SDS, 100 mM Tris-HCl, pH 7.6). Then, the protein samples were processed, and the buffer was exchanged by following filter-aided sample preparation (FASP) as previously described previously.^39,40^ The data was acquired using a nanoElute system (Bruker Daltonics, Bremen, Germany) connected to a timsTOF Pro mass spectrometer (Bruker Daltonics, Bremen, Germany) following data-independent acquisition mode. The data was processed by DIA-NN software (version 1.8) using the library-free DIA analysis mode and the normalized MaxLFQ values, which were used for relative quantification.^41−43^

### Quantitative Expression of Transporters in U-87MG Cells

The protein expression levels of OATP1C1, along with LAT1 and a housekeeping protein Na^+^/K^+^-ATPase, were quantified from the plasma membrane fractions of human glioma U-87MG cells by using multiplexed multiple reaction monitoring (MRM) analysis according to the protocol described by Uchida et al.^44^ First, the crude membrane fractions were isolated from three distinct sets of cell culture plates using the Membrane Protein Extraction Kit (BioVision Incorporated, Milpitas, CA) according to the manufacturer’s instructions. The protein content for each fraction was measured by the Bio-Rad Protein Assay, based on the Bradford dye-binding method (EnVision, PerkinElmer, Inc., Waltham, MA). A total amount of 50 μg of protein from each fraction was solubilized/denatured in 7 M guanidine hydrochloride, 0.5 M Tris-HCl, and 10 mM EDTA-Na. The proteins were then reduced by dithiothreitol (1:50, w/w) and *S*-carboxymethylated by iodoacetamide (1:20, w/w). The alkylated proteins were precipitated by methanol/chloroform/water (4:1:3) and centrifuged at 18 000*g* for 5 min at 4 °C. The pellet was resuspended in 6 M urea and mixed for 10 min at room temperature before the dilution with 0.1 M Tris-HCl (pH 8.5) to a final concentration of 1.2 M urea and dissolved completely by intermittent sonication (Branson 3510, Danbury, CT). The dissolved proteins were first digested with LysC (1/100, w/w) and 0.05% ProteaseMax (Promega Biotech AB, Nacka, Sweden) for 3 h at room temperature. Then, the samples were spiked with 10 μL (30 fmol) of the heavy-labeled peptides for absolute quantification (JPT Peptide Technologies GmbH, Berlin, Germany; Table 4). The samples were incubated with (1/100, w/w) TPCK-Trypsin (Promega Biotech AB, Nacka, Sweden) for 18 h at 37 °C. The tryptic digestion was then quenched by adding 40 μL of 5% formic acid. The samples were then centrifuged at 18 000*g* for 5 min at 4 °C, and the supernatants were transferred to vials for analysis.

**Table 4:** SRM/MRM Transitions for Absolute Quantitative Proteomics

					MRM transitions(*m*/*z*)	
protein	St/IS	unique amino acid sequence	retention time (min)	transition number	precursor ion (Q1)	product ions (Q3)
OATP1C1	St	LYDSNVFR	22.1	1	507.3	900.4
				2		737.4
				3		622.3
	IS	LYDSNVF*R	22.1	1	512.3	910.5
				2		747.4
				3		632.4
LAT1	St	VQDAFAAAK	13.7	1	460.7	821.4
				2		578.3
				3		507.3
	IS	VQDAFAAAK*	13.7	1	464.8	829.4
				2		586.3
				3		515.3
Na^+^/K^+^-ATPase	St	AAVPDAVGK	10.4	1	414.2	685.4
				2		586.3
				3		489.3
	IS	AAVPDAV*GK	10.4	1	417.2	691.4
				2		592.3
				3		495.3

St—standard, IS—internal standard. The bold letter with* denotes labeled arginine (R) or lysine (K) with a stable isotope ^13^C and ^15^N.

The digested peptides were analyzed using a UPLC system (1290, Agilent Technologies, Santa Clara, CA) coupled with a triple quadrupole mass spectrometer with a heated electrospray ionization source in positive mode (MSD 6495, Agilent Technologies, Santa Clara, CA). A total amount of 20 μL of the digested peptides (10 μg) was separated using an AdvanceBio Peptide Map 2.1 mm × 250 mm, 2.7 μm column (Agilent Technologies, Santa Clara, CA) and LC eluents of 0.1% formic acid in water (A) and acetonitrile (B). The peptides were eluted following a constant flow rate of 0.3 mL/min and a gradient of 2–7% B for 2 min, followed by 7–30% B for 48 min, 30–45% B for 3 min, and 45–80% B for 2.5 min before re-equilibrating the column again for 4.5 min. OATP1C1, LAT1, and a housekeeping protein Na^+^/K^+^-ATPase were quantified based on the ratio between the light and heavy standard peptides, as described previously (Table 4).^23,45^ Data were acquired using Agilent MassHunter Workstation Acquisition (Agilent Technologies, Data Acquisition for Triple Quadrupole, version B.03.01) and processed by using Skyline software (version 20.1). The results were expressed as fmol/μg of the total amount of protein in the samples.

### Functionality of OATPs in Cells

After removal of the culture medium, U-87MG human glioma cells and mouse primary astrocytes were carefully washed with prewarmed Hank’s balanced salt solution (HBSS) containing 125.0 mM NaCl (or choline chloride in Na^+^ free conditions), 4.8 mM KCl, 1.2 mM MgSO_4_, 1.2 mM KH_2_PO_4_, 1.3 mM CaCl_2_, 5.6 mM glucose, and 25.0 mM 4-(2-hydroxyethyl)piperazine-1-ethanesulfonic acid (HEPES) with the pH adjusted to either 7.4 or pH 8.5 with 1 M NaOH (or KOH in sodium-free conditions). In the experiments at lower pH (4.5–6.5), 25.0 mM HEPES was replaced by 2-(*N*-morpholino) ethanesulfonic acid (MES), and the pH was adjusted to 4.5, 5.5, and 6.5 by 1.0 M NaOH. Preincubation was done with 500 μL of prewarmed HBSS at 37 °C for 10 min before adding the substrate for the uptake experiments. The functionality of OATP1C1 in human glioma U-87MG cells was studied with a known substrate (l-thyroxine, T_4_) and of oatp1a4, 1a5, and 1a6 in mouse primary astrocytes with [6,7-^3^H(*N*)]-estrone-3-sulfate (ES) by incubating the uptake solution (250 μL in HBSS) consisting of 5–400 μM of the substrate at 37 °C for 30 min. After incubation, the uptake was stopped by adding 500 μL of ice-cold HBSS, and the cells were washed two times with ice-cold HBSS (2 × 500 μL). The cells were then lysed with 500 μL of 0.1 M NaOH (60 min), the lysate was neutralized, and the protein was precipitated with acidic acetonitrile (ACN with 4.8% formic acid). The supernatants were analyzed by the high-performance liquid chromatography (HPLC) methods described below (T_4_) or by using a liquid scintillation counter (MicroBeta2 counter, PerkinElmer, Waltham, MA) (ES). The concentrations of T_4_ and ES were calculated from the spiked standard curve and normalized with the protein concentrations. The protein concentrations on each plate were determined as a mean of three samples by Bio-Rad Protein Assay, based on the Bradford dye-binding method, using bovine serum albumin (BSA) as a standard protein and measuring the absorbance (595 nm) by a multiplate reader (EnVision, PerkinElmer, Inc., Waltham, MA). The OATP-mediated uptake was confirmed by changing the pH of the uptake buffer in the absence of sodium ions, and in the case of OATP1C1 in human U-87MG cells, the uptake of T_4_ (100 μM) was studied in the presence of competing OATP1C1-ligands, diclofenac and flufenamic acid (100 μM), and in the case of oatp1a4/1a5/1a6 in mouse primary astrocytes, the uptake of ES (100 μM) was studied in the presence of competing OATO1A2-ligand, naringin (100 μM).

### Chemical Stability of Prodrugs **1**–**8**

The rates of chemical pH-dependent hydrolysis of prodrugs **1**–**8** were determined at 37 °C in 0.1 M NaOH and 50 mM Tris–HCl buffer at pH 7.4. The incubation mixtures were prepared by dissolving 10 mM prodrug **1**–**8** in DMSO in preheated buffer solutions. The DMSO concentration in the incubation mixtures was 2%, and the prodrug concentration in the beginning was 100 μM. The mixtures were incubated for 24 h (Tris–HCl buffer) and 1 h (NaOH), then the samples were withdrawn at appropriate intervals. ACN was added to the samples (1:1, v/v) to hinder further hydrolysis during the HPLC analyses. After the HPLC analysis (described below), the pseudo-first-order half-lives (*t*_1/2_) for the hydrolysis of the prodrug were calculated from the slope of the linear portion of the plotted logarithm of the remaining prodrug versus time.

### Enzymatic Bioconversion of Prodrugs **1**–**8**

The rates of bioactivation of the prodrugs **1**–**8** in mouse and human liver S9 fractions, as well as in mouse primary astrocyte–microglia homogenate, were determined at +37 °C. The incubation mixtures were prepared by mixing liver S9 subcellular fraction or brain cell homogenate (final protein concentration 1.0 mg/mL) with isotonic Tris–HCl buffer (pH 7.4) and 10 mM prodrug stock solution in DMSO (the initial concentration of prodrugs was 100 μM and the DMSO concentration was 2%). The mixture was incubated for 5 h, and the samples (100 mL) were withdrawn at appropriate intervals. The enzymatic reaction was terminated by the addition of ice-cold acetonitrile (100 mL), and the samples were centrifuged for 5 min at 12 000 rpm at room temperature and kept on ice until the supernatants were analyzed by the HPLC method described below. In blank reactions, the S9 fractions were replaced with the same volume of buffer. The pseudo-first-order half-lives (*t*_1/2_) for the rates of bioconversion of the prodrugs were calculated from the slope of the linear portion of the plotted logarithm of the remaining prodrug concentration versus time. The rates of bioactivation of prodrugs **1**–**8** in human plasma and mouse serum were determined at 37 °C as above by adding 10 mM stock solution of the prodrug to plasma/serum in a ratio of 1:10 and analyzed similarly to the samples from S9 fractions.

### Transporter-Mediated Uptake of Compounds into Cells

Cellular uptake of prodrugs **1**–**8** was studied as described for l-thyroxine above by adding compounds at a concentration of 5–400 μM in prewarmed HBSS buffer (250 μL) on the cell layer and incubating at 37 °C for 30 min (uptake was linear with all compounds up to 30 min). Subsequently, the cells were washed three times with ice-cold HBSS and lysed with 500 μL of 0.1 M NaOH (60 min). The lysate was neutralized; the proteins were precipitated with acidic ACN (containing 4.8% formic acid), and the supernatants were analyzed by the (HPLC) methods described below. The concentrations of each prodrug normalized to protein concentration were calculated from the standard curve prepared by spiking known concentrations of compounds into ACN-precipitated cell lysate.

The competitive uptake in the presence of OATP-inhibitors or competing substrates (100 μM), diclofenac (DFC) and flufenamic acid (FFA), for OATP1C1 and naringin (NRG) for OATP1A2 was carried out as described above with HBSS buffer at pH 7.4 containing 100 μM of the studied compound. The cells were preincubated with the inhibitors for 10 min, and the incubation mixture was removed before adding the studied compound and the inhibitor to the cells. The competitive uptake (30 min) with the inhibitor was then carried out as the normal uptake described above. The concentrations of the studied compounds were analyzed by the HPLC methods described below, calculated from the spiked standard curve, and normalized with the protein concentrations.

### High-Performance Liquid Chromatography (HPLC) Analyses

The amount of prodrug **1**–**8** was determined by the HPLC system, which consisted of an Agilent 1100 binary pump (Agilent Technologies Inc., Wilmington, DE), an 1100 micro vacuum degasser, an HP 1050 Autosampler, and an HP 1050 variable wavelength detector, operated at 230–240 nm. The chromatographic separations were achieved on an Agilent Zorbax SB-C18 analytical column (4.4 mm × 150 mm, 5 μm) (Agilent Technologies Inc., Wilmington, DE) by using isocratic elution of water containing 0.1% formic acid (pH ca. 3.0) and acetonitrile containing 0.1% formic acid with ratios of 50:50, 30:70, 40:60, 50:50, 50:50, 40:60, 40:60, and 30:70 (v/v), respectively, for prodrug **1**–**8**. Retention time for prodrug 1 was ca. 3.07 min and for ketoprofen 4.77 min, prodrug 2 was ca. 3.39 min and for ketoprofen 2.35 min, prodrug 3 was ca. 3.01 min and for salicylic acid 3.92 min, prodrug 4 was ca. 6.69 min and for salicylic acid 2.92 min, prodrug 5 was ca. 3.42 min and for naproxen 4.95 min, prodrug 6 was ca. 6.42 min and for naproxen 3.15 min, prodrug 7 was ca. 2.96 min and for flurbiprofen 4.87 min, and prodrug 8 was ca. 5.02 min and for ketoprofen 2.94 min at the flow rate of 1.0 mL/min at room temperature. The lower limit of quantification for prodrugs **1**–**8** was 0.20 μM. These HPLC methods were also accurate, precise, and selective over the range of 0.5–20 μM.

### Brain Drug Delivery (*In Vivo*)

Seven-week-old healthy male mice (JAXC57BL/6J) weighing 25 ± 4 g were supplied by the Laboratory Animal Center of the University of Eastern Finland (Kuopio, Finland). Animals were housed in well-ventilated stainless-steel cages with ad-libitum consumption of tap water and food pellets (Teklad Global Diet 2016, Inotiv, Netherlands) on a 12 h light and 12 h dark cycle with a room temperature of 22 ± 2 °C and relative humidity of 50–60%. The procedures were conducted under a license (ESAVI-2020-025070) approved by the Finnish Project Authorization Board and in accordance with the European Community Guidelines (Directive 2010/63/EU) and “Principles of Laboratory Animal Care” (NIH publication #85-23, revised in 1985). All efforts were made to minimize the number of animals used and their suffering.

A stock solution of 80 mM prodrug **2** was prepared in DMSO 1 day before the study. The stock solution was diluted with saline containing 30% (w/v) of Macrogol (15)-hydroxystearate (BASF SE, Germany) into a 2.5 mM formulation with the final DMSO concentration of 3%. Mice were dosed with the drug via intraperitoneal (ip) bolus administration (25 μmol/kg). To remove excess blood from the brain vasculature, the mice were anesthetized using pentobarbital (120 mg/kg i.p.) (Euthoxin Vet, Chanelle Pharmaceuticals Manufacturing Ltd., Ireland) approximately 5 min before a transcardial perfusion with ice-cold saline was performed for 1 min. Whole brain samples (excluding the cerebellum and pons) were collected at a 30-min time point after drug administration. Brain samples were immediately snap-frozen in liquid nitrogen and stored at −80 °C.

### Quantitation of the Studied Compound *In Vivo*

The frozen brains were weighed and homogenized with 50 mM Tris-HCl buffer (pH 7.4) 1:5 (w/v) by a bead mill homogenizer (Omni Bead Ruptor 24 Elite homogenizer with a BR Cryo cooling unit, Omni International, Kennesaw, GA) with ceramic beads at 4 °C. The proteins were precipitated by diluting the homogenate 1:9 (v/v) with acetonitrile containing 0.1% (v/v) formic acid and the internal standard, labetalol. Samples were then centrifuged at 14 000*g* for 10 min at 4 °C. Supernatants were transferred into HPLC vials for analysis by liquid chromatography-tandem mass spectrometry (LC–MS/MS).

Drug concentrations in the brain were analyzed using an Agilent 1200 Series Rapid Resolution LC System with an Agilent Zorbax RRHD SB-C18 column (50 mm × 2.1 mm, 1.8 μm) and an Agilent 6410 triple quadrupole mass spectrometer equipped with an electrospray ionization (ESI) source (Agilent Technologies, Palo Alto, CA). The LC eluents were water (eluent A) and acetonitrile (eluent B), both containing 0.1% (v/v) formic acid. Analytes were separated with the following gradient: 0–0.3 min: 20% B, 0.3–1 min: 20% B → 95%, 1–5 min: 95% B, 5–5.5 min: 95% B → 20%, and 5.5–8 min: 20% B. The LC flow rate was 0.4 mL/min, the column temperature was set to 40 °C, and the sample injection volume was 5 μL. The LC–MS/MS data acquisition was performed in a positive ion mode with the following conditions: drying gas flow of 8.0 l/min with a temperature of 300 °C, nebulizer gas pressure of 40 psi, and a capillary voltage of 4 kV. The followed MRM transitions were 1013.7 → 777.7, 1013.7 → 731.6, and 1013.7 → 208.9 for the prodrug, 329.0 → 294.0 and 329.0 → 162.0 for labetalol (internal standard), and 255.0 → 209.0 for ketoprofen. Fragmentor voltages were 230, 70, and 100 V, respectively. The respective collision energies were 26, 28, and 45 V for prodrug **2**, 10 V for both labetalol products, and 10 V for ketoprofen. The data acquisition software was Agilent MassHunter Workstation software (version B.03.01), whereas Quantitative Analysis software (version B.09.00) was used for data processing and analysis. The lower limit of quantification for both prodrug **2** and ketoprofen was 0.1 nmol/g. The method was linear, selective, accurate, and precise in the calibration range of 0.1–300 nmol/g.

### Molecular Modeling and Proposed Binding Mode

#### Homology Model and Protein Preparation

The homology model for OATP1C1 was downloaded from the AlphaFold database https://alphafold.ebi.ac.uk/entry/Q9NYB5.(46,47) Residues at the N- (1-38) and C-termini (670-712) showed low to very low confidence scores (according to the AlphaFold Web site) displaying extended unfolded loops, so these residues have been deleted from the initial model. The quality of the model was evaluated with the Ramachandran plot implemented in PROCHECK (Supporting Information Figure S3).^48^ About 92% of the residues were found to be in the most favored region and 7.9% in the additional allowed region, which shows the good quality of the model. The model was prepared using a protein preparation wizard in Maestro (Schrödinger 2021.4).^49^ In the preprocess step, missing hydrogens were added and bond orders were assigned. The hydrogen bonding network has been optimized with protonation states generated by PROPKA at a pH of 7.4. Minimization of all atoms was performed for the model while converging the heavy atoms from the previous iteration to RMSD of 0.30 Å using an OPLS4 force field.^50^ Homology models for OATP1A2 (human), oatp1c1 (mouse), and oatp1a4/1a5/1a6 (mouse) were obtained from the AlphaFold database and prepared in the same way as mentioned above.

#### Binding Pocket Prediction

SiteMap was employed to predict the possible binding sites from the prepared proteins.^51^ The site having the highest Dscores and occupying the central pore region of the transporter was selected for grid generation and docking. This binding pocket is consistent with work from Adla et al.^11^ and Tonduru et al. (2023).^12^

#### Ligand Preparation and Molecular Docking

Prodrug designs (**1**–**8**), along with the natural substrates and inhibitors, were drawn using a 2D sketcher in Maestro, and low-energy 3D configurations were generated using LigPrep.^52,49^ By employing LigPrep, hydrogens were added, and all possible ionization states were produced using Epik at pH 7.0 (±2.0); possible tautomers and all stereoisomers were created using the OPLS4 force field.^53^ Amino acid *s*-like configurations were selected for all of the compounds with a single stereo center in agreement with the T_4_ configuration and both *S,S* and *S,R* configuration for racemic mixtures. The prepared ligands were docked using the Glide Docking tool, with a Standard Precision (SP) protocol, ligand sampling as flexible, and other settings were kept as default in the panel.^54^ Ligands were docked within the predicted binding pocket by SiteMap with a box limit of 20 Å. The poses were examined and selected based on the interactions and the placement of the amino acid part of the compounds in the polar region and the hydrophobic part toward the extracellular region. The selected poses (*S* and *S,S* configurations were selected based on pose ranking and orientation) were further considered for MD simulations.

### Molecular Dynamics Simulations

MD simulations were performed with Desmond from the Schrödinger package.^55^ The protein–ligand complexes were treated with a predefined TIP3P^56,57^ solvation model, and preequilibrated dimyristoylphosphatidylcholine (DMPC) membrane at 300 K temperature. The membrane was placed perpendicular to the helices and pores of the protein. Periodic boundary conditions (PBC) were set with an orthorhombic box and buffer size of 10 Å in all directions. Chlorine ions were added to neutralize the whole system according to the charge in each protein–ligand complex. The OPLS4 force field^50^ was used for all of the system preparations and simulations. These setups are simulated for 500 ns with 5 replicates (each seed with different random initial velocities for atoms), in total 2.5 μs per ligand, unless stated otherwise. We used the NPT ensemble class at 300 K temperature and 1.01 bar pressure. Frames were recorded for every 1 ns of simulation. Before running the simulation production, all systems were energy minimized and underwent short MD simulations using the relax model system protocol for membranes incorporated in the Desmond MD panel. The RESPA integrator with default timesteps for near 2 fs, bonded 2 fs, and far 6 fs was used. The Nose–Hoover chain thermostat method^58,59^ and the Martyna–Tobias–Klein barostat^60^ method were used to maintain the temperature and pressure constant. Short-range Coulombic interactions were handled by a cutoff radius of 9 Å. Figures were generated using PyMOL (v2.5) graphics from Schrödinger.^61^

### Molecular Dynamics Simulations Analysis

MD trajectories were analyzed using the Simulation Interaction Diagram tool (Schrödinger, 2023), which calculates the protein–ligand interaction frequencies, root mean squared fluctuations (RMSF) (Supporting Information Figure S4), root mean squared deviation (RMSD) of protein (Supporting Information Figure S5), RMSD of ligand (Supporting Information Figure S6), ligand torsions, and further properties, with respect to time along the simulations.

### Principal Component Analysis

Principal component analysis of trajectories from the MD simulations was performed to determine the overall motion of the protein–ligand complexes. All of the trajectories from the simulations were merged into one trajectory file (total 27 μs) using the trj_merge.py script and aligning to the initial frame using the trj_align.py script. A Python script, trj_no_virt.py provided by Schrödinger, was used to convert the .cms and trajectory files to .pdb and .xtc files, which were used as input files for Gromacs (version 2021.4). Extracellular and intracellular loops were excluded, and only the transmembrane regions were considered further using the make_ndx script in Gromacs. Gromacs tool gmx covar was used to calculate and diagonalize the covariance matrix. The eigenvectors produced are analyzed and projected using gmx anaeig script in Gromacs. Mode vectors script from PyMOL v2.5.4 (Schrödinger LCC, New York, NY) was used to visualize the principal components generated by the previous step. Principal component analysis of all of the simulations revealed changes in the conformation of the transporter, and the information related could be found in the legends of Supporting Information Figure S7.

### MM-GBSA Binding Energy Calculations

Molecular mechanics with generalized Born and surface area (MM-GBSA) predicts the binding free energy of protein–ligand complexes, and the ranking of ligands based on the free energy could be correlated to the experimental binding affinities, especially in a congeneric series. Every fifth frame from the simulations (2500 ns for each compound) was considered for the calculations, which gave 500 data points for each compound. These were used as input files for the MM-GBSA calculations with the thermal_mmgbsa.py script from the Schrödinger package.

## Data Availability

All of the information and files related to *in silico* docking results and molecular dynamics-related data are available at Zenodo website at http://10.5281/zenodo.7861415.

## Supporting Information Available

Concentration-dependent cellular uptake of prodrugs **1**–**8**; principal component analysis from the overall simulation data; and ^1^H NMR, ^13^C NMR spectrum, and HPLC chromatogram of prodrugs **1**–**8** (PDF)
SMILES strings with compounds and the cellular uptake data (CSV)
PDB coordinates for the initial homology models were retrieved from the AlphaFold database under the identifiers AF-Q9NYB5-F1 (OATP1C1 model for human), AF- Q9ERB5-F1 (oatp1c1 mouse model), AF-P46721-F1(human OAPT1A2 model), AF-Q9EP96-F1 (oatp1a4 mouse model), AF- Q91YY5-F1 (oatp1a5 mouse model), and AF- Q99J94-F1 (oatp1a6 mouse model) (ZIP)

## Abbreviations Used

BBB: blood–brain barrier
DCF: diclofenac
DCM: dichloromethane
DIT: 3,5-diiodo-l-tyrosine
DMF: *N*,*N*-dimethylformamide
E217G: estradiol 17β-d-glucuronide
ES: estrone-3-sulfate
FFA: flufenamic acid
FLB: flurbiprofen
KPF: ketoprofen
*K*_m_: Michaelis–Menten constant
LAT1: l-type amino acid transporter 1
MCT8: monocarboxylate transporter 8
MD: molecular dynamics
MM-GBSA: molecular mechanics with generalized Born and surface area
MFS: major facilitator superfamily
NPX: naproxen
OATP: organic anion-transporting polypeptide
OATP1C1: organic anion-transporting polypeptide 1C1
PCA: principal component analysis
PD: prodrug
rT3: reverse triiodothyronine
SA: salicylic acid
SLC: solute carrier family
T3: triiodothyronine
T4: thyroxine
TH: thyroid hormone
THF: tetrahydrofuran
