# Supplementary material for: Targeting Glial Cells by Organic Anion-Transporting Polypeptide 1C1 (OATP1C1)-Utilizing l-Thyroxine-Derived Prodrugs
Source: J Med Chem. 2023 Nov 6;66(22):15094–114. doi: 10.1021/acs.jmedchem.3c01026 (PMC10683023; doi:10.1021/acs.jmedchem.3c01026)
Supplement: Supplementary file 1 — jm3c01026_si_001.pdf [file jm3c01026_si_001.pdf]

Supporting Information

Targeting glial cells by Organic anion transporting polypeptide 1C1 (OATP1C1) -utilizing *L*-Thyroxine-derived prodrugs

Arun Kumar Tonduru[1*,#], Seyed Hamed Maljaei[1#], Santosh Kumar Adla[1], Landry Anamea[1], Janne Tampio[1], Adéla Králová[1], Aaro J. Jalkanen[1], Catarina Espada[1], Inês Falcato Santos[1], Ahmed B. Montaser[1], Jarkko Rautio[1], Thales Kronenberger[1,2], Antti Poso[1,2,3], Kristiina M. Huttunen[1]

[1] School of Pharmacy, Faculty of Health Sciences, University of Eastern Finland, P.O. Box 1627, 70211 Kuopio, Finland.

[2] a. Department of Pharmaceutical and Medicinal Chemistry, Institute of Pharmaceutical Sciences, Eberhard-Karls-Universität, Tuebingen, Auf der Morgenstelle 8, 72076 Tuebingen, Germany. b. Tuebingen Center for Academic Drug Discovery & Development (TüCAD2), 72076 Tuebingen, Germany.

[3] a. Department of Internal Medicine VIII, University Hospital Tübingen, DE 72076 Tübingen, Germany; b. Cluster of Excellence iFIT (EXC 2180) "Image-Guided and Functionally Instructed Tumor Therapies", University of Tübingen, 72076 Tübingen, Germany.

# These authors contributed equally

*Corresponding author: University of Eastern Finland, P.O. Box 1627, 70211 Kuopio, Finland, E-mail: arun.tonduru@uef.fi

Table of Contents:

Figure S1. Concentration-dependent cellular uptake of prodrugs 1-8 (5-400 μM) into human glioma U-87MG cells. The data are presented as an intact prodrug (● filled circles) and released parent drug (○ hollow circles), mean ± SD (n = 3-6). 4

Figure S2. Concentration-dependent cellular uptake of prodrugs 1-8 (5-400 μM) into mouse primary astrocytes. The data are presented as an intact prodrug (● filled circles) and released parent drug (○ hollow circles), mean ± SD (n = 3-6). 5

Figure S3. Ramachandran plot showing 92% of residues in favoured region and 7.9% of residues in additional allowed regions. 6

Figure S4. A-J) Root Mean Squared Fluctuations (RMSF) of residues along the 500ns Molecular dynamics simulations in 5 replicates. 7

Figure S5. A-J) Root Mean Squared Deviations (RMSD) of protein along the 500ns Molecular dynamics simulations in 5 replicates. 8

Figure S6. A-I) Root Mean Squared Deviations (RMSD) of ligands along the 500ns Molecualr dynamics simulations in 5 replicates. 9

Figure S7. Principal component analysis from the overall simulation data A) PC1 extreme motion displaying two conformations open (wheat) and closed (grey) towards intracellular region. B) PC1 conformations showing the residues movement and interactions observed between Arg578(TM10) and Glu218(TM5), Lys585 (TM11) and Asp213 (TM4). C) Distance between Asp213 and Lys585 observed along the simulations D) Distances between Glu218 and Arg578 observed along the simulations. 10

Figure S8. Concentration-dependent cellular uptake of OATP-utilizing prodrugs 1-2 and 7-8 (5-100 μM; including the proportion of the released parent drugs), compared to their parent drugs and previously published LAT1-utilizing prodrugs A-C in human glioma U-87MG cells. The data are presented mean ± SD (n = 3-6). ...11

Figure S9. Overview of electrostatic potentials, hydrophobic and hydrophilic surfaces and pore lining shown in red spheres. A) Hydrophobic surfaces 1 (HP1) and 2 (HP2) surrounding the pore region. B) overall hydrophobic surface in lateral view on transmembrane helices. C) Lateral view of electrostatic potential showing small negatively charged surface (highlighted in black dotted circle) inside the pore. D) Intracellular view of electrostatic potential showing positive and negative surfaces. E) Lateral view of hydrophilic surfaces (intra and extracellular solvent exposed hydrophilic surfaces) with a small hydrophilic pocket inside the transmembrane pore. 12

Figure S10. Docked poses of each prodrug in green sticks, polar residues in cyan and hydrophobic residues in orange. A) docked pose of PD1 in OATP1C1 model B) docked pose of PD2 in OATP1C1 model C) docked pose of PD3 in OATP1C1 model D) docked pose of PD4 in OATP1C1 model E) docked pose of PD5 in OATP1C1 model F) docked pose of PD6 in OATP1C1 model G) docked pose of PD7 in OATP1C1 model H) docked pose of PD8 in OATP1C1 model I) docked pose of T4 in OATP1C1 model. 13

Figure S11. Interaction frequencies observed along the simulations for 2500ns. Negatively charged residues represented in red, positively charged residues in blue and others in black. The colour scale indicates red is less frequency and green is high frequency of interaction. 14

Figure S12. Identity matrix and comparison between human (1c1,1a2) and mouse (1c1, 1a4, 1a5 and 1a6) proteins. 14

Figure S13. Binding poses of prodrug designs in Human OATP1A2(A,B) and mouse oatp1c1(C,D). A) All binding poses of prodrugs overalayed in OATP1A2 shwoing consistent poses, B) Binding pose of PD2 showing interactions with human OATP1A2. C) Binding poses of prodrug designs overlayed in mouse oatp1c1 showing scattered and inconsistent poses among the designs, D) Binding pose of PD1 in mouse oatp1c1 showing hydrogen bonding interactions with Arg600, Glu204, Glu60, Lys56 and Tyr361 and pi-pi interactions with Phe369. 15

Figure S14. Binding poses of prodrug designs in Mouse oatp1a4(A,B), mouse oatp1a5(C,D) and mouse oatp1a6(E,F). A) Binding poses of prodrugs in oatp1a4 showing difference between diiodo and tetraiodo designs, where the later prefers vertical pose and former prefers horizontal pose, B) Binding pose of PD1 showing interactions with mouse oatp1a4, C) Binding poses of prodrug designs overlayed in mouse oatp1a5, D) Binding pose of PD5 in oatp1a5, E) Binding poses of prodrugs in mouse oatp1a6 showing consistent poses. F) Binding pose of PD6 showing interactions with the oatp1a6. 16

Figure S15. HPLC chromatogram of the background (ACN injection). 17

Figure S16. HPLC chromatogram of prodrug **1** (purity 97.80%). 17

Figure S17. HPLC chromatogram of prodrug **2** (purity 98.10%). 18

Figure S18. HPLC chromatogram of the parent drug for prodrugs **1**-**2**, commercial ketoprofen (purity 99.57%). 18

Figure S19. 1H NMR spectrum of prodrug **1** 19

Figure S20. ^{13}C NMR spectrum of prodrug **1**. 19

Figure S21. ^{1}H NMR spectrum of prodrug **2**. 20

Figure S22. ^{13}C NMR spectrum of prodrug **2**. 20

Figure S23. HPLC chromatogram of prodrug **3** (purity 96.01%). 21

Figure S24. HPLC chromatogram of prodrug **4** (purity 97.44%). 21

Figure S25. HPLC chromatogram of the parent drug for prodrugs **3**-**4**, commercial salicylic acid (purity 99.07%). 21

Figure S26. ^{1}H NMR spectrum of prodrug **3**. 22

Figure S27. ^{13}C NMR spectrum of prodrug **3**. 22

Figure S28. ^{1}H NMR spectrum of prodrug **4**. 23

Figure S29. ^{13}C NMR spectrum of prodrug **4**. 23

Figure S30. HPLC chromatogram of prodrug **5** (purity 96.66%). 24

Figure S31. HPLC chromatogram of prodrug **6** (purity 95.89%). 24

Figure S32. HPLC chromatogram of the parent drug for prodrug **5-6**, commercial naproxen (purity 99.78%). .24

Figure S33. ^{1}H NMR spectrum of prodrug **5**. 25

Figure S34. ^{13}C NMR spectrum of prodrug **5**. 25

Figure S35. ^{1}H NMR spectrum of prodrug **6**. 26

Figure S36. ^{13}C NMR spectrum of prodrug **6**. 26

Figure S37. HPLC chromatogram of prodrug **7** (purity 95.38%). 27

Figure S38. HPLC chromatogram of prodrug **8** (purity 95.18%). 27

Figure S39. HPLC chromatogram of the parent drug for prodrug **7-8**, commercial flurbiprofen (purity 99.75%). 27

Figure S40. ^{1}H NMR spectrum of prodrug **7**. 28

Figure S41. ^{13}C NMR spectrum of prodrug **7**. 28

Figure S42. ^{1}H NMR spectrum of prodrug **8**. 29

Figure S43. ^{13}C NMR spectrum of prodrug **8**. 29

Figure S44. Docking poses of DCF (A), FFA (B) and NRG (C) in OATP1C1. D) Interaction frequencies observed in 2500ns molecular dynamics simualtions for each inhibitor compound. 30

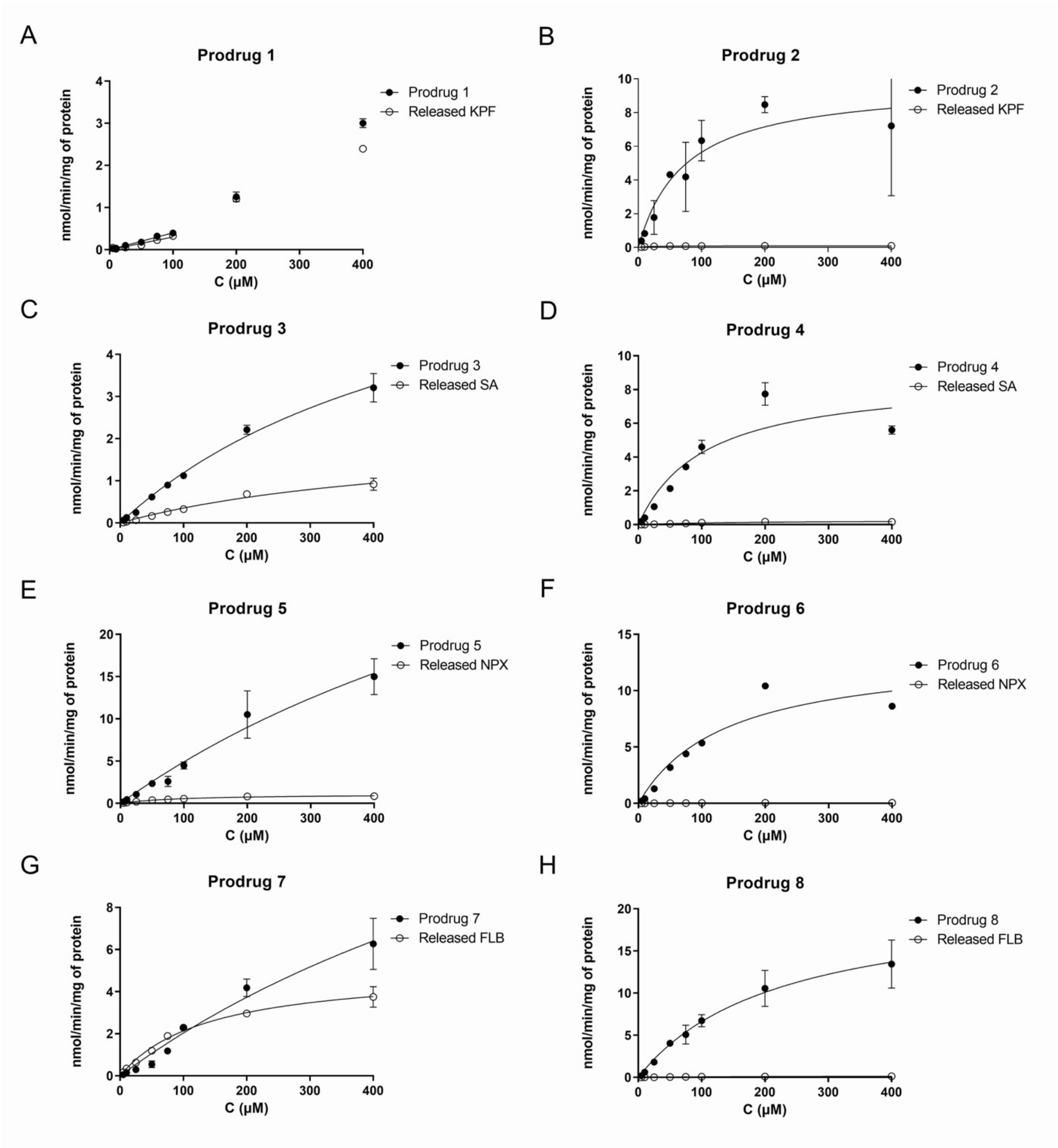

Figure S1. Concentration-dependent cellular uptake of prodrugs 1-8 (5-400 μM) into human glioma U-87MG cells. The data are presented as an intact prodrug (● filled circles) and released parent drug (○ hollow circles), mean ± SD (n = 3-6).

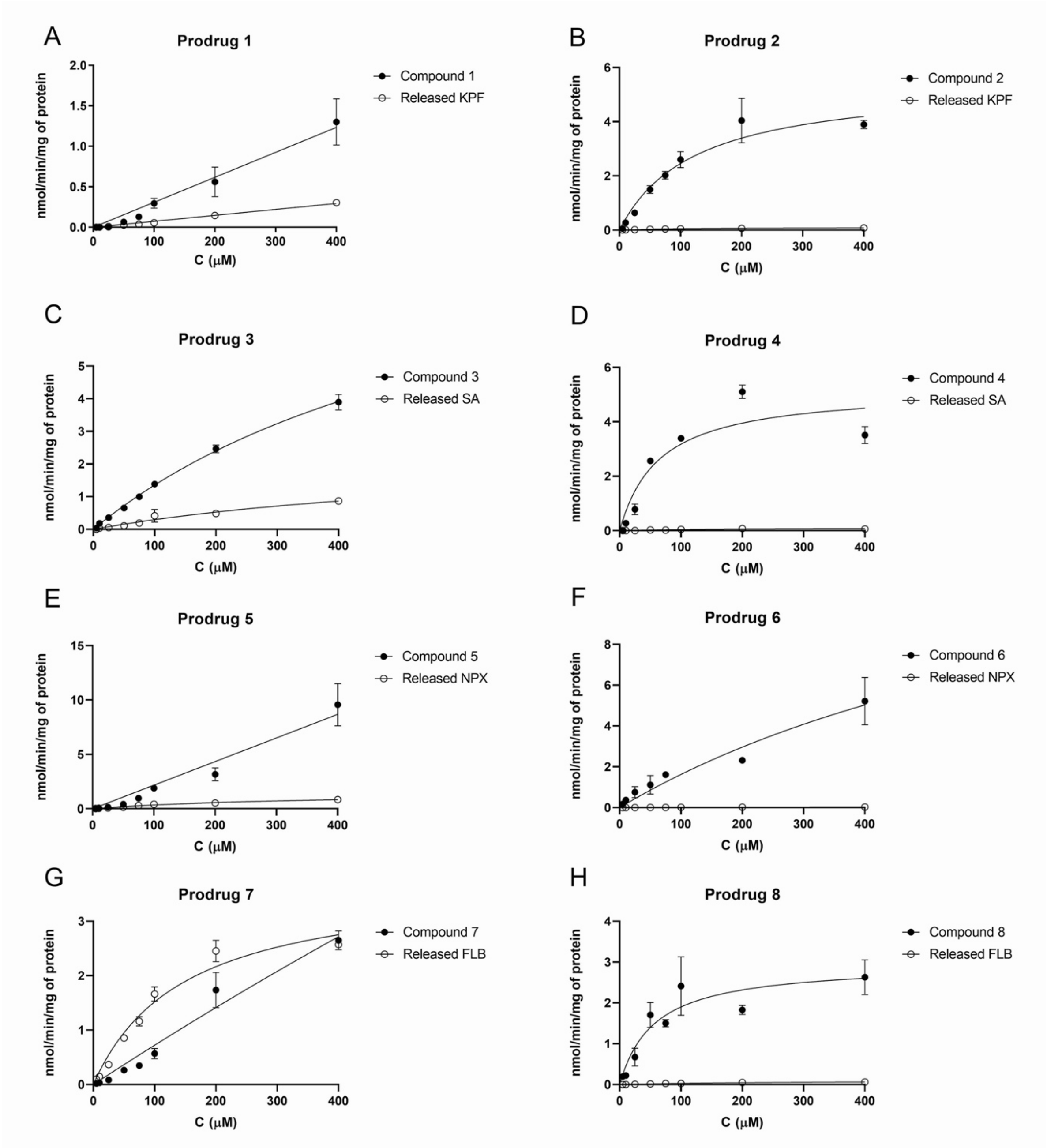

Figure S2. Concentration-dependent cellular uptake of prodrugs 1-8 (5-400 μM) into mouse primary astrocytes. The data are presented as an intact prodrug (● filled circles) and released parent drug (○ hollow circles), mean ± SD (n = 3-6).

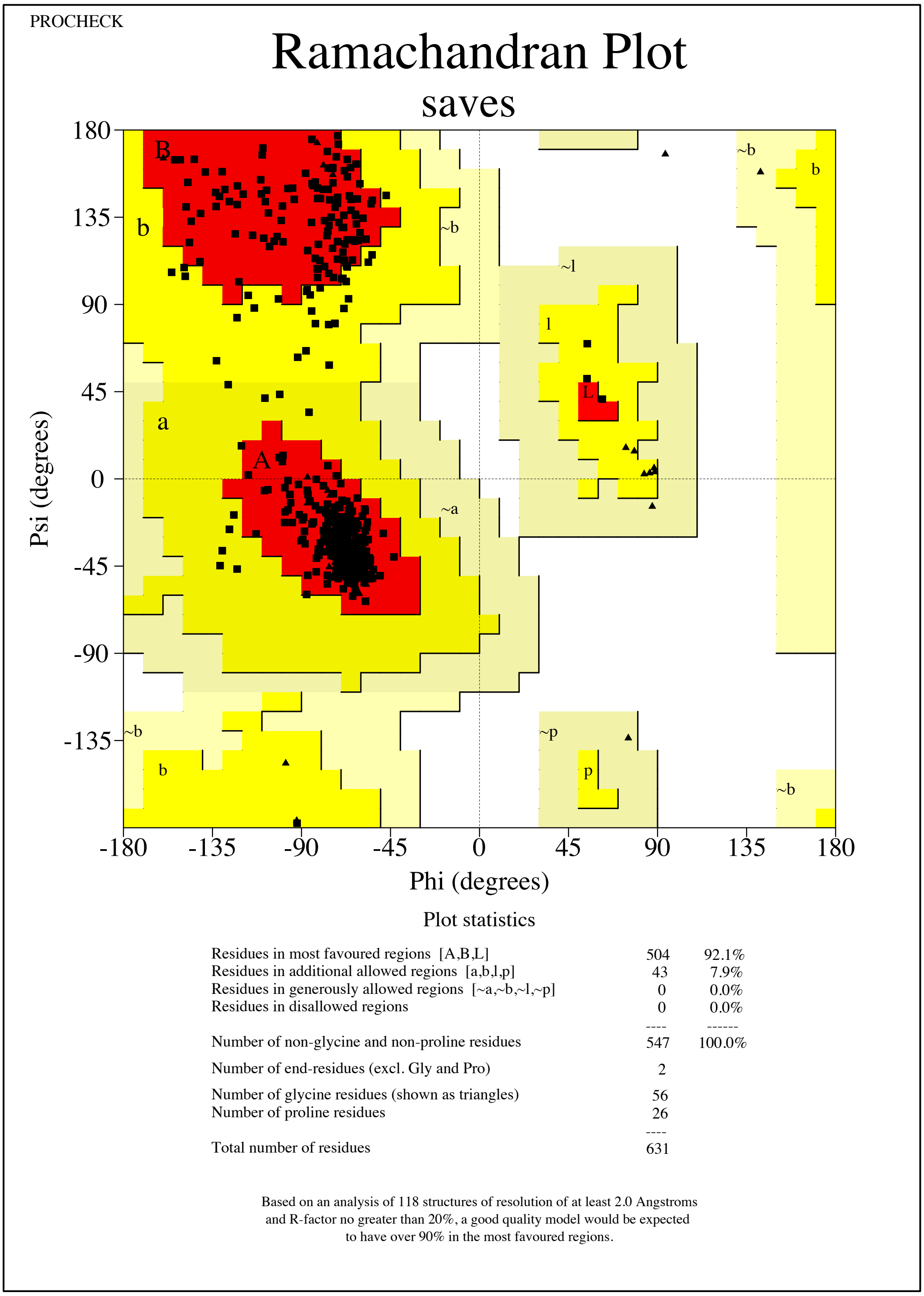

Figure S3. Ramachandran plot showing 92% of residues in favoured region and 7.9% of residues in additional allowed regions.

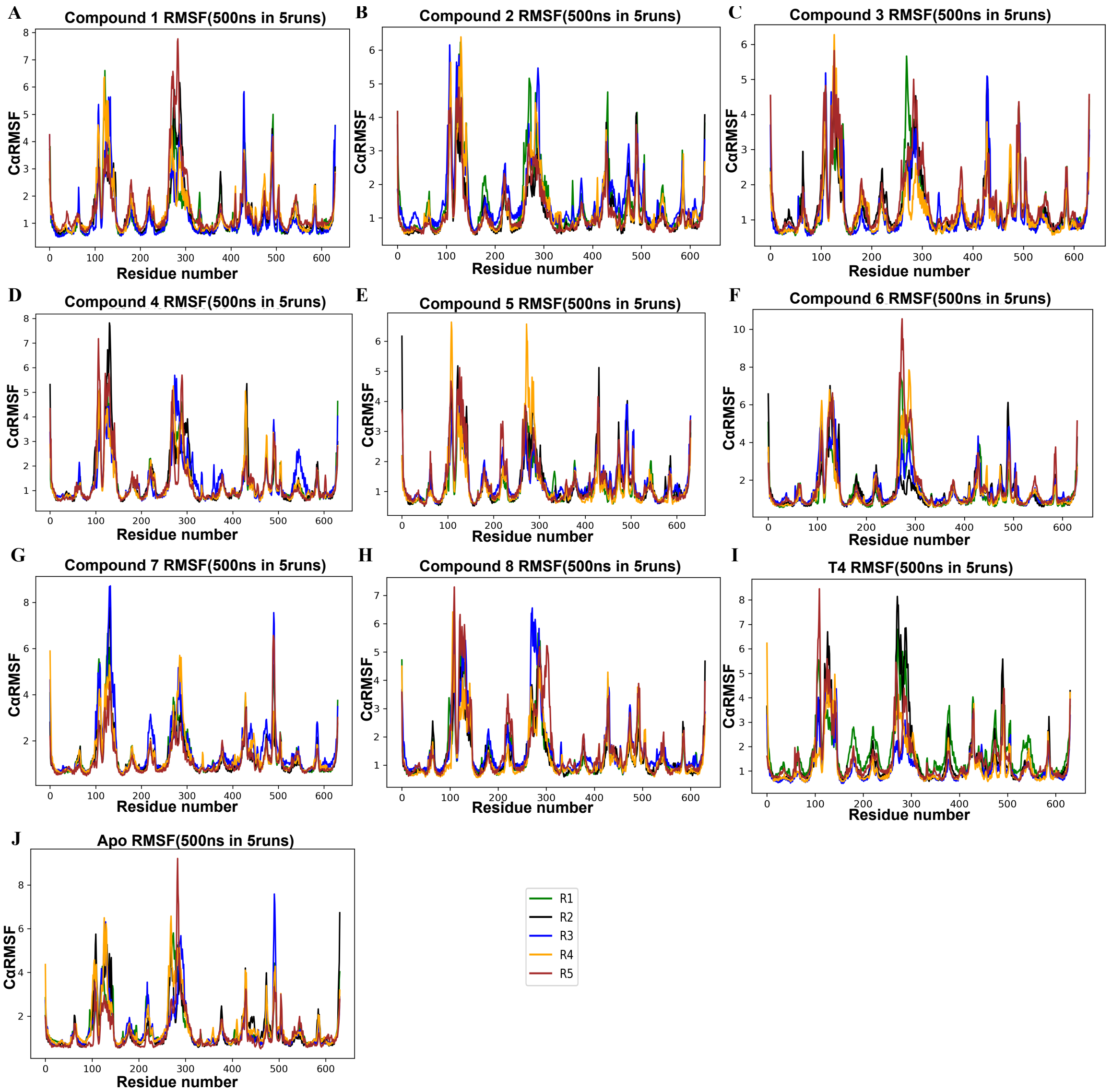

Figure S4. A-J) Root Mean Squared Fluctuations (RMSF) of residues along the 500ns Molecular dynamics simulations in 5 replicates.

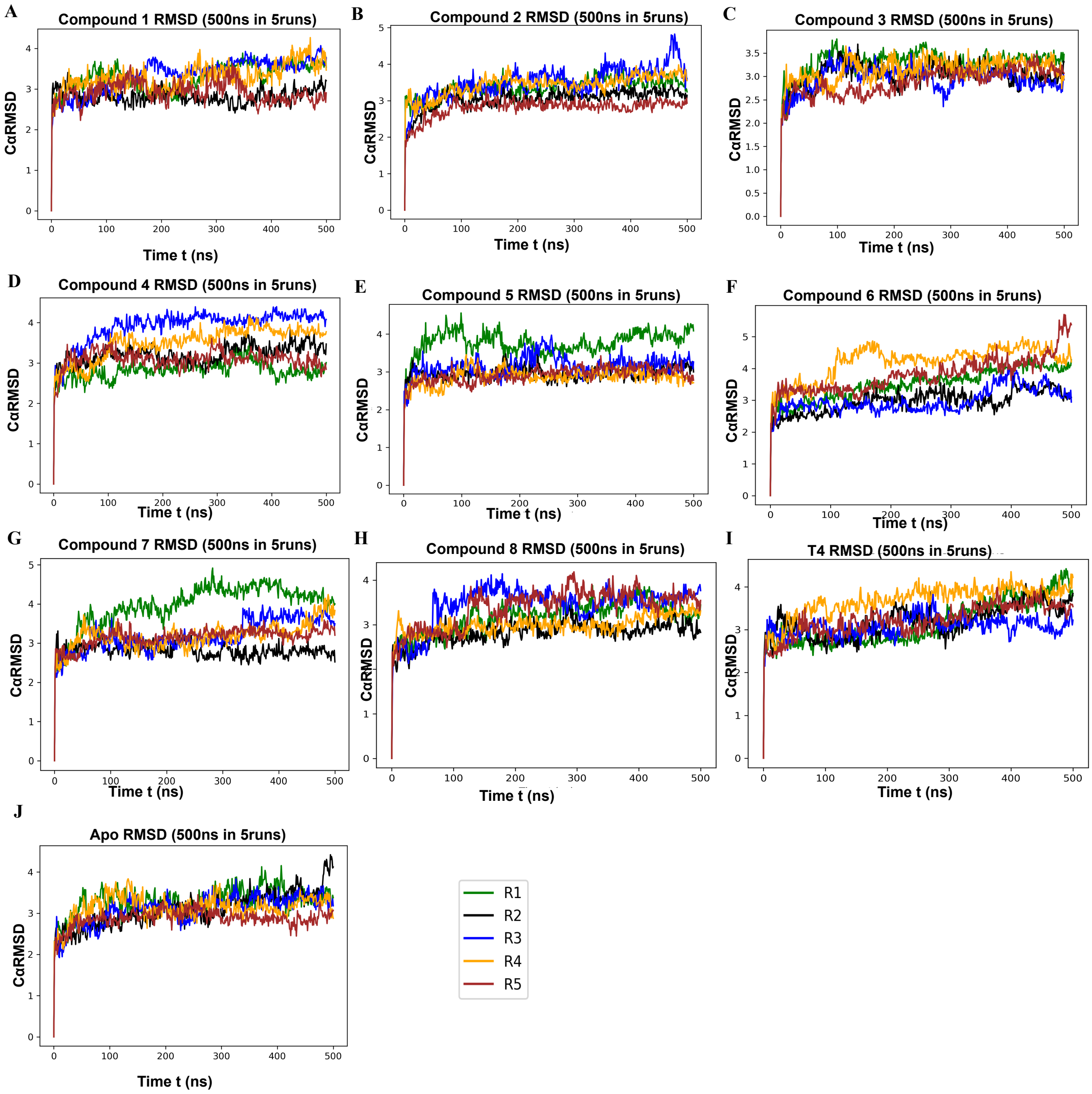

Figure S5. A-J) Root Mean Squared Deviations (RMSD) of protein along the 500ns Molecular dynamics simulations in 5 replicates.

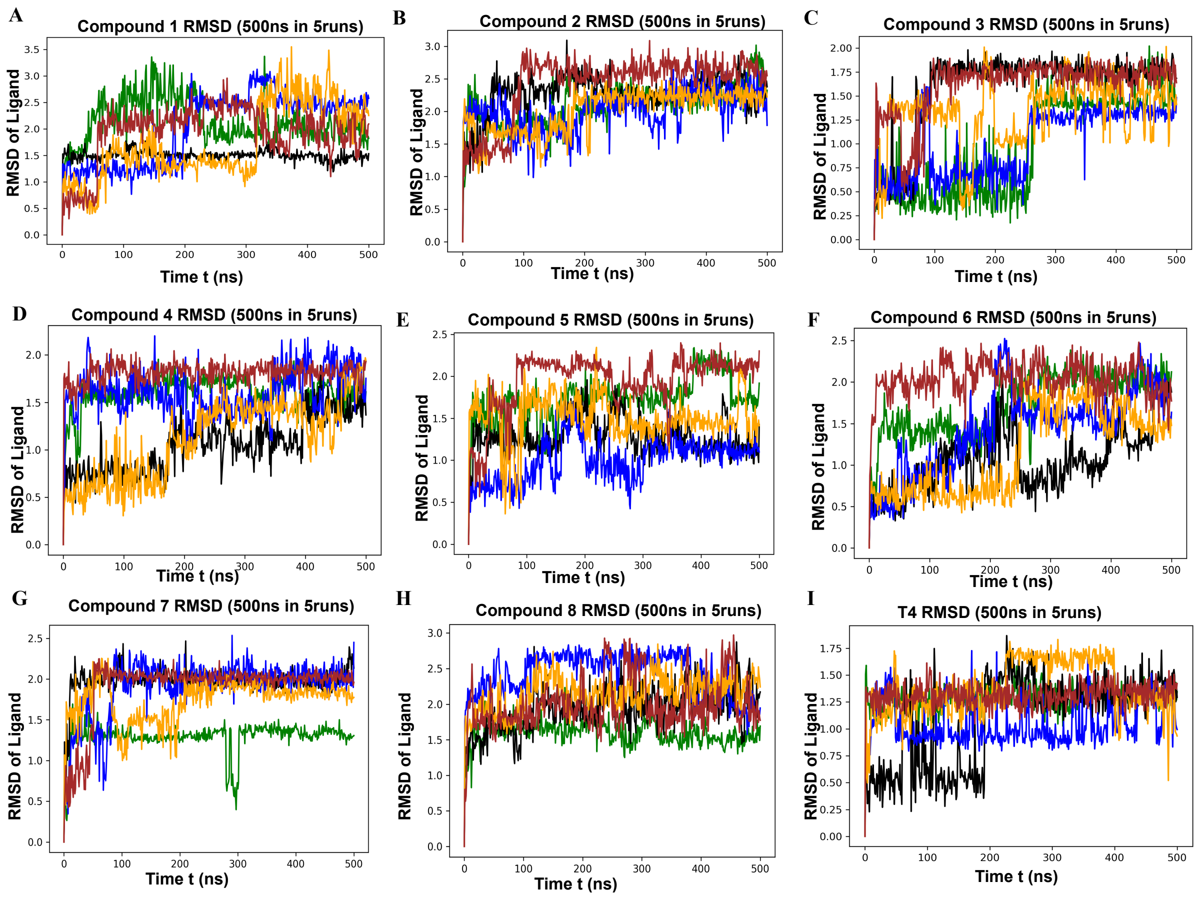

Figure S6. A-I) Root Mean Squared Deviations (RMSD) of ligands along the 500ns Molecualr dynamics simulations in 5 replicates.

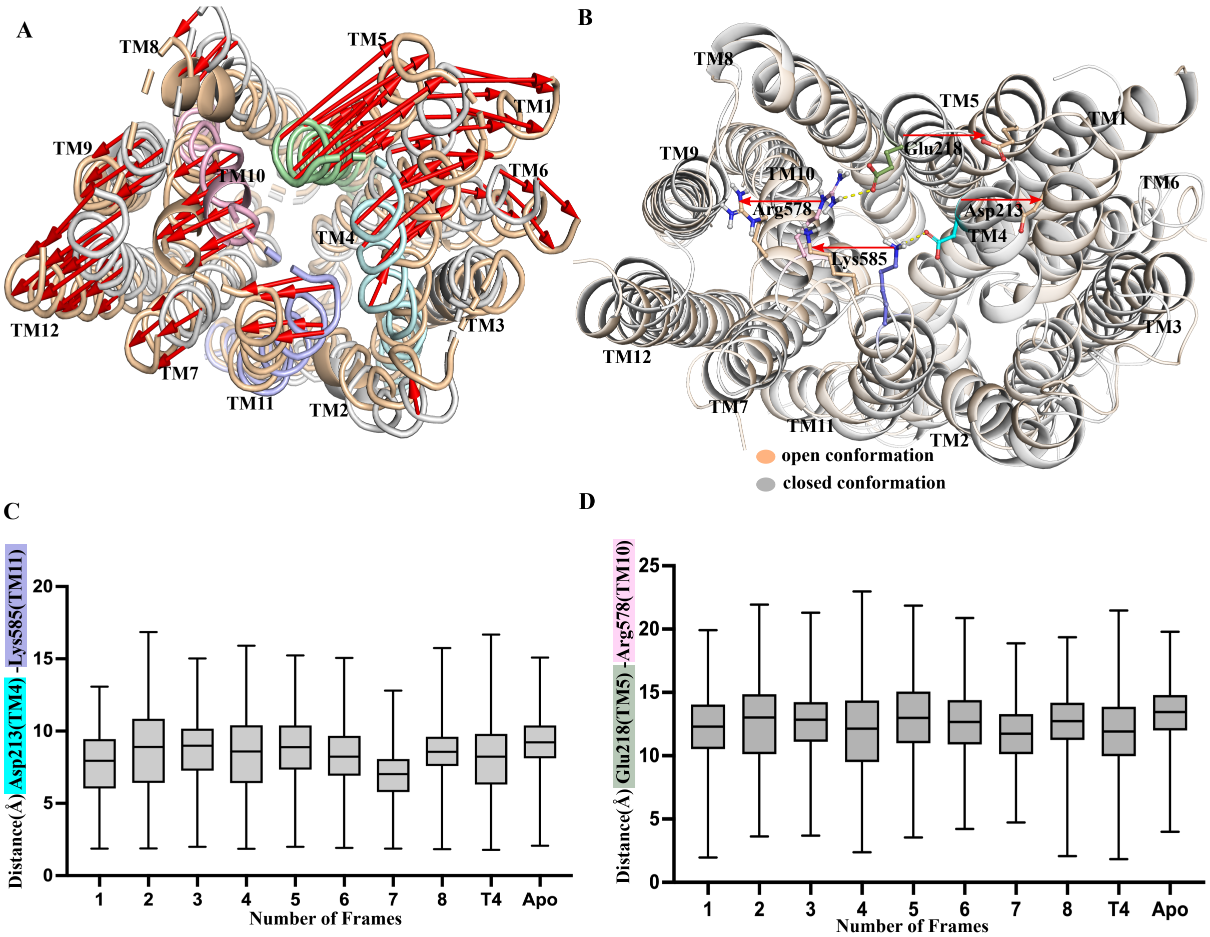

Figure S7. Principal component analysis from the overall simulation data A) PC1 extreme motion displaying two conformations open (wheat) and closed (grey) towards intracellular region. B) PC1 conformations showing the residues movement and interactions observed between Arg578(TM10) and Glu218(TM5), Lys585 (TM11) and Asp213 (TM4). C) Distance between Asp213 and Lys585 observed along the simulations D) Distances between Glu218 and Arg578 observed along the simulations.

Principal component analysis revealed closed and open conformations for OATP1C1:

Principal component analysis retrieved first two PC's with 7.7% and 2.7% respectively. The extreme motions observed in PC1 were shown in the Figure 3A. The l argest motions (based on the length of the arrows seen in mode vectors) were found in the transmembrane helices TM4, TM5, TM10 and TM11. The first and last frames from the PC1 were extracted and mode vectors were generated which shows two states (could be assumed open and closed) similar to the studies reported earlier in OATP1A2[1]. Based on the visual inspection and the findings above we further investigated the distances between helical pairs TM4-TM11 and TM5-TM10 for each prodrug. We have observed that in the terminals of TM4-TM11 and TM5-TM10 there are conserved positive and negative residues which are having interactions with each other and are leading to open and closed state at the intracellular region of the pore. In TM11 conserved positive residue Lys585 (conserved positive residue in all OATPs) interacts with negatively charged Asp213 (conserved in all OATPs except OATP1A2) on TM. Negatively charged residue Glu218 on TM5 interacts with conserved positively charged residue (Arg/Lys) Arg578 on TM10 (Figure 33B, 33C, 33D). These interactions lead us to hypothesize that TM4-TM11 and TM5-TM10 interactions are important to change from open to closed state during the transport of substrates through OATP1C1. From the recent cryoEM structure of human OATP1B1 publication it is evident that the TM4-TM11 residues Lys568-Asp198 are acting as intracellular gates[2]. The corresponding residues in OATP1C1 are Lys585 and Asp214, this would add to our hypothesis that TM4-TM11 involvement in gate closure.

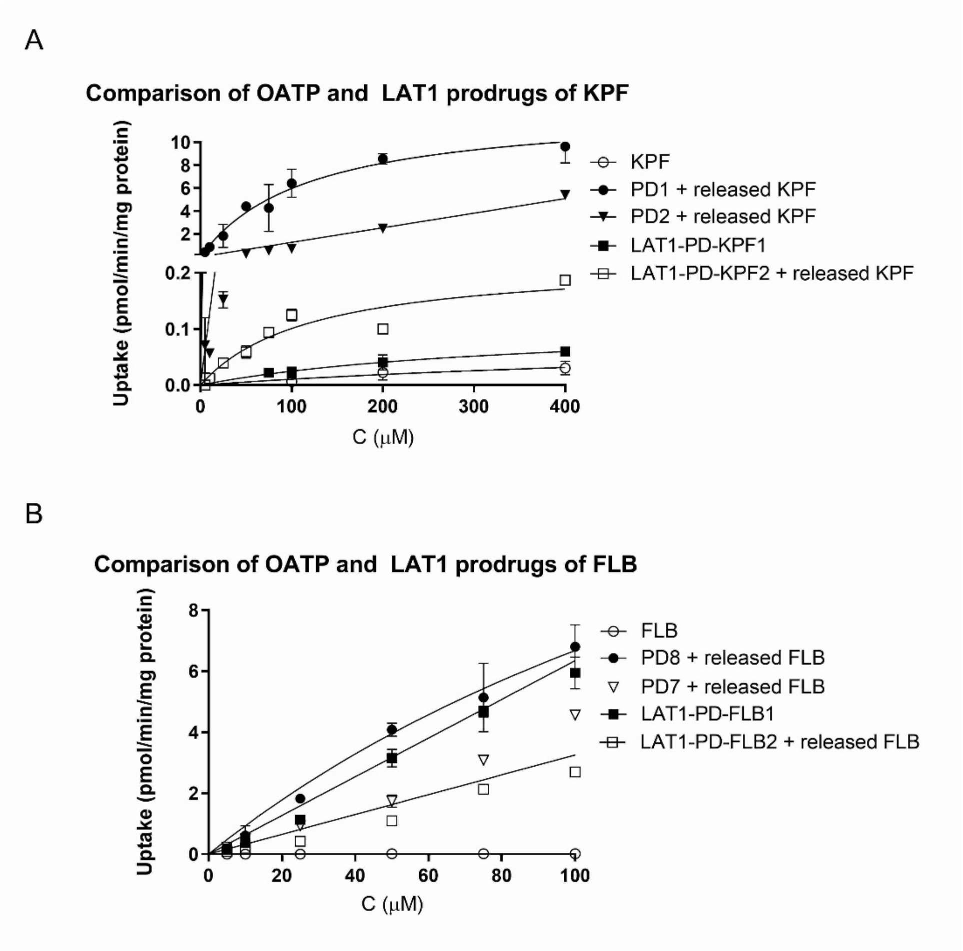

Figure S8. Concentration-dependent cellular uptake of OATP-utilizing prodrugs 1-2 and 7-8 (5-100 μM; including the proportion of the released parent drugs), compared to their parent drugs and previously published LAT1-utilizing prodrugs A-C in human glioma U-87MG cells. The data are presented mean ± SD (n = 3-6).

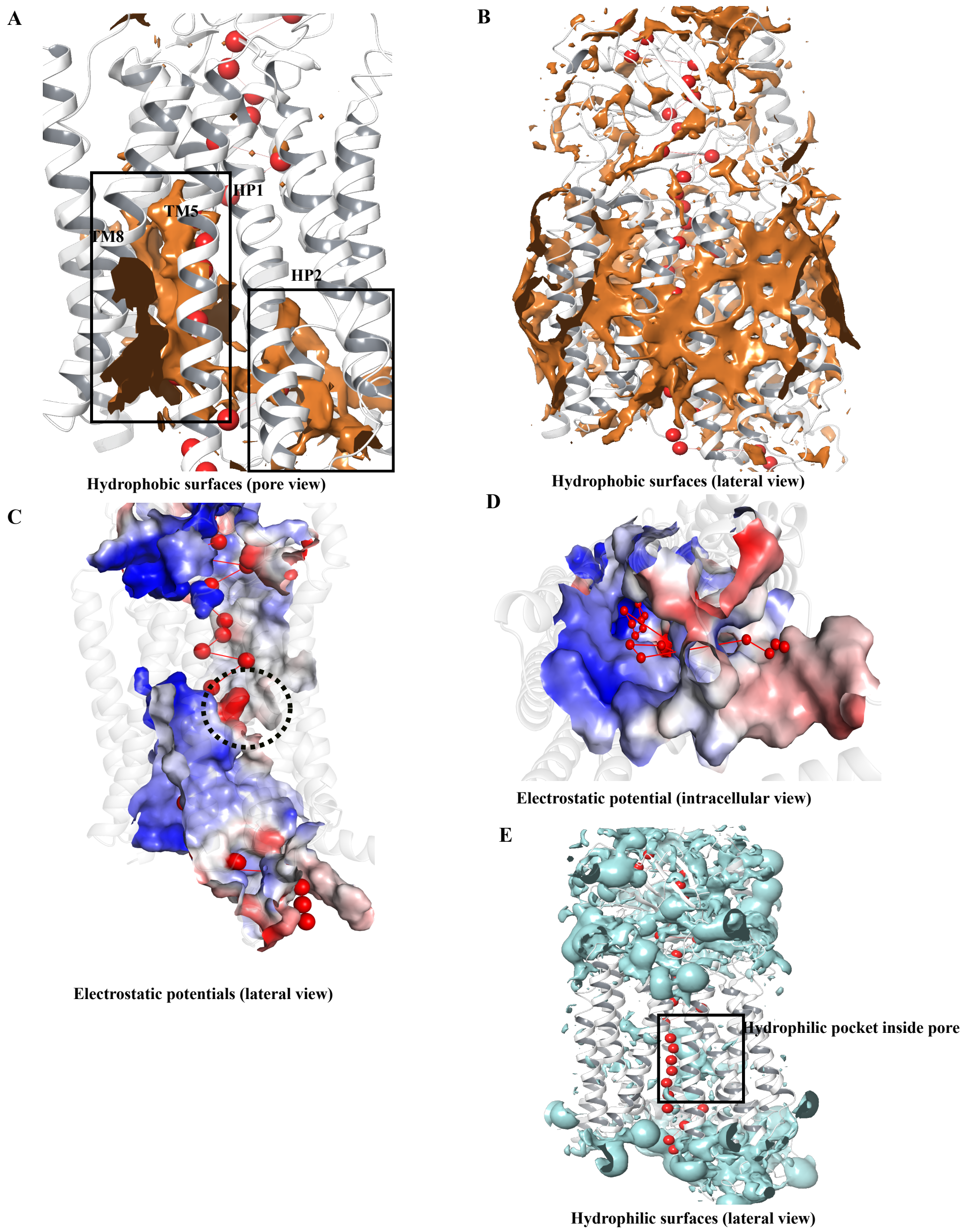

Figure S9. Overview of electrostatic potentials, hydrophobic and hydrophilic surfaces and pore lining shown in red spheres. A) Hydrophobic surfaces 1 (HP1) and 2 (HP2) surrounding the pore region. B) overall hydrophobic surface in lateral view on transmembrane helices. C) Lateral view of electrostatic potential showing small negatively charged surface (highlighted in black dotted circle) inside the pore. D) Intracellular view of electrostatic potential showing positive and negative surfaces. E) Lateral view of hydrophilic surfaces (intra and extracellular solvent exposed hydrophilic surfaces) with a small hydrophilic pocket inside the transmembrane pore.

We have explored the electrostatic potentials along the pore of the transporter using APBS electrostatics in PyMOL which revealed a positive potential throughout the pore except a small patch of negative potential near the TM1 (Glu60) and TM4 (Glu201) (Supporting information Figure S9, C). The intracellular region looks positive with some negative potentials near the solvent exposed surface. Hydrophobic/hydrophilic panel calculated the hydrophobic surfaces around the transmembrane helices and two distinct hydrophobic surface patches along the membrane pore. The hydrophobic surface around transmembrane regions depict the interaction of transmembrane region with phospholipid bilayer system. One of the hydrophobic surfaces is located to upper left side of pore region at interaction of TM5, TM7, and TM8 and another hydrophobic surface located to the right bottom of TM2, TM4 and TM11 (Supporting information Figure S9, A). Hydrophilic sites were observed at both extracellular and intracellular surface regions where there could be exposure to the solvent and a small pocket was observed in the pore region at the interface of TM1, TM4 and TM11 (Supporting information Figure S9, E).

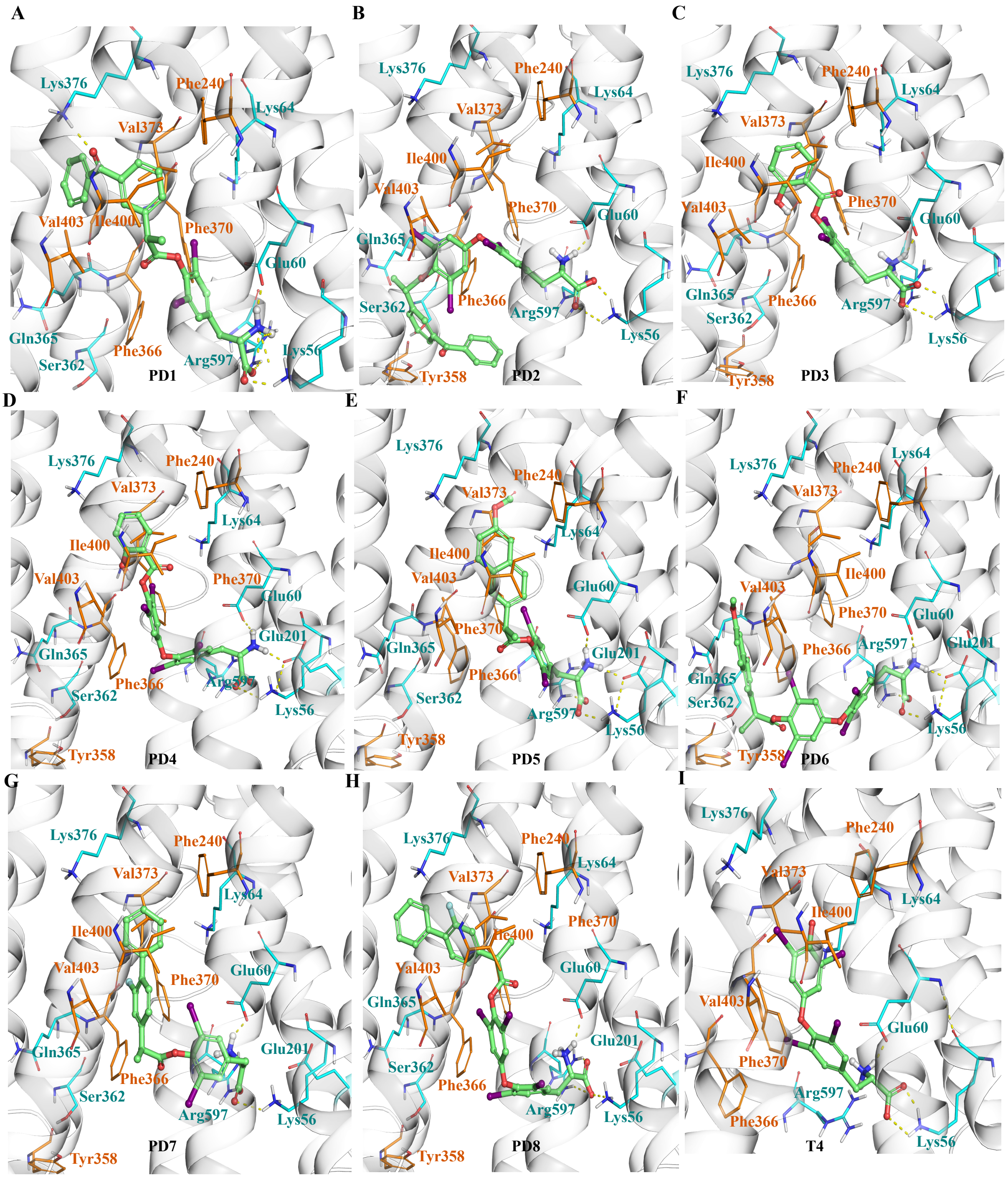

Figure S10. Docked poses of each prodrug in green sticks, polar residues in cyan and hydrophobic residues in orange. A) docked pose of PD1 in OATP1C1 model B) docked pose of PD2 in OATP1C1 model C) docked pose of PD3 in OATP1C1 model D) docked pose of PD4 in OATP1C1 model E) docked pose of PD5 in OATP1C1 model F) docked pose of PD6 in OATP1C1 model G) docked pose of PD7 in OATP1C1 model H) docked pose of PD8 in OATP1C1 model I) docked pose of T4 in OATP1C1 model.

		Residues	1	2	3	4	5	6	7	8	T4
Amino		Tyr53				15		18		13	
		Glu60	95	92	78	43	50	77	49	49	92
		Glu89	73	25			24	35	24	28	9
		Glu201	28	23		23	26	73	14	16	
	Carboxylate	Gln205	11			42	35	44	19	32	
		Gln229	12	30	57	53		46	45	45	17
		Arg597	84			32	58	74	34	39	6
		Lys56	11	56	19	33	29	70	19	37	11
		Lys64		31							9
Hydrophobic moeity		Ala232			21	11	19	13	12		35
		Phe240	11		33	24	22		33		
		Phe366	9	48				51		17	
		Leu369	22	26				43		24	
		Phe370	14						12	9	
		Met372	15							15	
		Val373	27		27	17	13		23	11	47
		Lys376	56	10	9	12	9			38	115
		Ile395	11		10		15		23		
		Asn399	9								11
		Ile400	33		27	23	18		12		
		Val403	31	38		13				20	59
		Ile407			11	24	15	23	10	19	
		Ser562			12						89
		Tyr573	16	45	28	56	34	11	30	51	22
		Tyr592		22		14				11	

Figure S11. Interaction frequencies observed along the simulations for 2500ns. Negatively charged residues represented in red, positively charged residues in blue and others in black. The colour scale indicates red is less frequency and green is high frequency of interaction.

**Identity_matrix**	oatp1c1_Mouse	OATP1C1_Human	OATP1A2_Human	oatp1a6_Mouse	oatp1a4_Mouse	oatp1a5_Mouse
oatp1c1_Mouse	100	83	44	42	43	43
OATP1C1_Human	83	100	44	41	42	44
OATP1A2_Human	44	44	100	66	73	73
oatp1a6_Mouse	42	41	66	100	75	76
oatp1a4_Mouse	43	42	73	75	100	84
oatp1a5_Mouse	43	44	73	76	84	100

Figure S12. Identity matrix and comparison between human (1c1,1a2) and mouse (1c1, 1a4, 1a5 and 1a6) proteins.

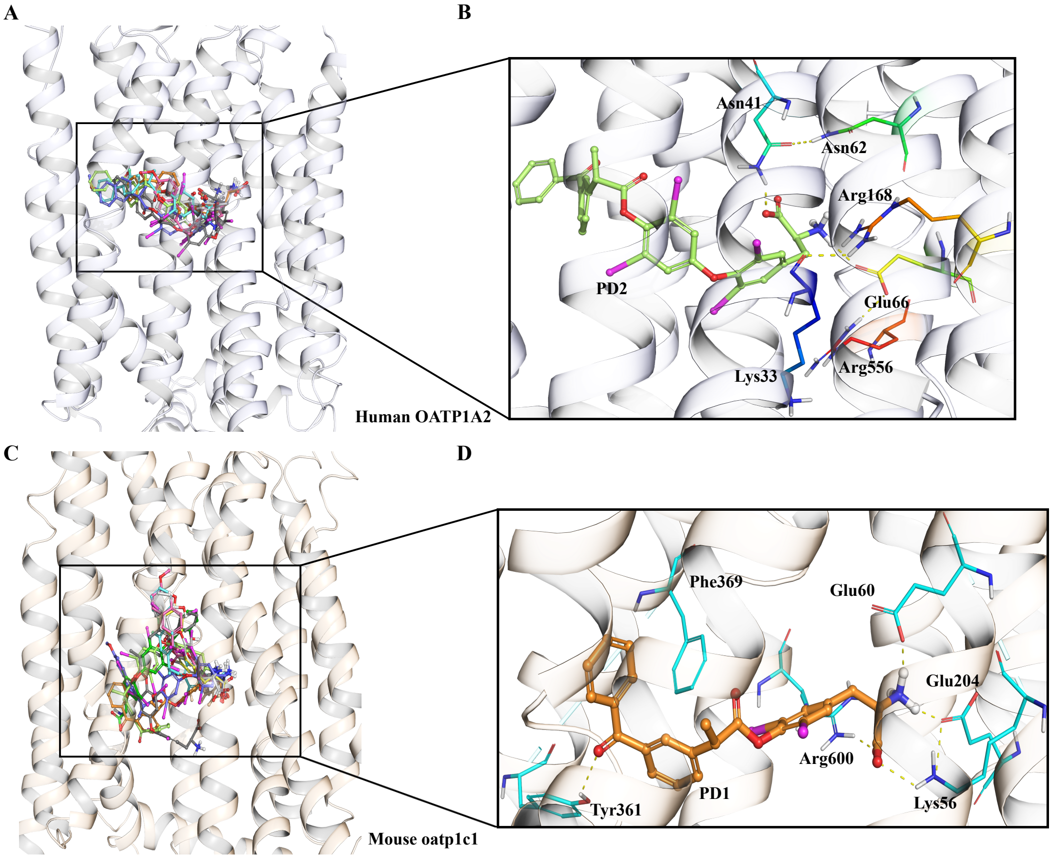

Figure S13. Binding poses of prodrug designs in Human OATP1A2(A,B) and mouse oatp1c1(C,D). A) All binding poses of prodrugs overalayed in OATP1A2 shwoing consistent poses, B) Binding pose of PD2 showing interactions with human OATP1A2. C) Binding poses of prodrug designs overlayed in mouse oatp1c1 showing scattered and inconsistent poses among the designs, D) Binding pose of PD1 in mouse oatp1c1 showing hydrogen bonding interactions with Arg600, Glu204, Glu60, Lys56 and Tyr361 and pi-pi interactions with Phe369.

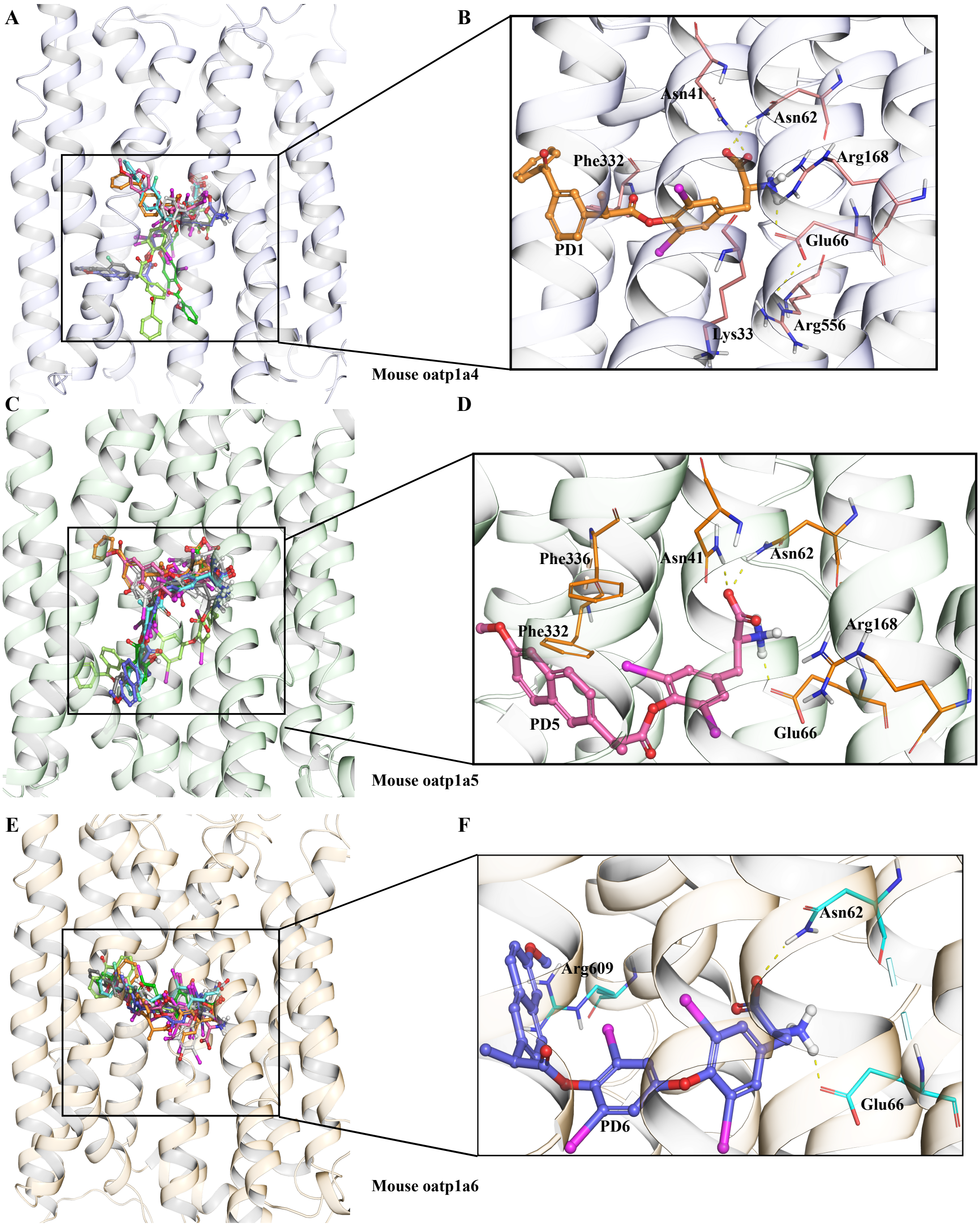

Figure S14. Binding poses of prodrug designs in Mouse oatp1a4(A,B), mouse oatp1a5(C,D) and mouse oatp1a6(E,F). A) Binding poses of prodrugs in oatp1a4 showing difference between diiodo and tetraiodo designs, where the later prefers vertical pose and former prefers horizontal pose, B) Binding pose of PD1 showing interactions with mouse oatp1a4, C) Binding poses of prodrug designs overlayed in mouse oatp1a5, D) Binding pose of PD5 in oatp1a5, E) Binding poses of prodrugs in mouse oatp1a6 showing consistent poses. F) Binding pose of PD6 showing interactions with the oatp1a6.

Characterization of the prodrugs 1-8:

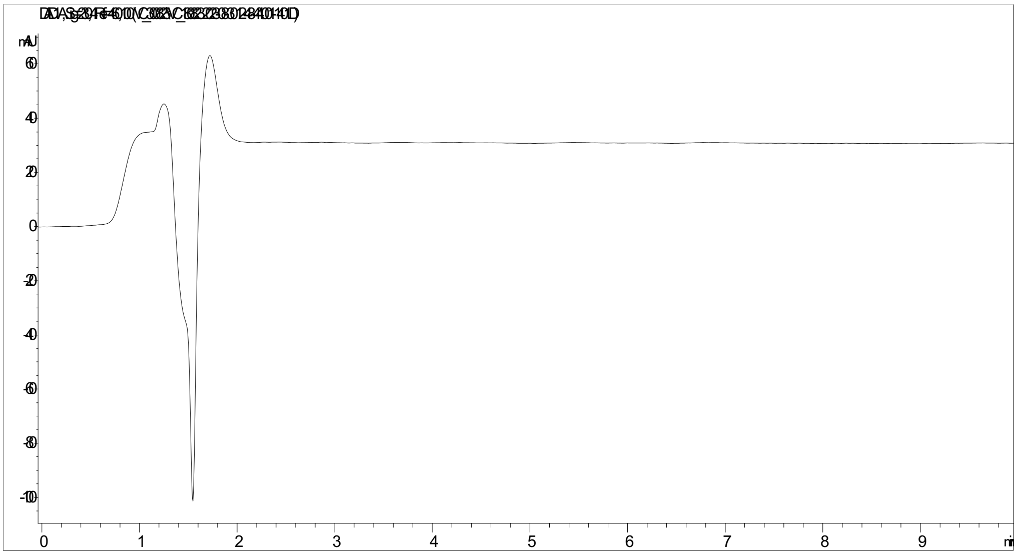

Figure S15. HPLC chromatogram of the background (ACN injection).

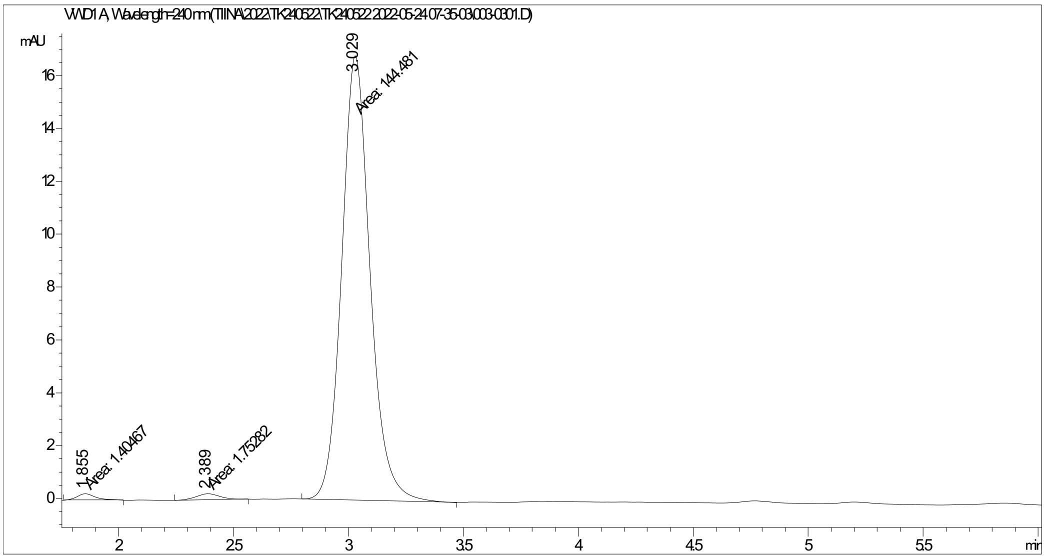

Figure S16. HPLC chromatogram of prodrug **1** (purity 97.80%).

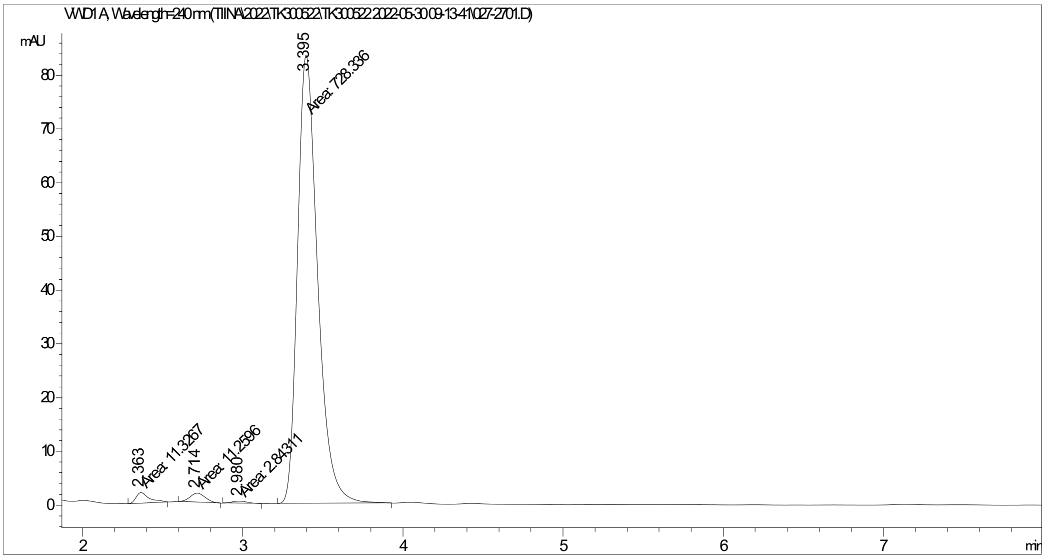

Figure S17. HPLC chromatogram of prodrug **2** (purity 98.10%).

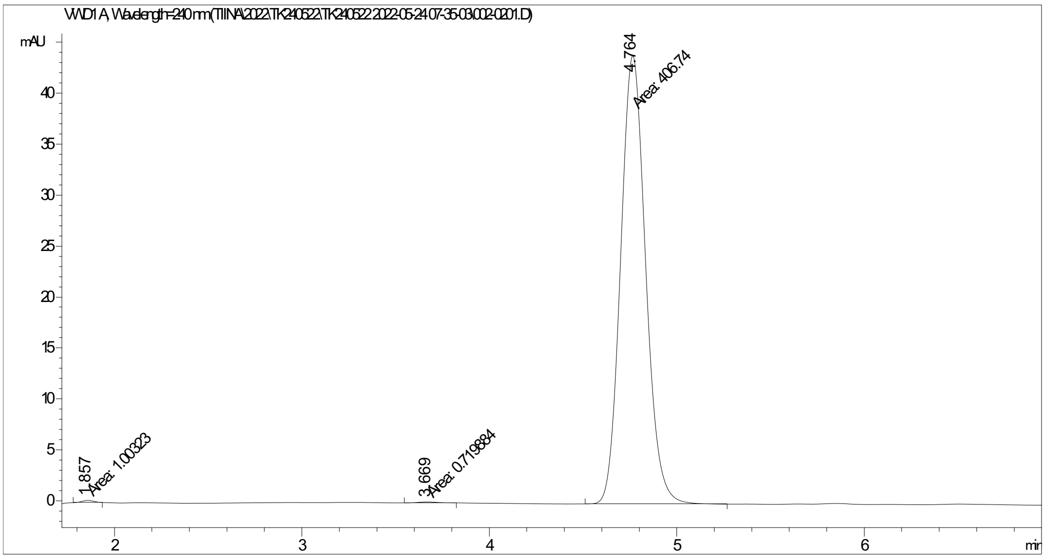

Figure S18. HPLC chromatogram of the parent drug for prodrugs **1-2**, commercial ketoprofen (purity 99.57%).

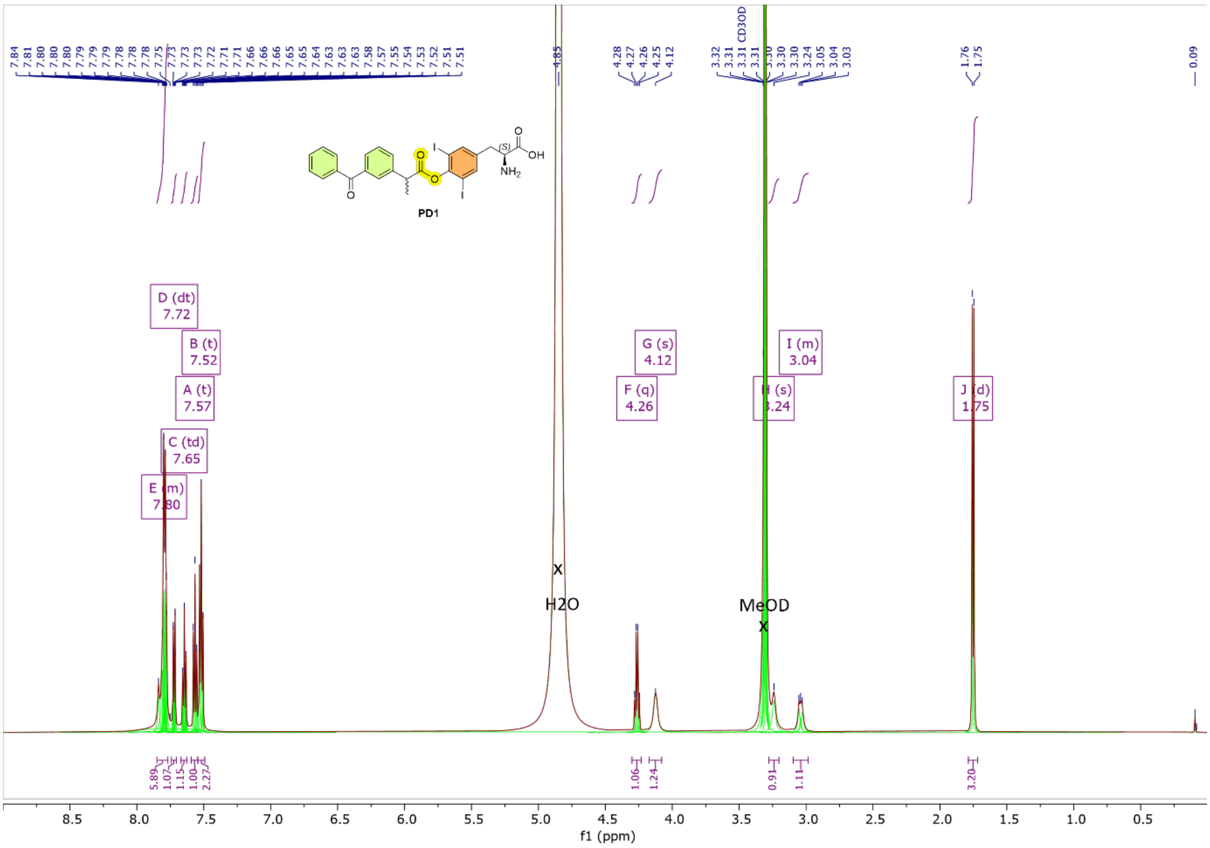

Figure S19. 1H NMR spectrum of prodrug **1**.

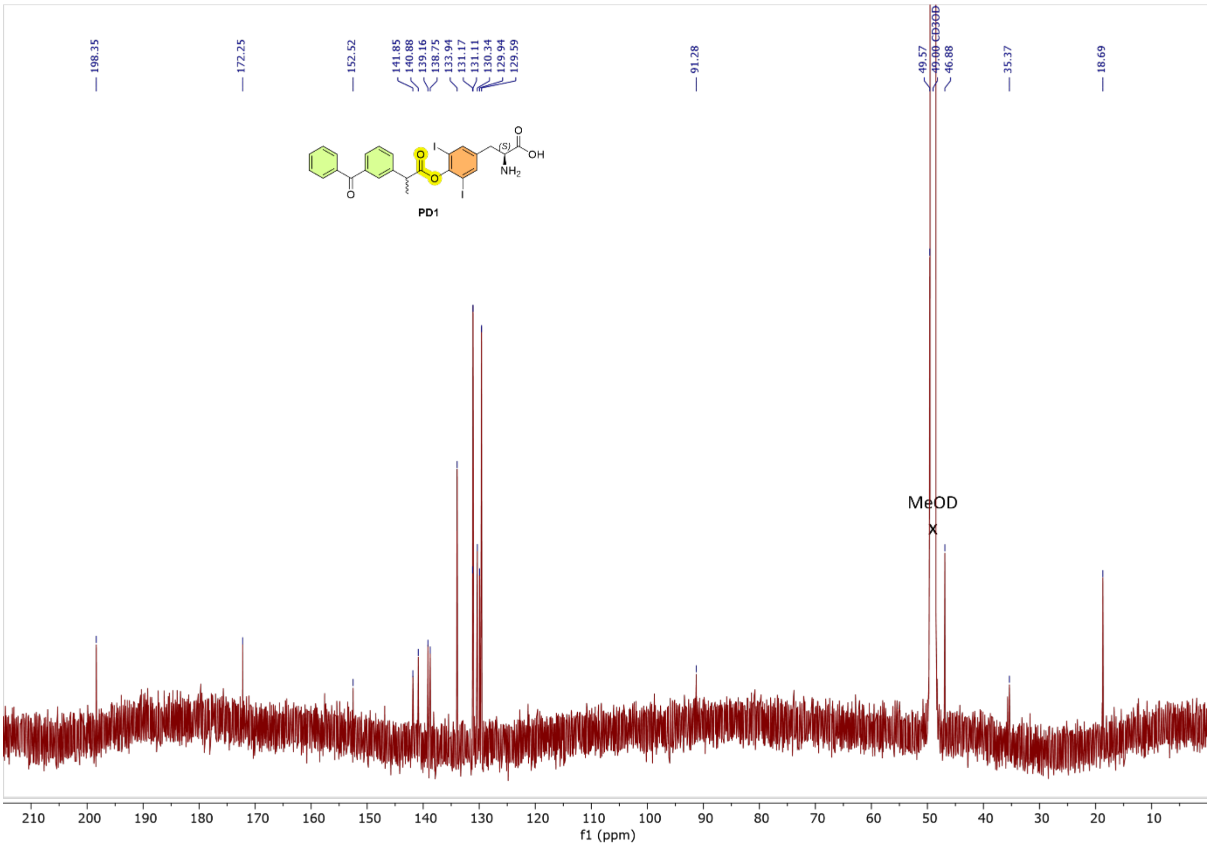

Figure S20. ^{13}C NMR spectrum of prodrug **1**.

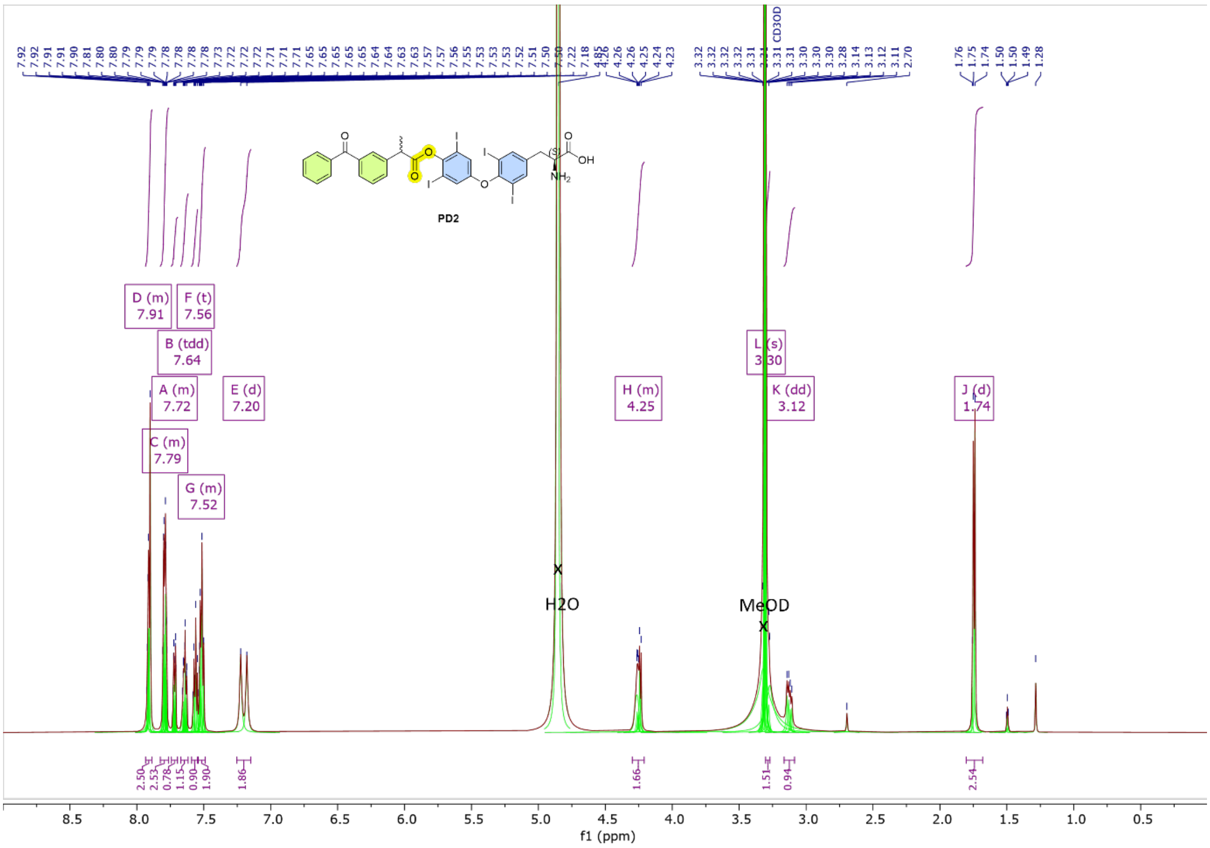

Figure S21. ^{1}H NMR spectrum of prodrug **2**.

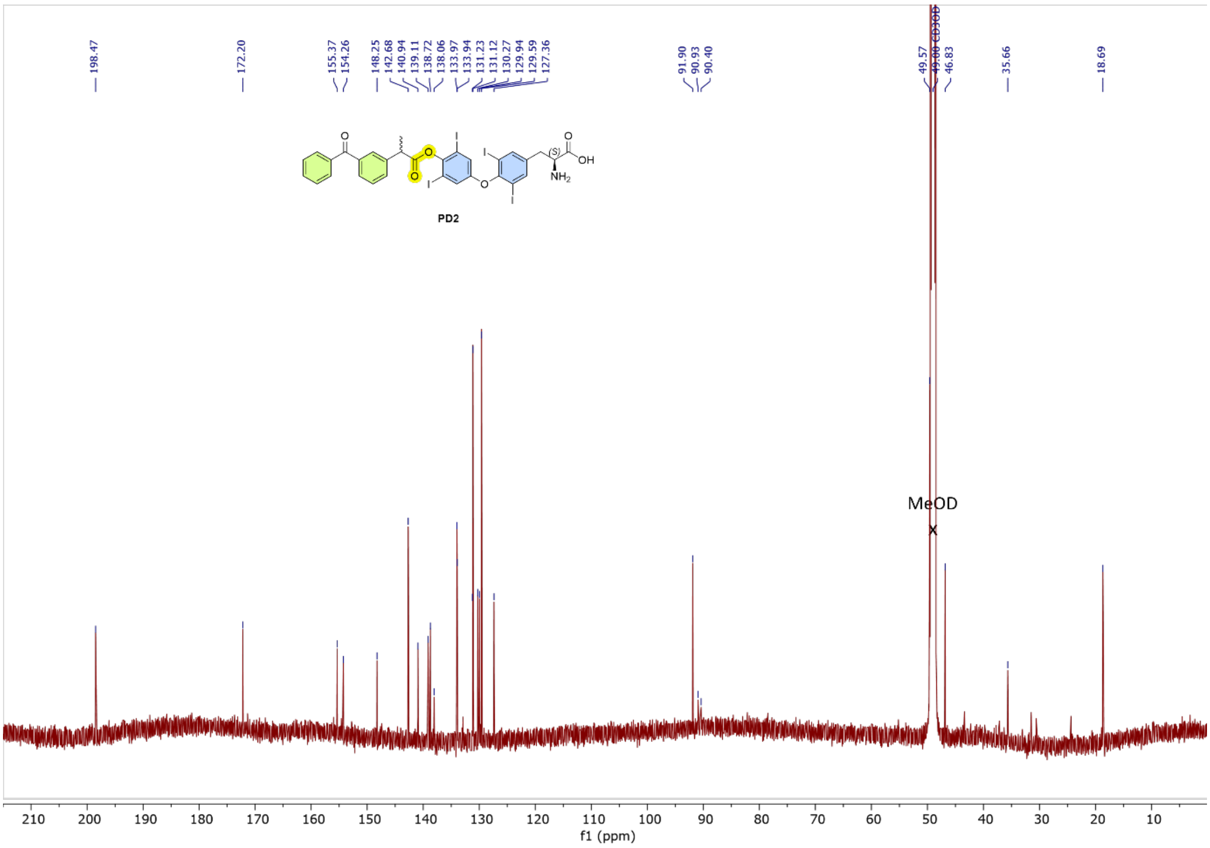

Figure S22. ^{13}C NMR spectrum of prodrug **2**.

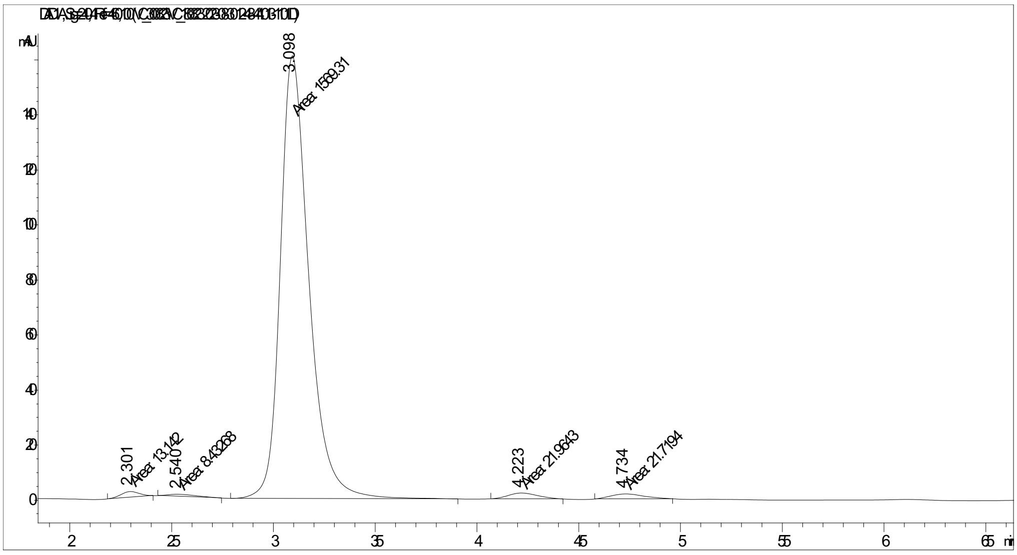

Figure S23. HPLC chromatogram of prodrug **3** (purity 96.01%).

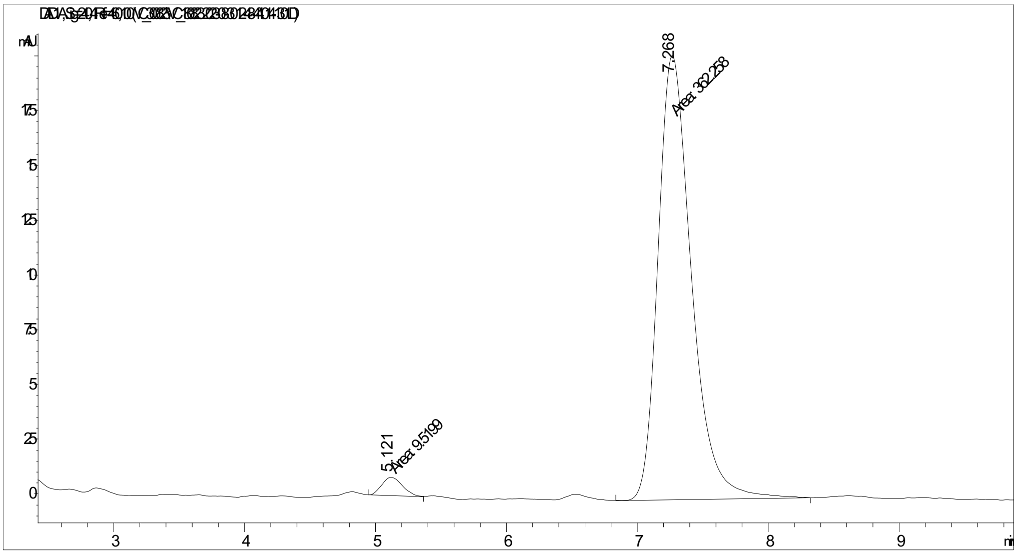

Figure S24. HPLC chromatogram of prodrug **4** (purity 97.44%).

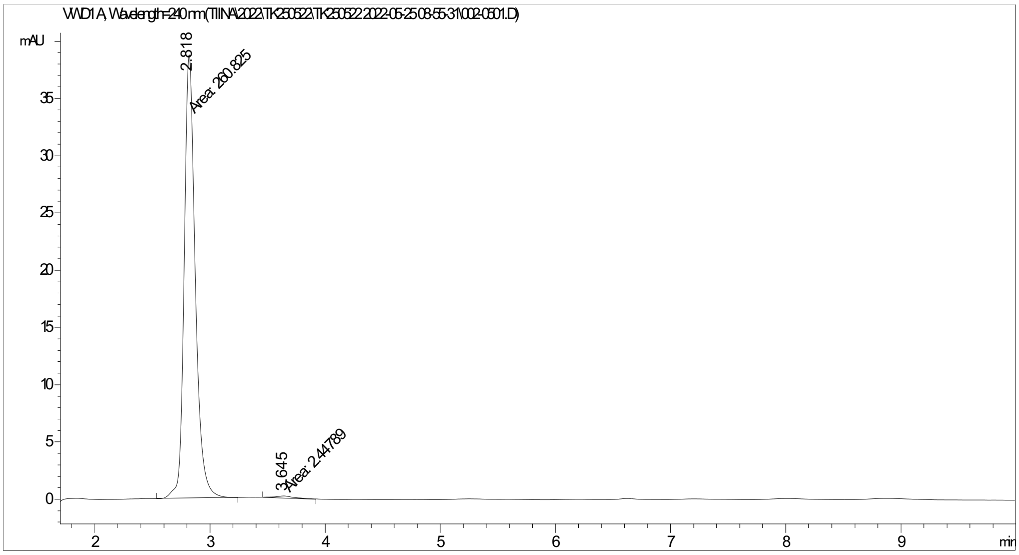

Figure S25. HPLC chromatogram of the parent drug for prodrugs **3-4**, commercial salicylic acid (purity 99.07%).

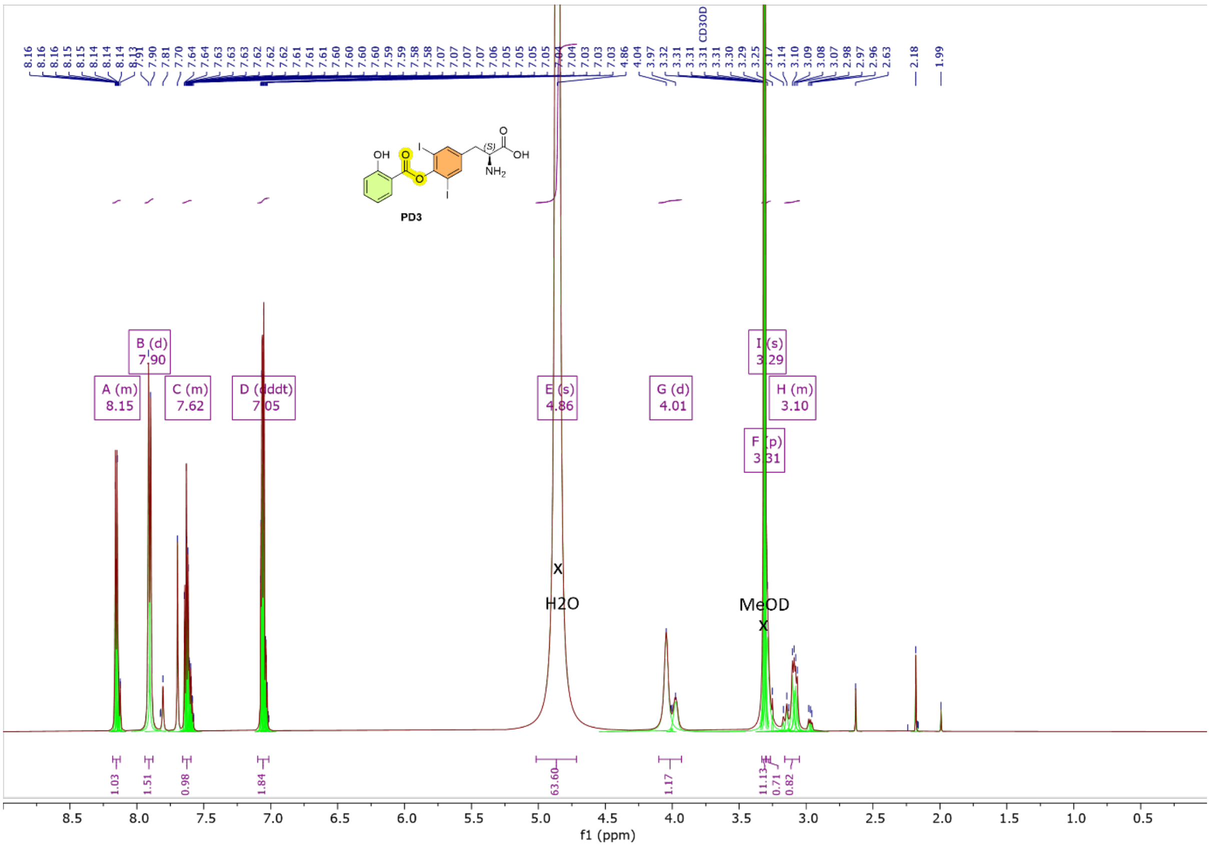

Figure S26. ^{1}H NMR spectrum of prodrug **3**.

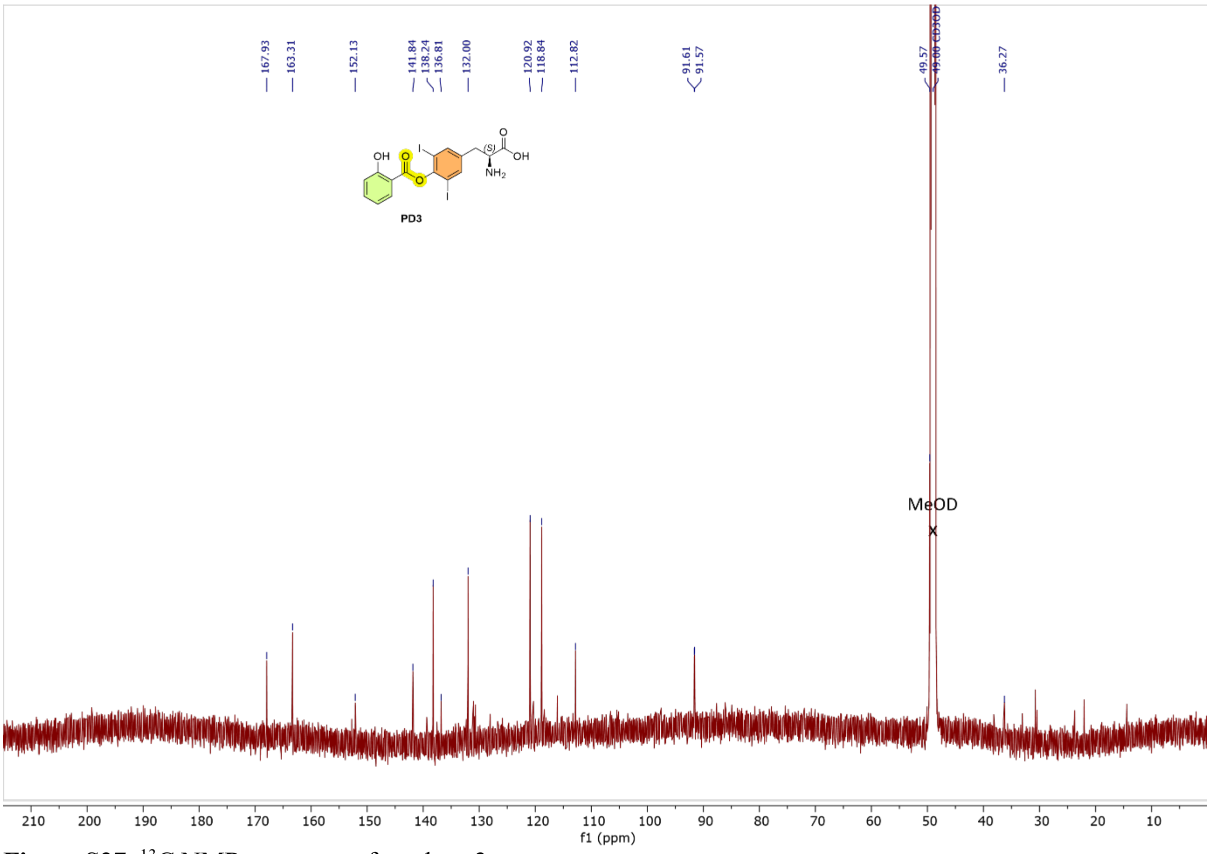

Figure S27. ^{13}C NMR spectrum of prodrug **3**.

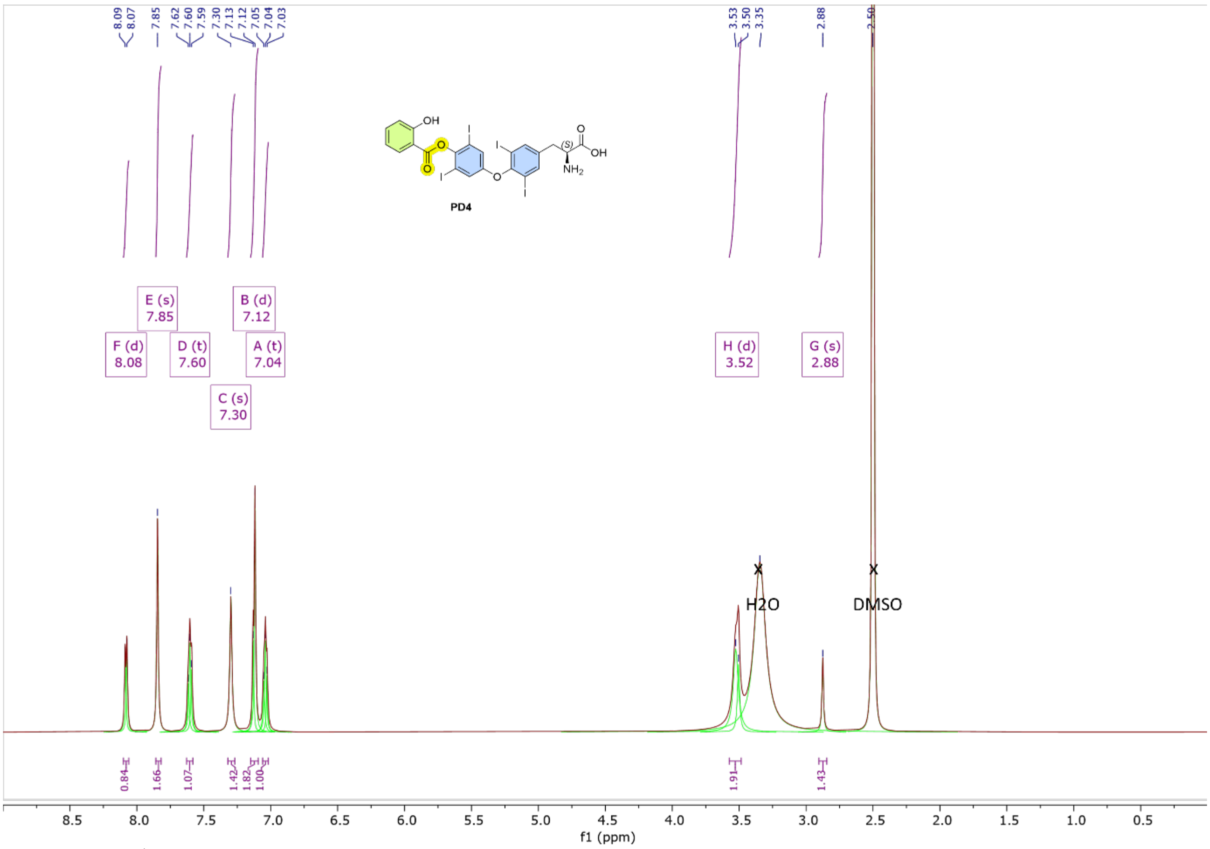

Figure S28. ^{1}H NMR spectrum of prodrug **4**.

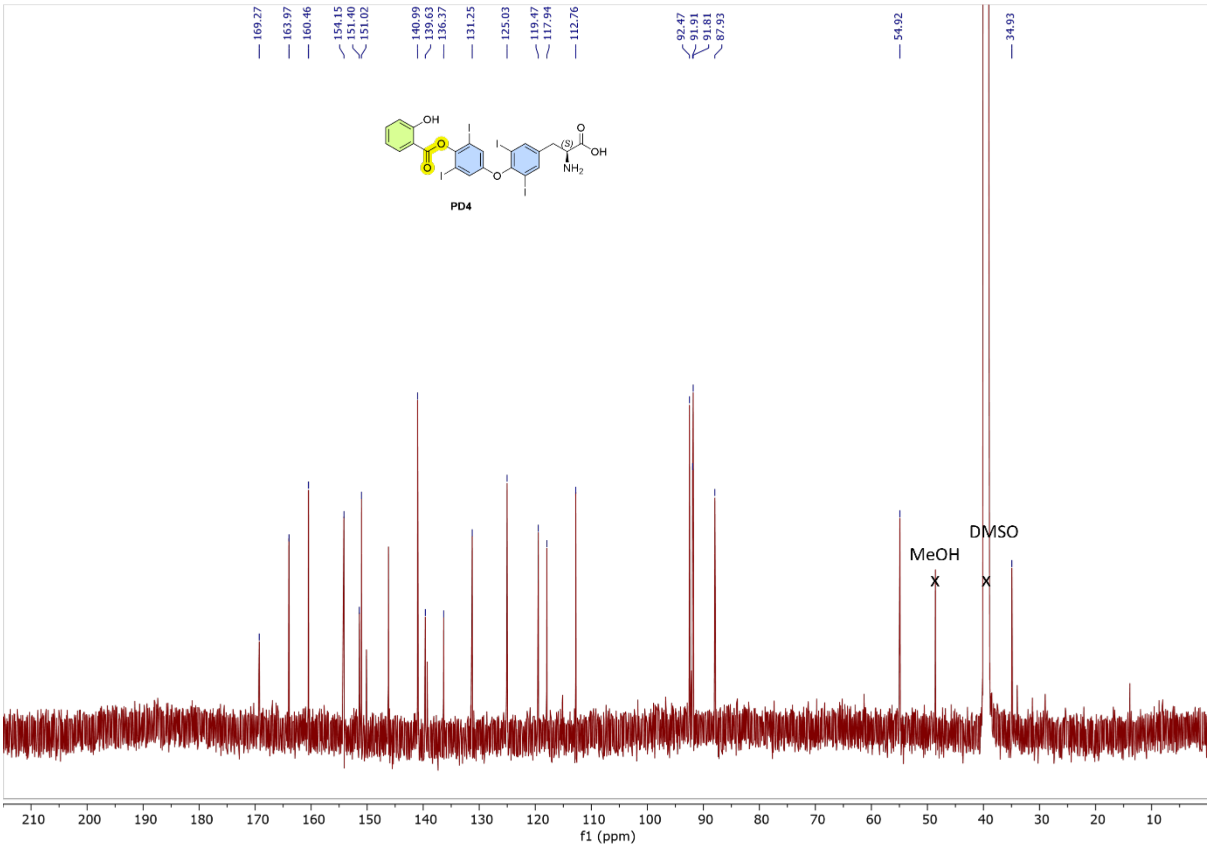

Figure S29. ^{13}C NMR spectrum of prodrug **4**.

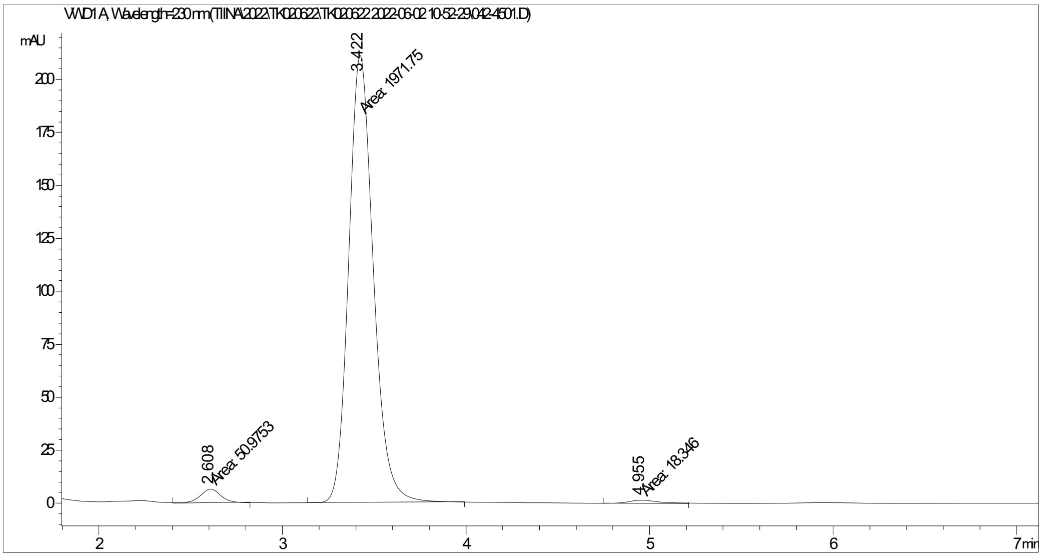

Figure S30. HPLC chromatogram of prodrug **5** (purity 96.66%).

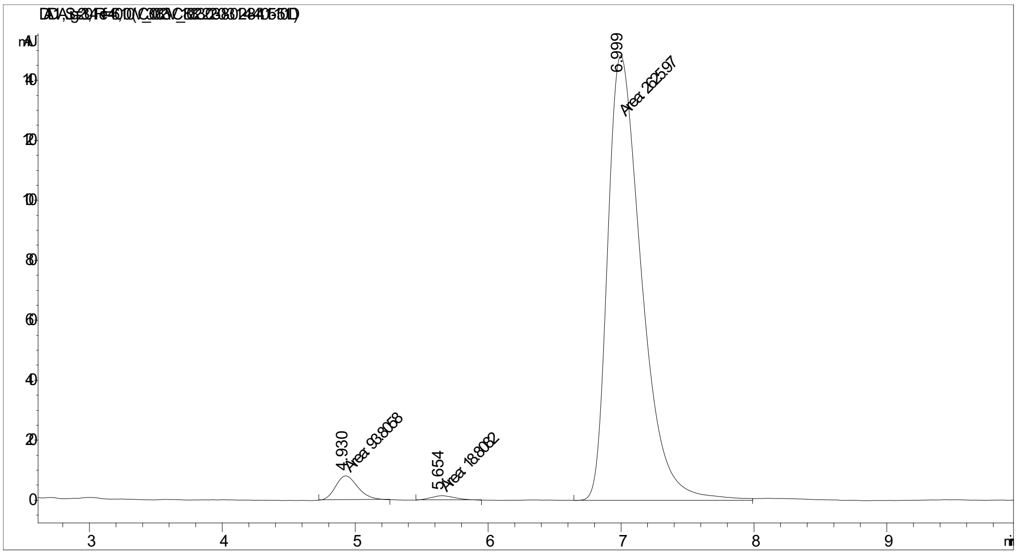

Figure S31. HPLC chromatogram of prodrug **6** (purity 95.89%).

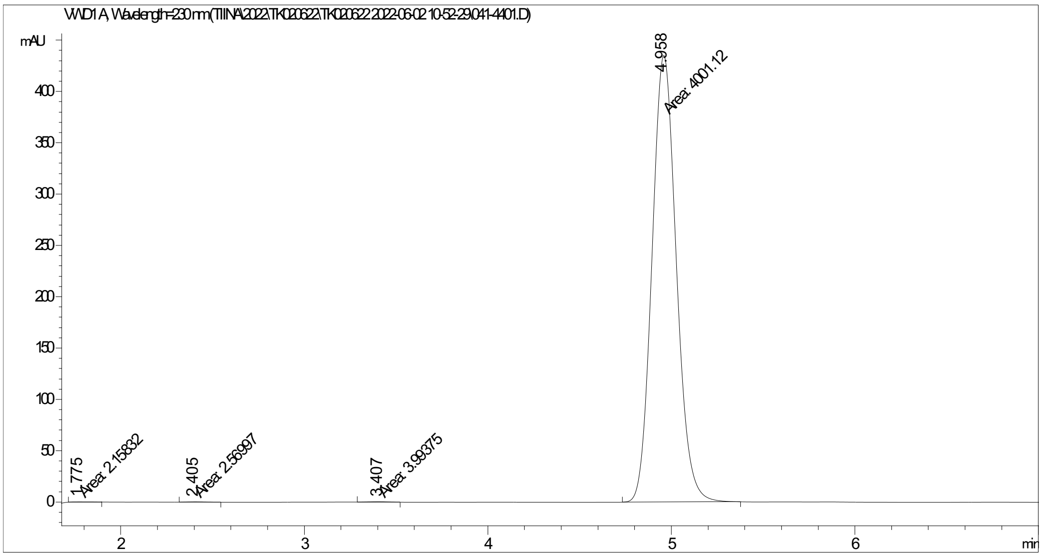

Figure S32. HPLC chromatogram of the parent drug for prodrug **5-6**, commercial naproxen (purity 99.78%).

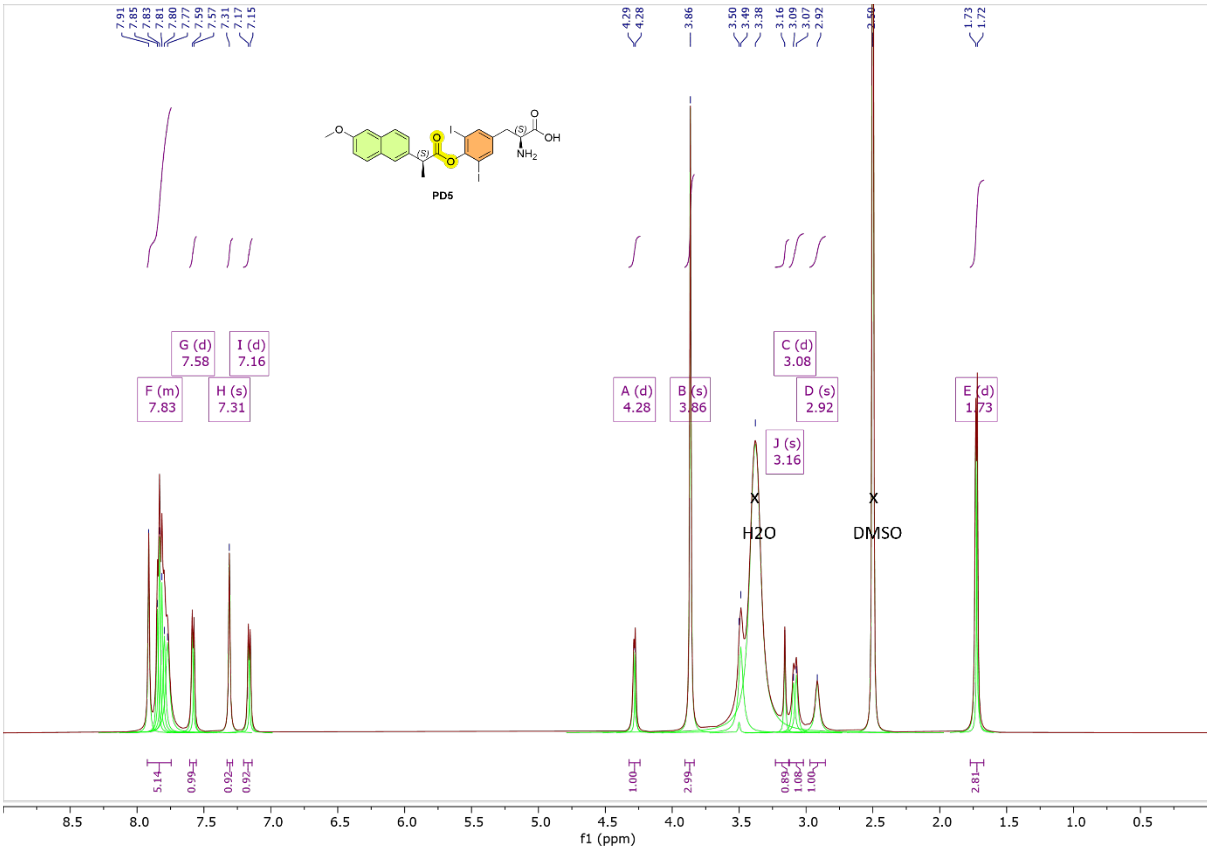

Figure S33. ^{1}H NMR spectrum of prodrug **5**.

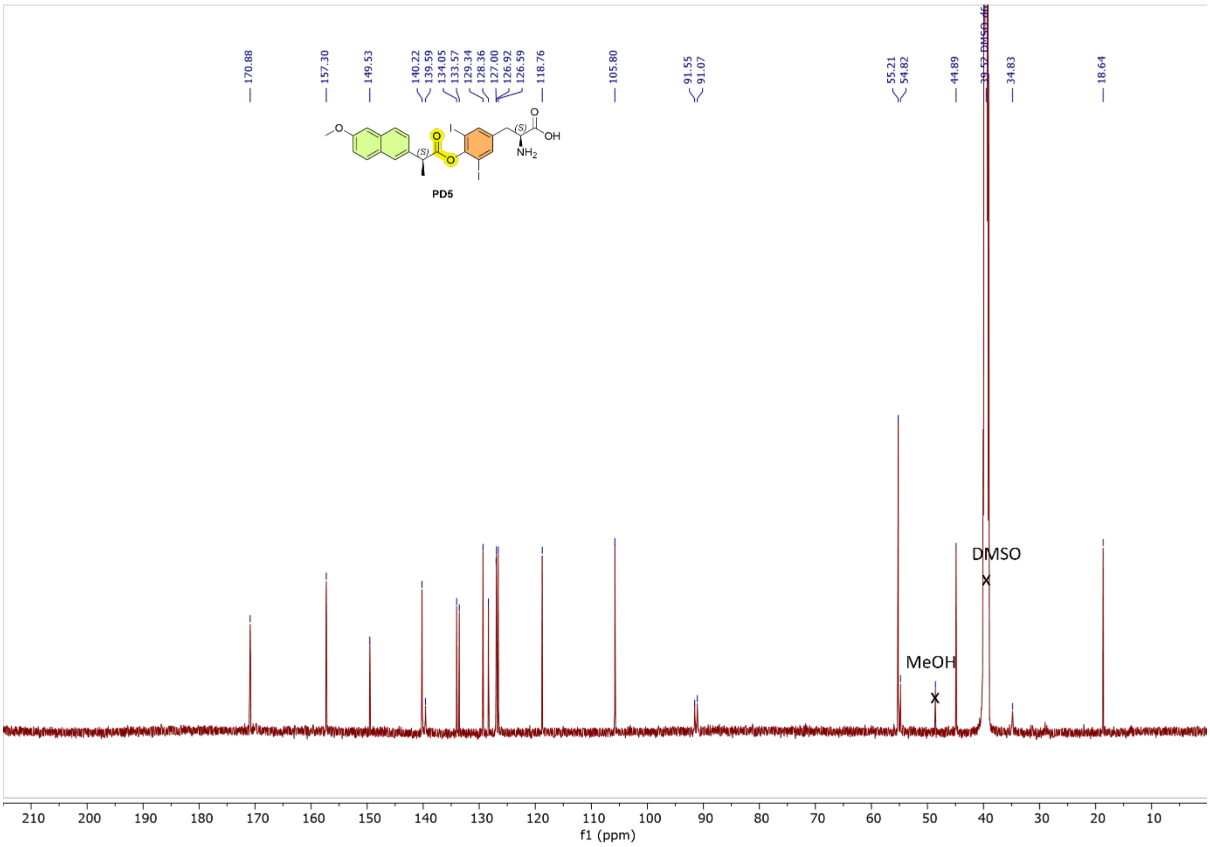

Figure S34. ^{13}C NMR spectrum of prodrug **5**.

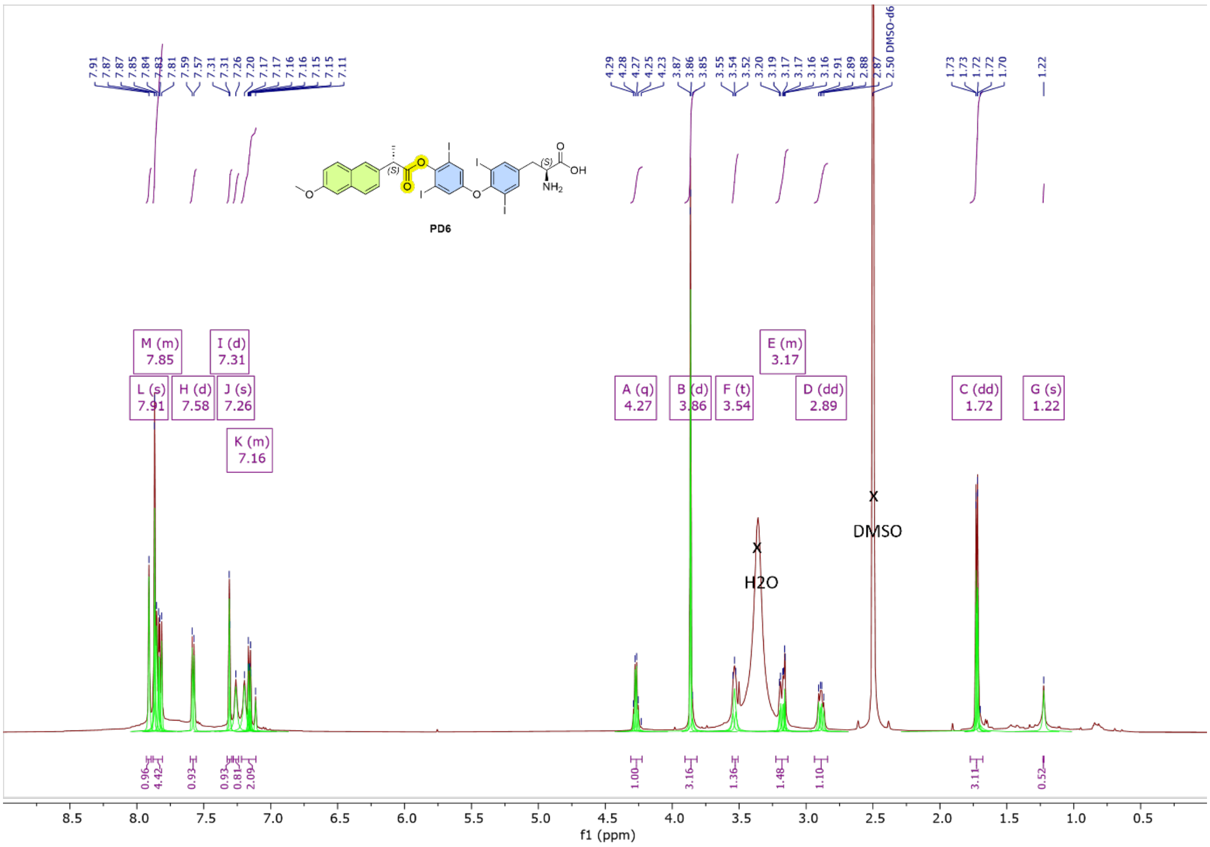

Figure S35. ^{1}H NMR spectrum of prodrug **6**.

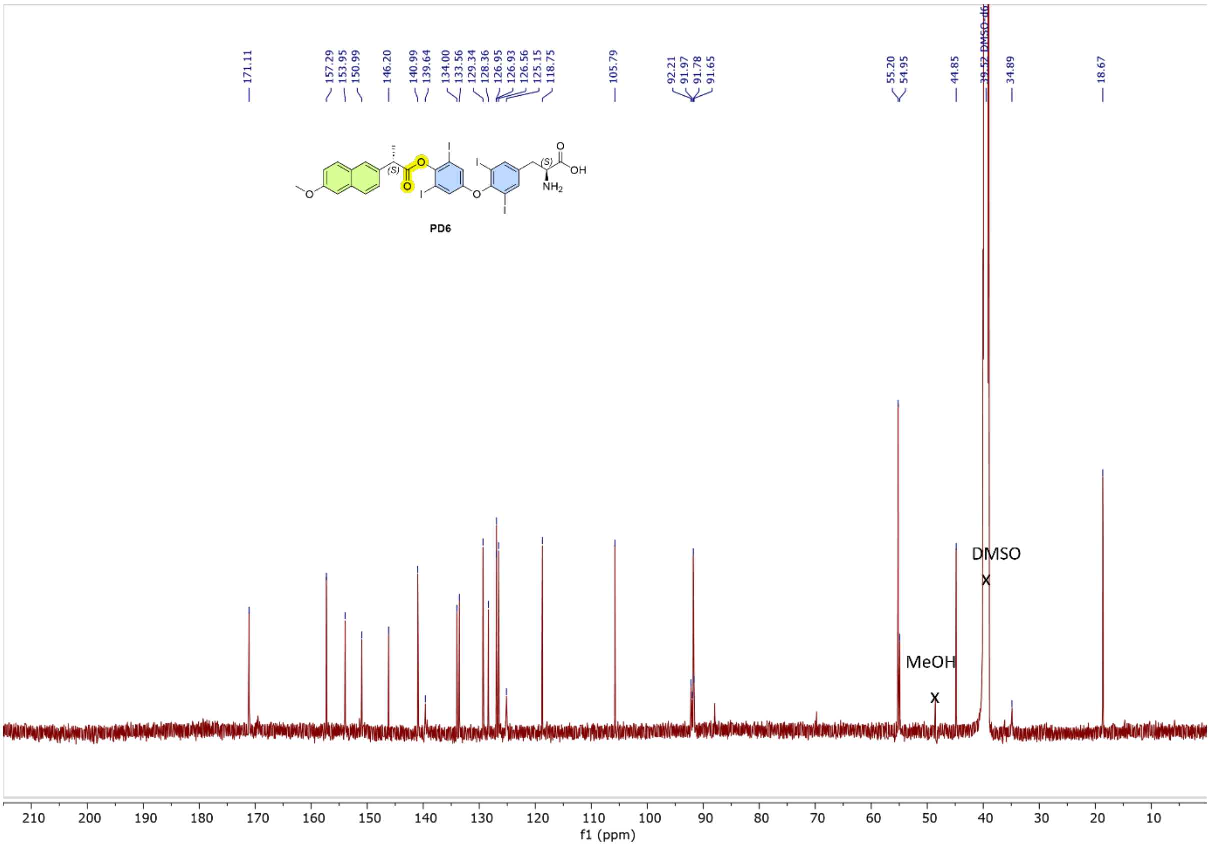

Figure S36. ^{13}C NMR spectrum of prodrug **6**.

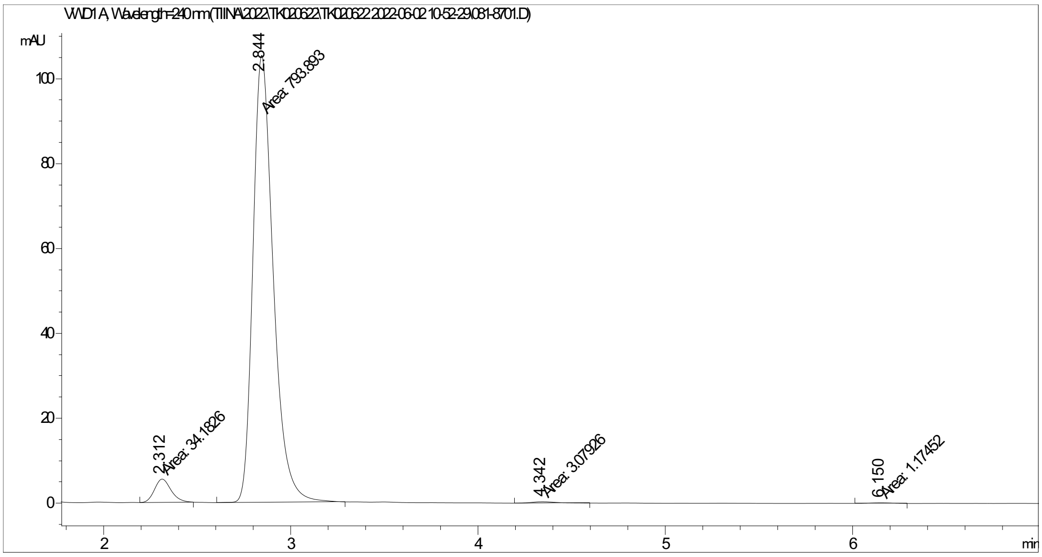

Figure S37. HPLC chromatogram of prodrug **7** (purity 95.38%).

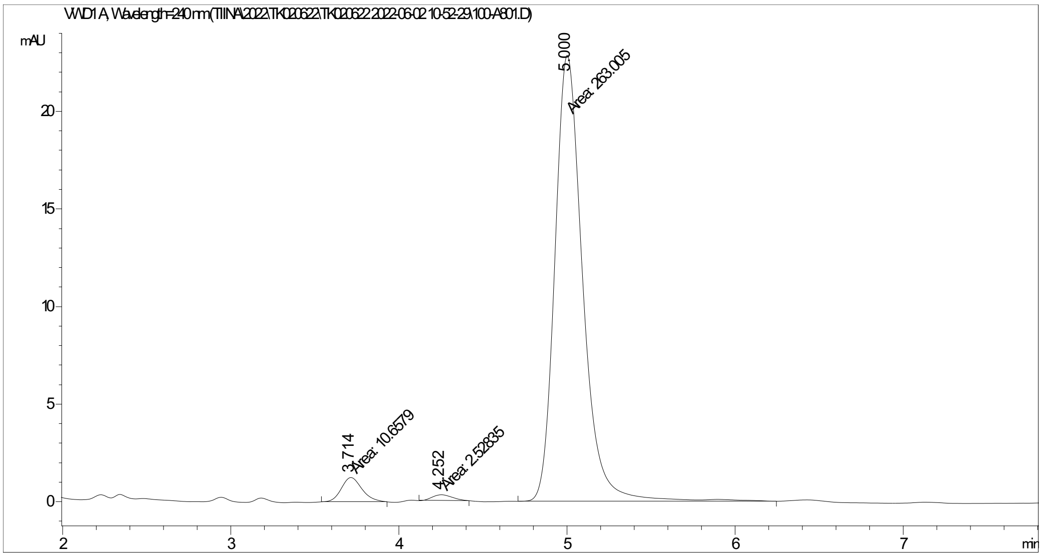

Figure S38. HPLC chromatogram of prodrug **8** (purity 95.18%).

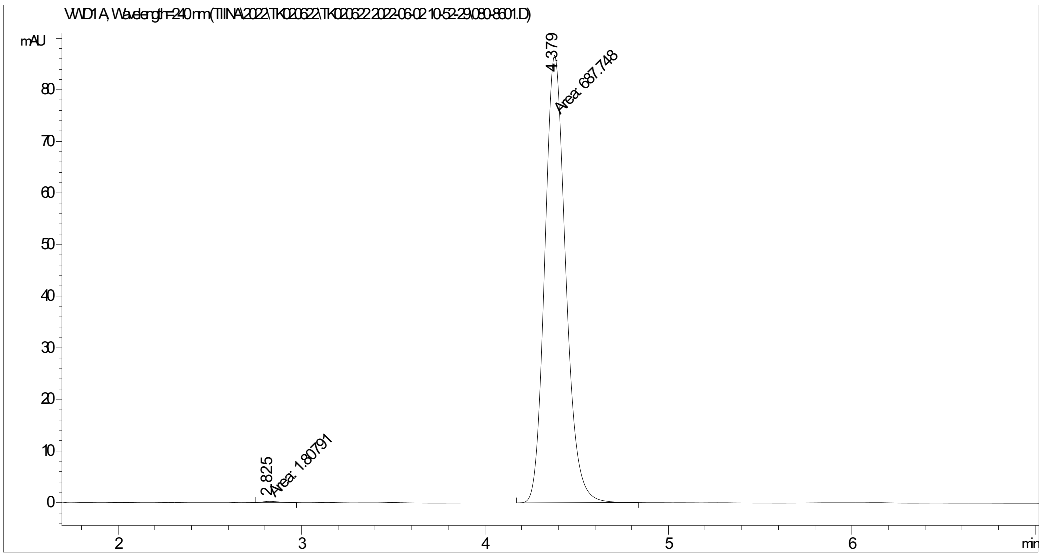

Figure S39. HPLC chromatogram of the parent drug for prodrug **7-8**, commercial flurbiprofen (purity 99.75%).

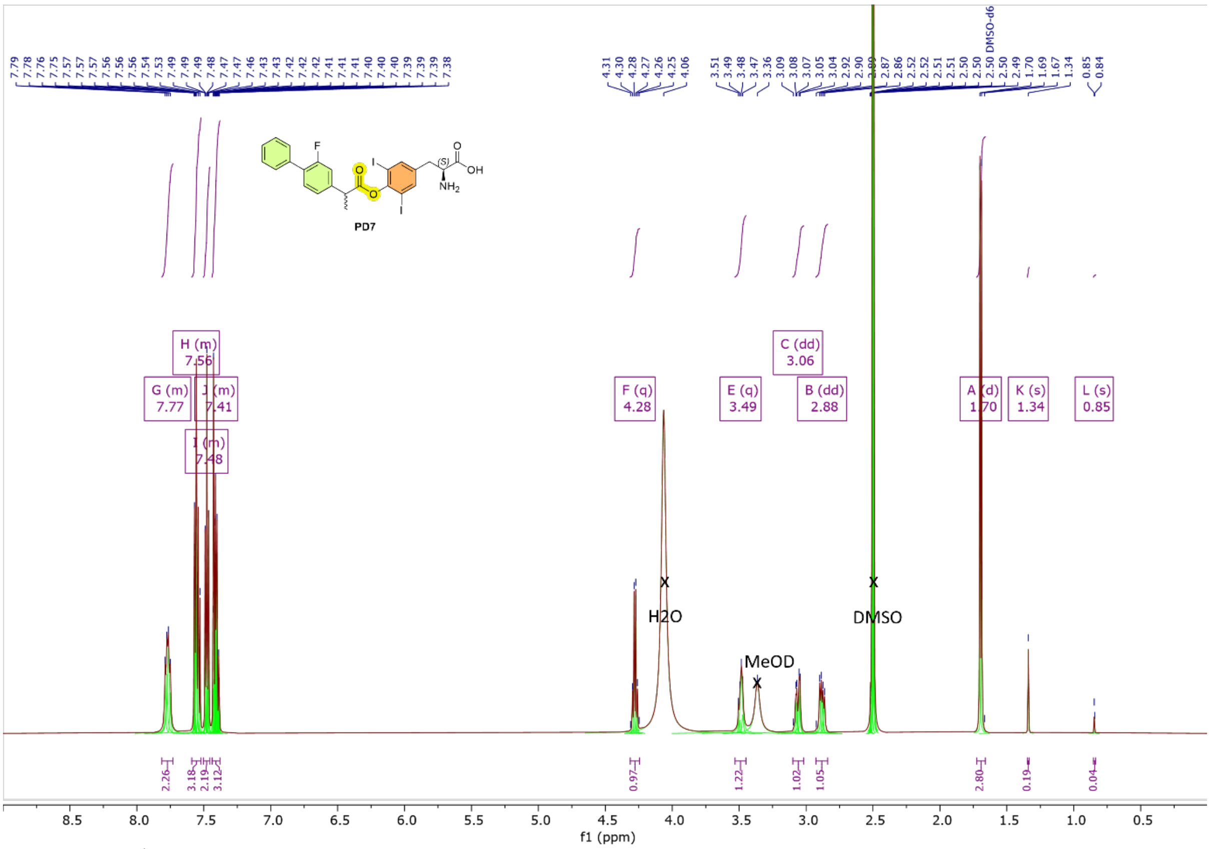

Figure S40. ^{1}H NMR spectrum of prodrug **7**.

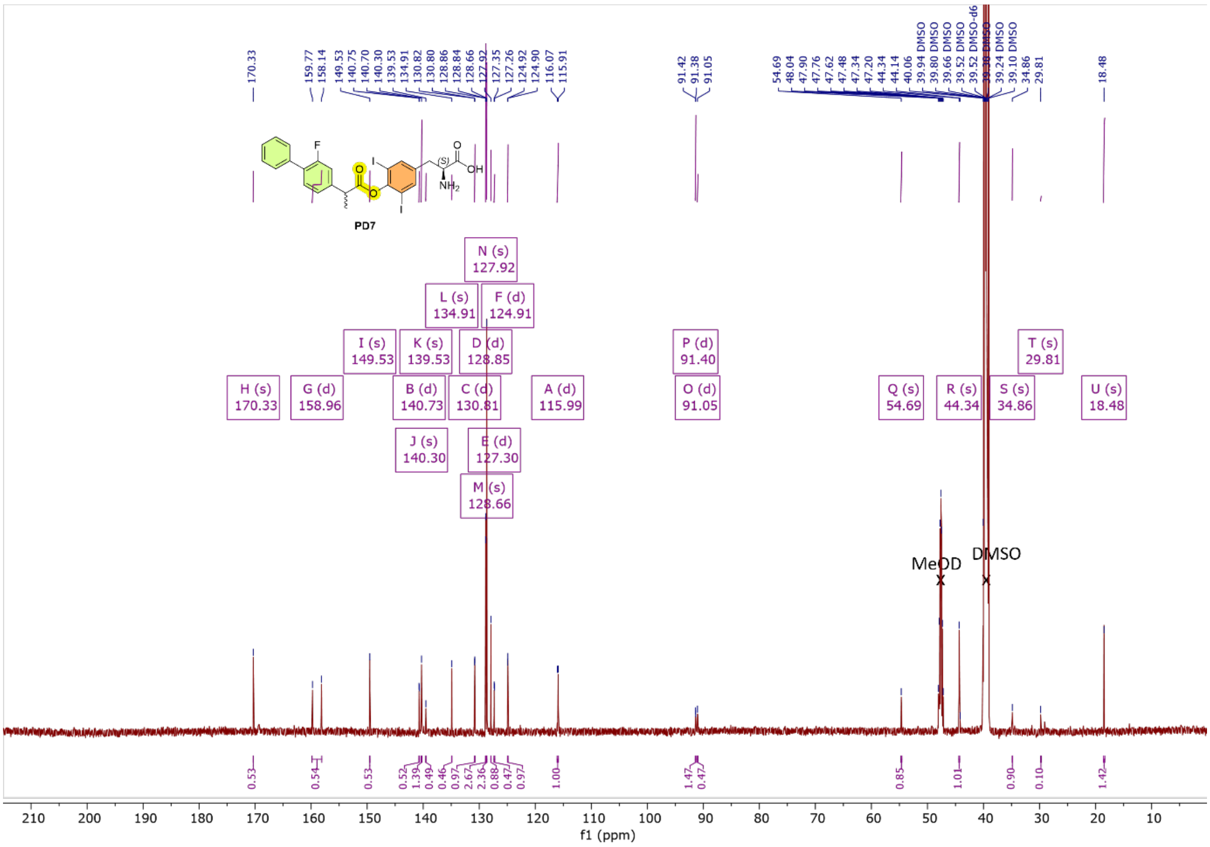

Figure S41. ^{13}C NMR spectrum of prodrug **7**.

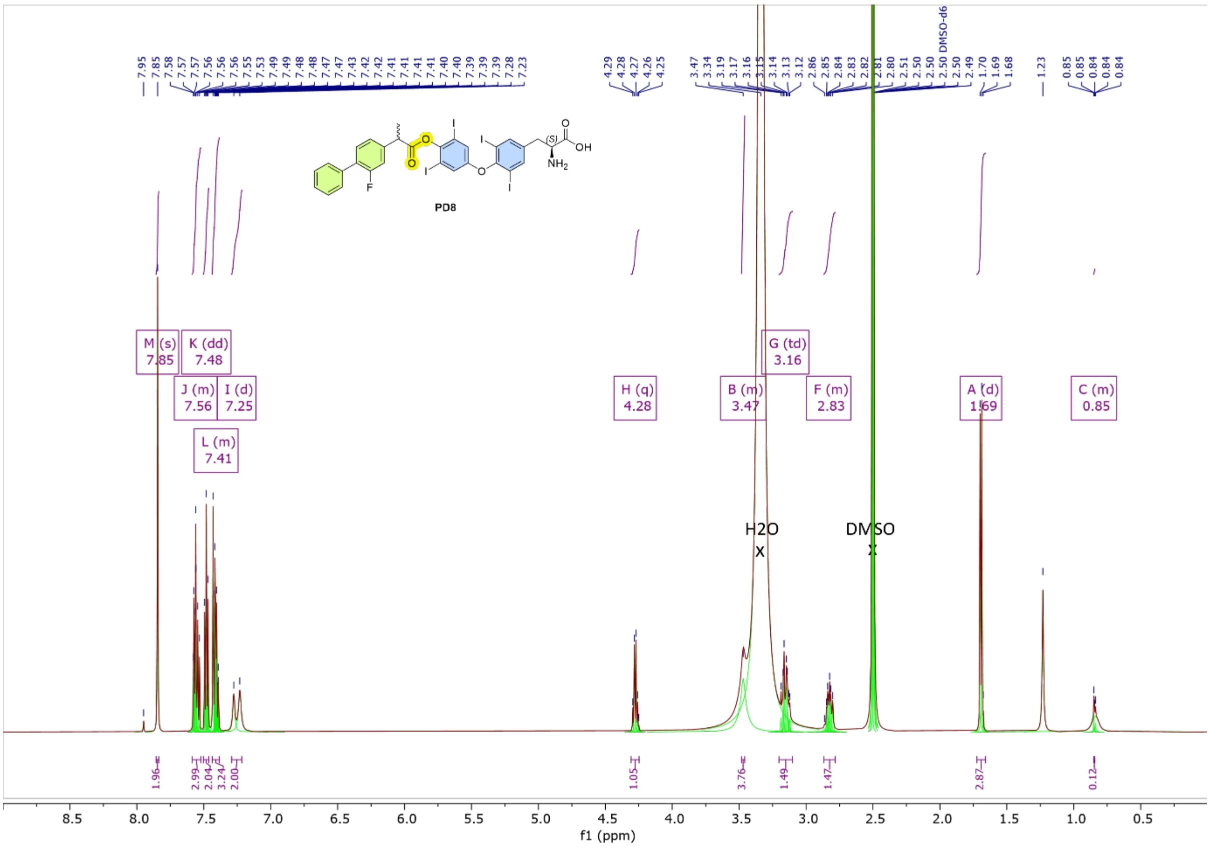

Figure S42. ^{1}H NMR spectrum of prodrug **8**.

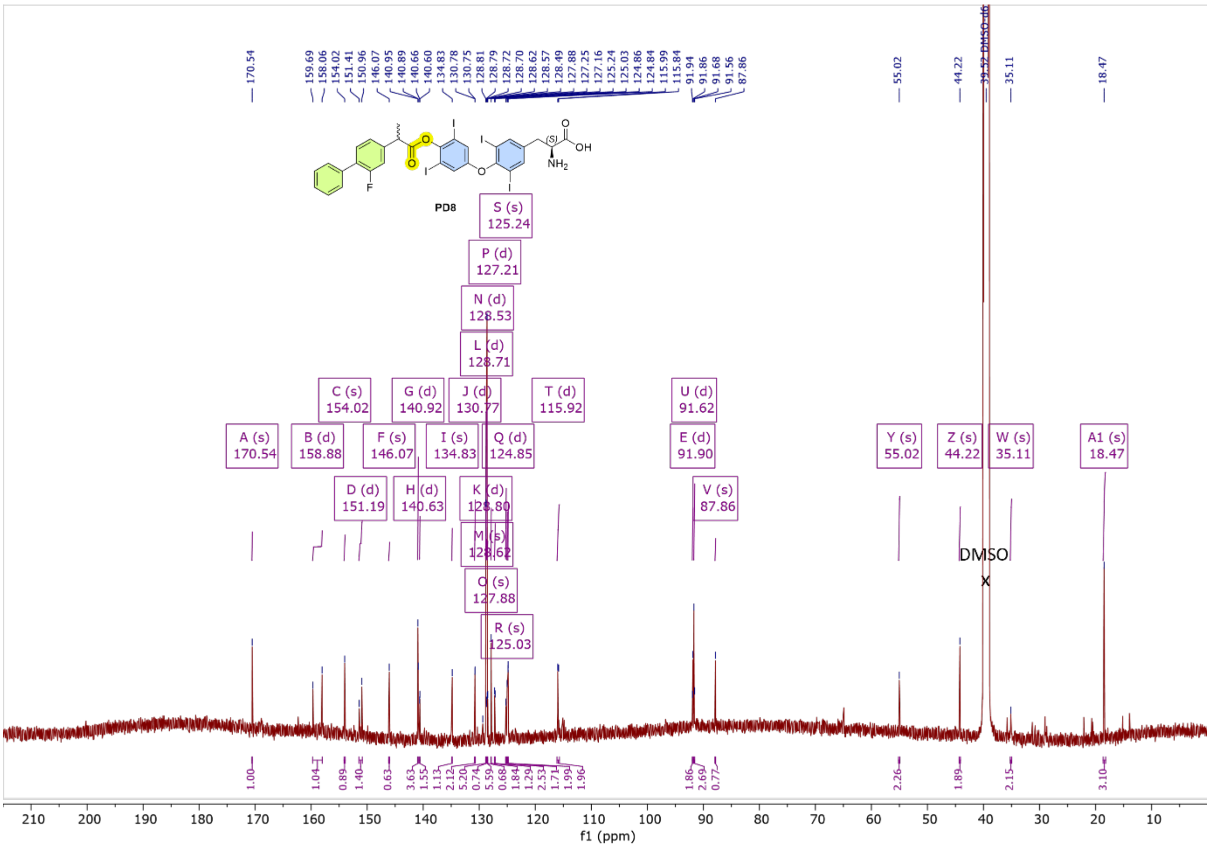

Figure S43. ^{13}C NMR spectrum of prodrug **8**.

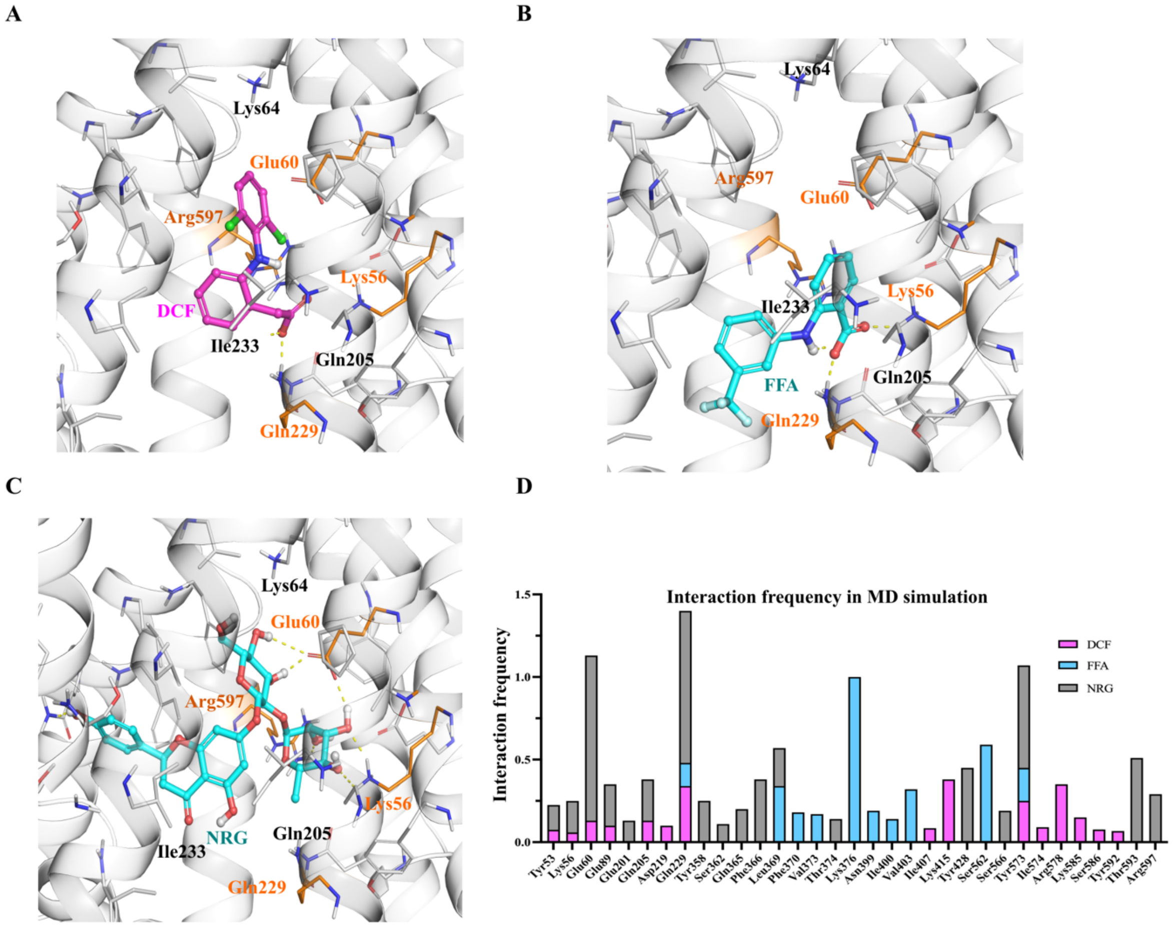

Figure S44. Docking poses of DCF (A), FFA (B) and NRG (C) in OATP1C1. D) Interaction frequencies observed in 2500ns molecular dynamics simualtions for each inhibitor compound.

References:

1. Adla, S. K. *et al.* Neurosteroids: Structure-Uptake Relationships and Computational Modeling of Organic Anion Transporting Polypeptides (OATP)1A2. *Molecules* **26**, 5662 (2021).

2. Shan, Z. *et al.* Cryo-EM structures of human organic anion transporting polypeptide OATP1B1. *Cell Res.* (2023) doi:10.1038/s41422-023-00870-8.
